# Involvement of IDA-HAE Module in Natural Development of Tomato Flower Abscission

**DOI:** 10.3390/plants12010185

**Published:** 2023-01-01

**Authors:** Lu Lu, Samiah Arif, Jun Myoung Yu, June Woo Lee, Young-Hoon Park, Mark Leo Tucker, Joonyup Kim

**Affiliations:** 1 Department of Horticultural Science, Chungnam National University, Daejeon 34134, Republic of Korea; 2 Department of Applied Biology, Chungnam National University, Daejeon 34134, Republic of Korea; 3 Department of Horticultural Bioscience, Pusan National University, Miryang 50463, Republic of Korea; 4 Quality Assurance Team, Quality Assurance Department, Nongwoobio Co., Ltd., Yeoju 12655, Republic of Korea; 5 Soybean Genomics and Improvement Lab, Agriculture Research Service, United States Department of Agriculture, Building 006, BARC-West, Beltsville, MD 20705, USA

**Keywords:** tomato, flower abscission, stress, inflorescence deficient in abscission, HAESA, bimodal role

## Abstract

The *unwanted* detachment of organs such as flowers, leaves, and fruits from the main body of a plant (abscission) has significant effects on agricultural practice. Both timely and precise regulation of organ abscission from a plant is crucial as it influences the agricultural yield. The tomato (*Solanum lycopersicum*) has become a model system for research on organ abscission. Here, we characterized four tomato natural abscission variants named jointless (j), functionally impaired jointless (fij), functionally impaired jointless like (fij like), and normal joint (NJ), based on their cellular features within the flower abscission zones (AZ). Using eight INFLORESCENCE DEFICIENT IN ABSCISSION (SlIDA) genes and eight HAESA genes (SlHAE) identified in the genome sequence of tomato, we analyzed the pattern of gene expression during flower abscission. The AZ-specific expression for three tomato abscission *polygalacturonases* (SlTAPGs) in the development of flower AZ, and the progression of abscission validated our natural abscission system. Compared to that of j, fij, and fij like variants, the AZ-specific expression for SlIDA, SlIDL2, SlIDL3, SlIDL4, and SlIDL5 in the NJ largely corelated and increased with the process of abscission. Of eight SlHAE genes examined, the expression for *SlHSL6* and *SlHSL7* were found to be AZ-specific and increased as abscission progressed in the NJ variant. Unlike the result of gene expression obtained from natural abscission system, an in silico analysis of transcriptional binding sites uncovered that SlIDA genes (SlIDA, SlIDL6, and SlIDL7) are predominantly under the control of environmental stress, while most of the SlHSL genes are affiliated with the broader context in developmental processes and stress responses. Our result presents the potential bimodal transcriptional regulation of the tomato IDA-HAE module associated with flower abscission in tomatoes.

## 1. Introduction

Plants can shed their vegetative and reproductive organs when developmental and environmental cues are available [1]. In plants, the unique cell separation process known as abscission, or organ separation, occurs [2,3]. It is a biological process that is mainly controlled by hormones such as ethylene and auxin. Other hormones that include cytokinins, gibberellins, jasmonic acid, and abscisic acid, also have a role in the commencement of abscission [4,5,6]. As premature abscission in agricultural settings dramatically reduces crop productivity, controlling the act of abscission can offer economic value. Over the past decades, plant species such as soybean, tomato, and Arabidopsis have served as the model systems for abscission research of leaf petiole, flower pedicel, and floral organs, respectively [7].

Abscission takes place in a particular cell type called the abscission zone (AZ), identifiable in the organs to be detached from a plant. The typical AZ cells are small, cytoplasmically dense, and less vacuolated, which comprise several to many layers depending on the species [2]. In addition to developmental cues such as pollination, diverse stress signals associated with temperature, light, water, nutrient, and pathogen have been found to bring about a series of subsequent events that eventually lead to separation.

Arabidopsis does not display leaf or fruit abscission under normal conditions [3]. However, recent genetic and biochemical studies using Arabidopsis floral organs abscission have enhanced our understanding of the regulation of abscission. As part of the process, four phases have been modeled [3,8,9]: (1) the differentiation of undifferentiated cells into an anatomically distinct AZ; (2) AZ cells becoming capable of responding to signals of abscission; (3) activation of the AZ cells, causing the cell walls to be loosened by cell wall modifying enzymes and forming an extensible boundary layer on the separating cells, which eventually culminate in organ separation; and (4) the remnant part of the AZ that undergoes transdifferentiation to create a layer of protection.

It has been shown that a variety of hormones have roles in initiating and modulating several types of abscissions. Of these hormones, ethylene is known to be essential in soybean leaf and tomato flower abscission [7,10,11,12]. Prior studies have demonstrated the role of ethylene in modulating diverse abscission systems. For instance, a transcriptomic investigation of an auxin-depleted tomato (*Solanum lycopersicum*) flower AZ system has revealed that the ethylene sensitivity and activity of associated regulatory signaling components in the AZ, including WRKY and bZIP transcription factors (TF), are elevated during abscission [11]. Importantly, pretreatment with the ethylene action inhibitor 1-methylcyclopropene (1-MCP) repressed the elevated activities [11], corroborating the fact that ethylene is essential in flower abscission of the tomato.

In addition to ethylene, a short peptide known as inflorescence deficient in abscission (*IDA*) in Arabidopsis (*AtIDA*), has been identified as a crucial signaling component in the regulation of Arabidopsis floral organ abscission [9,13,14]. The later stages of Arabidopsis floral organ abscission, phases three to four, are the proposed phases for the activity of *AtIDA* [8,9,15]. The recent identification of IDA homologs in other crop species such as soybean and tomato [12], and the correlative expression during three different abscission systems of soybean leaf, tomato flower, and Arabidopsis floral organs [7], further implied functional relevance for the role of IDA in abscission of various crop species.

The original Arabidopsis *ida* mutant displays the defect in floral organ abscission [13], and this abscission defect in the IDA was substantially restored by the ectopic expression of homologous IDA like genes (e.g., *AtIDL1*-*5*) [14]. Several studies have demonstrated that the complementation using the 35S promoter-driven citrus IDA3 gene and AtIDA promoter-driven litchi IDL1 gene was able to recover the defect in the Arabidopsis IDA mutant [16,17], suggesting the functional conservence. Recent biochemical and genetic approaches in Arabidopsis have revealed that the subtilisin like proteinase (SBTs) family members, SBT4.12, SBT4.13, and SBT5.2, cleave between a lysine and a glycine residue of the (E)PIP motif to produce a mature IDA peptide that is 14 amino acids long [18]. In addition, it has been reported that post-translational modification, such as hydroxylation on proline residues of the (E)PIP motif, is crucial for the recognition of the AtIDA ligand by its cognate receptors [6,19].

Of the more than 600 members identified in Arabidopsis [20], HAESA (AtHAE), HAESA like 1 (AtHSL1), and HAESA like 2 (AtHSL2) are leucine-rich repeat receptor like kinases (LRR-RLK) uncovered for receptors to transduce AtIDA related signals in developmental processes such as floral organ abscission, lateral root emergence, and leaf epidermal cell patterning [14,19,21,22,23]. AtHAE and AtHSL1/2 belong to the LRR RLK X1 subfamily [24], and the earlier study of gene expression for AtHAE in floral organ abscission accompanied with delayed abscission phenotype in the transgenic line governed by the suppression of AtHAE [25] have set out the ligand-receptor pair relationship in Arabidopsis. Findings that AtHAE and AtHSL2 are redundantly required for the receptor function in Arabidopsis and that they act epistatic to AtIDA genetically [14,21] and further established IDA-HAE/HSL as the signaling module in the control of floral organ abscission in Arabidopsis.

In the past two decades, there has been much progress in our understanding of IDA’s developmental role in the control of floral organ abscission in Arabidopsis; however, molecular insights into the role of IDA homologs in the abscission of agricultural crops are still elusive. With the recent advent of gene editing tools such as CRISPR-Cas9, it is more feasible to study gene function associated with agronomic traits such as flower abscission in the tomato. To understand the role of IDA in the development of flower abscission in tomatoes, we investigated the involvement of the tomato IDA signaling module. We identified tomato IDA (SlIDA) and HAE (SlHAE) homologs by searching the tomato genome sequence and re-evaluating recent transcriptome data [7,11,12,26]. In contrast to those prior explant systems to study abscission, in which major auxin sources, including leaf blade and flower, are removed to accelerate the abscission process [7,11,12], we examined the role of SlIDA and SlHAE genes in the natural development of flower abscission. Using four types of natural abscission variants in the tomato, we analyzed the pattern of gene expression for SlIDA and SlHAE homologs during the natural developmental stages of abscission. Further, we examined the cisregulatory elements for the promoter sequences of SlIDA and SlHAE homologs and compared our current findings with the prior auxin-depleted flower abscission system in tomato. Our data suggest that the tomato IDA-HAE module is transcriptionally subject to both the developmental process and the stress responses associated with flower abscission in the tomato.

## 2. Results and Discussion

Tomato is an excellent model plant to study the abscission of flowers and leaves [27]. In addition to a wealth of genome sequence information [26,28,29,30], the well-studied interactive roles of ethylene and auxin in the regulation of abscission [11,31,32], and the cellular and physiological processes within the AZ, make it an attractive system to study abscission [33]. Arguably, the gained knowledge on the regulation of tomato pedicel abscission can be mutually translated between other economically important crops [7,15,34] that will allow a fine-tuned regulation of species (e.g., tomato or soybean) or organ-specific (e.g., flower, leaf, or fruit) abscission.

The flower AZ of tomato is well-defined at anatomical levels [35,36,37], and it is characterized as a circumferential knuckle region identifiable in the near midpoint of the flower pedicel. Chemical modifications leading to a breakdown of the primary cell wall matrix spatially confined in the AZ eventually render organ separation [27,35,38,39]. While prior studies have enhanced our understanding of the identification of molecular components and their regulatory roles underlying tomato flower abscission [36,37,40,41,42,43,44], the functional relevance of tomato homologs of IDA signaling genes in the development of flower abscission is still unclear. Given the vast amount of information obtained from Arabidopsis research in the last 20 years, there is still a lack of much information on the role of the IDA-HAE module in the tomato developmental program, including flower abscission.

**Figure 1:**
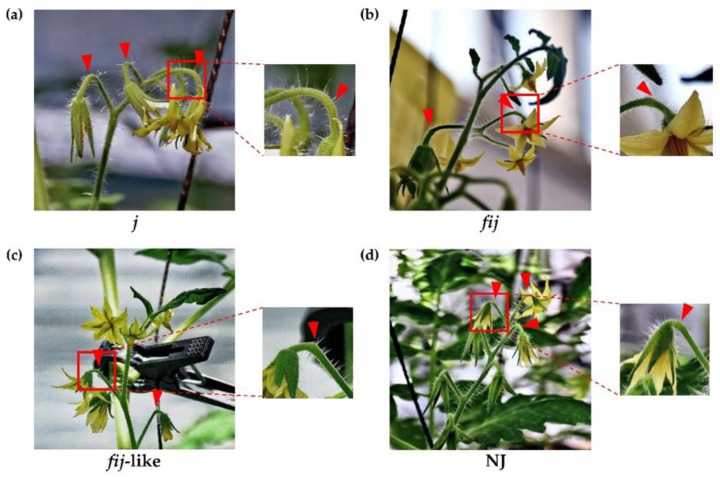
Phenotype of abscission zones (AZ) in four tomato abscission variants. (**a**) jointless (j), (**b**) functionally impaired jointless (fij), (**c**) functionally impaired jointless like (fij like), and (**d**) normal joint (NJ). Various flower developmental stages, except for the j, were used to obtain samples of abscission zones (AZs) and nonabscission zones (NAZs). Arrowheads indicate the junction of AZs in each variant.

**Figure 2:**
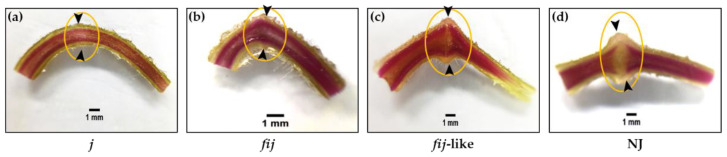
Phloroglucinol staining for lignification of AZs in (**a**) jointless (j), (**b**) functionally impaired jointless (fij), (**c**) functionally impaired jointless like (fij like), and (**d**) normal joint (NJ) pedicels. The unstained cleft was observed to be greater for the NJ variant, signifying the unlignified layers of cells in the AZ compared to fij like, whereas the pattern of lignification in the vascular system of the j and fij pedicels remained unaltered throughout the pedicel. Arrowheads indicate the junction of AZs in each variant. Bar = 1 mm.

To understand the possible role of the IDA-HAE module in tomato flower abscission, we utilized various tomato abscission variants (Figure 1 and Figure S1). Cellular analysis within the AZ allowed us to differentiate four types of natural abscission variants: the well-known jointless (j), functionally-impaired jointless (fij), functionally-impaired jointless like (fij like), and normal joint (NJ) (Figure 2 and Figure S1). Then we extracted RNA from samples of abscission zones (AZs) and nonabscission zones (NAZs, proximal side of pedicel), except for the j, collected from various flower developmental stages for the AZ-specificity of gene expression (i.e., transcript of AZ compared to that of NAZ) (AZ/NAZ). For the j variant, we collected RNA samples from the midpoint of pedicels, as it lacks the joint, where abscission would typically take place in the NJ. For fij and fij like variants, as we named, as they display a knuckle like structure with different degrees of lignification within the AZ, we collected RNA samples from both AZ and NAZ of several flower developmental stages. With the sixteen homologs we identified for tomato SlIDA and SlHAE genes [7,11,12,26], we profiled the gene expression of the identified IDA-HAE module during the progression of flower abscission.

### 2.1. Lignin Deposition in Four Tomato Variants of Abscission Zones

We identified four types of natural abscission variants based on the external morphology related to the AZ formation and response/sensitivity to break or shed of the distal part of pedicels when slightly touched mechanically at the AZ. Since the physiological responses to break or shed in the distal part of pedicels differed for these variants, we decided to examine the cellular characteristics associated with the degree of lignification within the AZ of j, fij, fij like, and NJ variants (Figure 2). The flower AZ of the tomato enlarges toward flower development and fruit maturation, showing its typical knuckle like structure in the pedicel. As for abscission related to tomato pedicel, two temporal abscission processes are largely observed: flowers of abscission resulting from unsuccessful pollination and fruit of abscission past over-ripening [45]. It has been demonstrated that the pattern of lignification is restricted within the vascular system mainly in the xylem before or around the fruit set, within the vascular system, and across the transverse region of the AZ [44,45,46,47].

When the lignification within the flower AZ of j, fij, fij like, and NJ, variants were evaluated, the pattern of lignification of all the variants mostly remained within the vascular system throughout the pedicels (Figure 2). In particular, where the pattern of lignification in the vascular system of j and fij pedicels remained undisrupted throughout the pedicel, the pattern of vascular staining for the fij like variant presented a slight cleft that mirrored the indentation of AZ (Figure 2a–c). Further, this unstained cleft was found greater than that of the NJ variant, representing the unlignified layers of cells in the AZ (Figure 2d). Although recent studies have revealed the molecular components, such as MADS box genes, associated with the formation of tomato flower AZ [36,37,40,41,42,43,44], it is still not clear if the tomato IDA-HAE module is also related to tomato flower abscission and the formation of the AZ.

### 2.2. Validation of Natural Flower Abscission System

**Figure 3:**
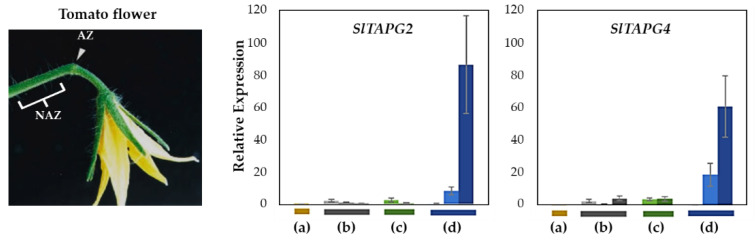
Relative gene expression of SlTAPG2, and SlTAPG4 for the AZ/NAZ in the four tomato variants. (a) jointless (j), (b) functionally impaired jointless (fij), (c) functionally impaired jointless like (fij like), and (d) normal joint (NJ). Except for the j (collected from only the midpoint), samples of AZ and NAZ (proximal side of pedicel) from several flower developmental stages available on the peduncle of the variant that covers a relatively broad range of flower abscission development were collected (Figure S1). One developmental stage of j, three developmental stages of fij, two developmental stages of fij like, and three developmental stages of NJ were collected. The samples from two neighboring peduncles on the primary inflorescence were utilized and combined for the respective developmental stages of AZ and NAZ samples from each peduncle to enrich the samples for RNA extraction. Each bar graph (AZ/NAZ), except for the j, of the natural abscission variant, represents the result from those several flower abscission developmental stages. The means ± SD of three independent replicates were used to present data sets.

To validate our natural flower abscission system, we examined the gene expression of three tomato abscission *polygalacturonases* (SlTAPG2 and SlTAPG4) in four abscission variants, previously identified as marker genes for tomato flower abscission [33] (Figure 3). Gene expression from flower AZs was compared to that from NAZs, except for the j, which represents the AZ-specificity (Figure S1). To cover a relatively broad range of abscission progression, the AZ and NAZ samples from several flower developmental stages available on the peduncle of the variant were utilized. Further, the samples from two neighboring peduncles on the primary inflorescence were collected and combined for the respective developmental stages of AZ and NAZ samples from each peduncle to enrich for RNA extraction (see Materials and Methods for the details).

Gene expression for SlTAPG2 and SlTAPG4 in the j was not detectable. The expression of SlTAPG2 and SlTAPG4 in fij and fij like variants was very low and much more variable throughout the several developmental stages of abscission. This variability may have resulted from the sampling issue that is associated with the indistinct knuckle like structure inherent in those fij and fij like variants (Figure 1, Figure 2 and Figure S1). As expected, gene expression of all three marker genes was found to be the greatest and increasing in the NJ variant as abscission progresses, in line with the result from the explant system (Figure 3 and Figure S2).

### 2.3. Gene Expression Profiling and Cisregulatory Element Analyses of the IDA-HAE Module in Four Tomato Abscission Variants

**Figure 4:**
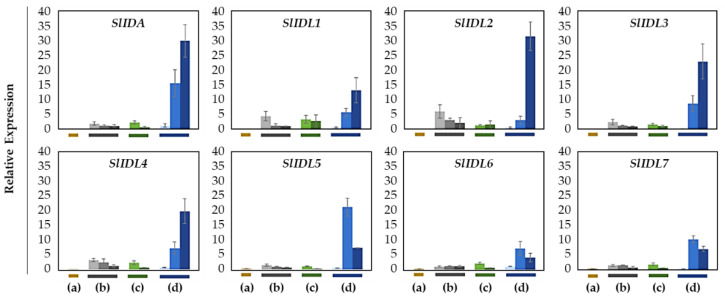
Relative gene expression of SlIDA for the AZ/NAZ in the four tomato variants. (a) jointless (j), (b) functionally impaired jointless (fij), (c) functionally impaired jointless like (fij like), and (d) normal joint (NJ). Except for the j (collected from only the midpoint), samples of AZ and NAZ (proximal side of pedicel) from several flower developmental stages available on the peduncle of the variant that covers a relatively broad range of flower abscission development were collected (Figure S1). One developmental stage of j, three developmental stages of fij, two developmental stages of fij like, and three developmental stages of NJ were collected. The samples from two neighboring peduncles on the primary inflorescence were utilized and combined for the respective developmental stages of AZ and NAZ samples from each peduncle to enrich the samples for RNA extraction. Each bar graph (AZ/NAZ), except for the j, of the natural abscission variant, represents the result from those several flower abscission developmental stages. The means ± SD of three independent replicates were used to present data sets.

Next, to determine if tomato the IDA-HAE module is associated with the development of flower abscission, we performed gene expression analysis for eight SlIDA genes (SlIDA and SlIDL1-SlIDA7) in four tomato abscission variants (Figure 4). In contrast to prior studies that have demonstrated the increased expression for a few tomato IDA genes (Figure S2) related to tomato leaf and flower abscission systems [7,11,12], in which major auxin sources such as leaf blade or flower are removed, or acceleration of abscission process is triggered by ethylene treatment, we analyzed gene expression from the natural flower abscission system with no such pretreatments. We collected AZ and NAZ samples from younger to older flowers on the pedicel in four abscission variants to tie the functional relevance of the IDA-HAE module with both the development of AZ and the progressive stages of abscission (Figure S1).

**Figure 5:**
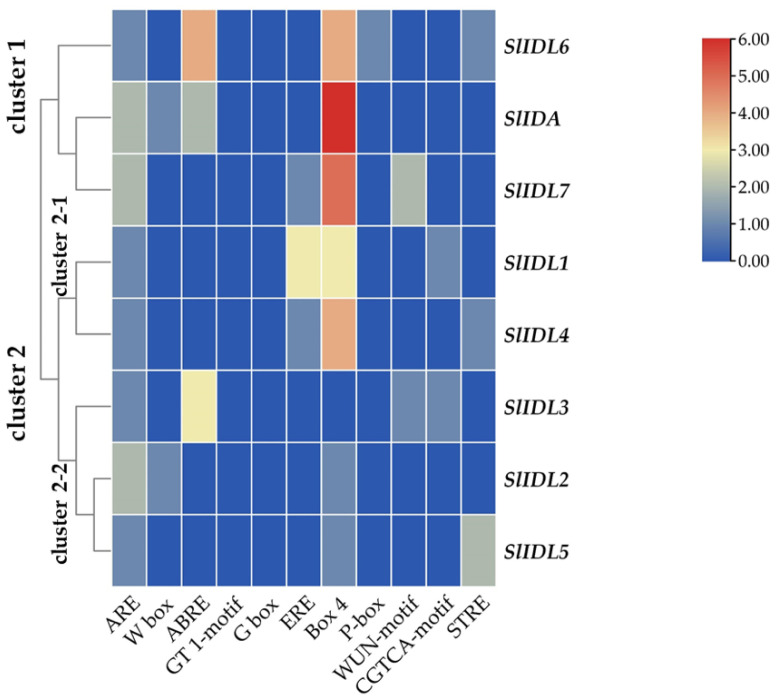
Cisregulatory elements in the promoter regions of SlIDA genes. ARE (anaerobic induction element), W-box (WRKY binding site), ABRE (cisacting element involved in the abscisic acid responsiveness), GT1-motif (light responsive element), G Box (cisacting regulatory element involved in light responsiveness), ERE (ethylene-responsive cisacting regulatory element), Box 4 (part of a conserved DNA module involved in light responsiveness), P-box (gibberellin-responsive element), WUN-motif (wound-responsive element), CGTCA-motif (cisacting regulatory element involved in the MeJA-responsiveness), and STRE (stress response element) are identified in the promoters of eight SlIDA genes. Heatmap clustering for cisregulatory elements of SlIDA genes was generated with the Euclidean distance measurement and complete cluster method. Scale bars on the right indicate the relative enrichment for the identified cisregulatory elements.

In the abscission variant j, there were no related expression changes for any SlIDA genes as to the pedicel AZ development (i.e., the midpoints combined from the first and second pedicels on the peduncle). Gene expression for eight genes (SlIDA, SlIDL1-SlIDA7) was found detectable with variable levels in the fij and fij like variants. Again, it should be noted that the variability, in part, might have to do with sampling from the indistinct knuckle like structure observed in the fij and fij like variants that might have affected the true signaling event (Figure 1, Figure 2 and Figure S1). Among eight SlIDA genes, the expression for the SlIDA was most prominent followed by SlIDL2, SlIDL3, SlIDL4, and SlIDL5 as tomato flower abscission progressed in the NJ variant (Figure 4). As our experimental system is with no pretreatments differing from previous studies (Figure S2) [7,11,12], it would be interesting to see if such pretreatment or environmental stress could influence gene expression of these SlIDA genes. For instance, a recent study demonstrated that the stress-related low light intensity induced ethylene synthesis that in turn increased gene expression of SlIDL6, which eventually promoted flower abscission in the tomato [48]. In fact, when we examined the cisregulatory elements in the promoter sequences for eight SlIDA genes, the most enriched elements were the binding sites for transcription factors associated with stress response (e.g., ABRE, ARE) particularly with the light response (e.g., Box4, G-box, GT1-motif) (Figure 5). This association was identified specifically in the promoter of SlIDA, SlIDL6, and SlIDL7 and grouped in the same cluster while separated from other genes (Figure 5), suggesting that the transcription of these SlIDA genes is predominantly regulated in response to stress conditions.

It has been previously shown that auxin depletion causes similar changes as stress-induced responses including increased ethylene and reactive oxygen species as well as carbohydrate starvation, which affect flower abscission in tomatoes [31]. Interestingly, previous gene expression analysis for tomato flower abscission after removing the auxin source (i.e., flower) has identified three AZ-specific SlIDA genes (SlIDA, SlIDL7, and SlIDL8) in line with our current finding of cisregulatory elements [7,11] (Figure 5 and Figure S2). Of further note, the magnitude of gene expression for SlIDA (SlIDA, SlIDL6, and SlIDL7) and marker genes (SlTAPG2 and SlTAPG4) from the auxin-depleted abscission system revealed much more than those from the current natural abscission system, which may have resulted from a stress response (Figure 3, Figure 4 and Figure S2) [7,11].

In Arabidopsis, two genetically redundant AtHAE and AtHSL2 are demonstrated to be required for the receptor of IDA ligand in floral organ abscission [14,21]. Using gene sequences of AtHAE and AtHSL2, we identified the eight most homologous sequences of SlHAE and SlHSL genes in the tomato genomic database. To further understand the involvement of the tomato IDA-HAE module, we examined the expression for these eight SlHAE genes in four abscission variants, encompassing progressive abscission stages of flower abscission.

**Figure 6:**
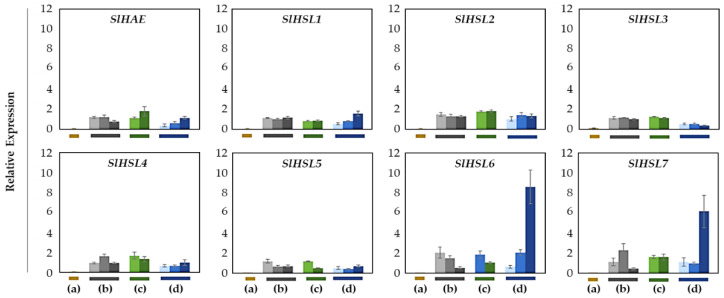
Relative gene expression patterns of SlHAE for the AZ/NAZ in four tomato variants. (a) jointless (j), (b) functionally impaired jointless (fij), (c) functionally impaired jointless like (fij like), and (d) normal joint (NJ). Except for the j (collected from only the midpoint), samples of AZ and NAZ (proximal side of pedicel) from several flower developmental stages available on the peduncle of the variant that covers a relatively broad range of flower abscission development were collected (Figure S1). One developmental stage of j, three developmental stages of fij, two developmental stages of fij like, and three developmental stages of NJ were collected. The samples from two neighboring peduncles on the primary inflorescence were utilized and combined for the respective developmental stages of AZ and NAZ samples from each peduncle to enrich the samples for RNA extraction. Each bar graph (AZ/NAZ), except for the j, of the natural abscission variant, represents the result from those several flower abscission developmental stages. The means ± SD of three independent replicates were used to present data sets.

**Figure 7:**
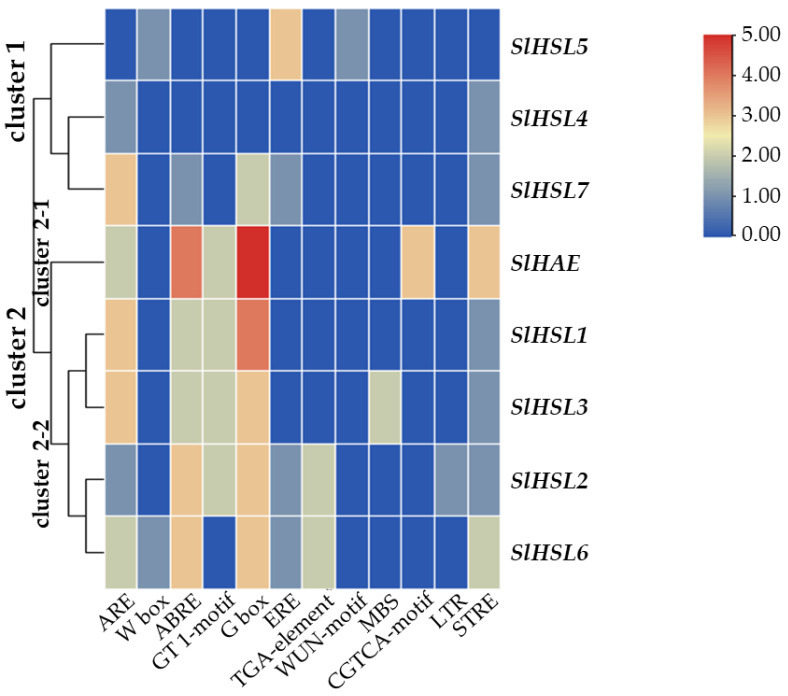
Cisregulatory elements in the promoter regions of SlHAE genes. ARE (anaerobic induction element), W-box (WRKY binding site), ABRE (cisacting element involved in the abscisic acid responsiveness), GT1-motif (light responsive element), G Box (cisacting regulatory element involved in light responsiveness), ERE (ethylene-responsive cisacting), TGA-element (auxin-responsive element), WUN-motif (wound-responsive element), MBS (MYB binding site involved in drought-inducibility), CGTCA-motif (cisacting regulatory element involved in the MeJA-responsiveness), LTR (low-temperature-responsive element), and STRE (stress response element) are identified in the promoters of eight SlHAE genes. Heatmap clustering for cisregulatory of *SlHAE* genes was generated with the Euclidean distance measurement and complete cluster method. Scale bars on the right indicate the relative enrichment for the identified cisregulatory elements.

Overall, most of the gene expression was lower than that of the SlIDA genes in all the variants (Figure 4 and Figure 6). Compared with the pattern of SlIDA genes found in the fij and fij like variants, all the gene expression was very low and variable. Curiously, the expression for tomato homologous genes closely related to AtHSL2 (SlHSL6 and SlHSL7) was found to be most prominent and increased over the progressive stages of the NJ variant (Figure 6). Further, in contrast to the result of cisregulatory elements found in the promoters of SlIDA genes, analysis of the promoters for SlHAE genes revealed the indistinctive clustering of cisregulatory elements for most of the SlHAE genes (Figure 7). In addition, the cisregulatory elements for SlHAE genes identified were more diverse elements linked to many hormone-related (e.g., TGA-element (auxin) CGTCA-motif (meJA), ABRE (ABA), ERE (ethylene)), light, and stress responses (e.g., G-box, GT1-motif, LTR, and STRE) (Figure 7), which implies a broader context of transcriptional regulation for these potential receptors associated with flower abscission in tomato.

## 3. Materials and Methods

### 3.1. Plant Material and Sample Collection

To assess the role of IDA signaling in the process of flower abscission, we used four tomato natural abscission variants: (1) jointless (*j*) (Rio Grande), which has no knuckle like structure and AZ formation, (2) functionally impaired jointless (fij) (LA3963), which has a knuckle like structure but no AZ formation, (3) functionally impaired jointless like (fij like) (Ha7998), which has a knuckle like structure and AZ formation near halfway between fij and normal AZ variants of tomato, and (4) a tomato cultivar with normal joint (NJ) (RomaVF), which has a knuckle like structure and AZ formation (Figure S1).

Except for the j (collected from only the midpoint), we collected AZ and NAZ samples (proximal side of pedicel) from several flower developmental stages available on the peduncle of the variant to cover a relatively broad range of flower abscission development (Figure S1). One developmental stage of j, three developmental stages of fij, two developmental stages of fij like, and three developmental stages of NJ, from Muju Mountain (35°54′44.50″ N, 127°52′ 20.23″ E) in South Korea were collected. To further enrich the samples for RNA extraction, the samples from two neighboring peduncles on the primary inflorescence were utilized and combined for the respective developmental stages of AZ and NAZ samples from each peduncle. The AZ and NAZ samples were collected and immediately frozen using dry ice, and then used for further analysis. Except for the j, each bar graph (transcript compared to that of NAZ and AZ/NAZ) for the natural abscission variant represents the result from those several flower abscission developmental stages (Figure 3, Figure 4 and Figure 6).

### 3.2. RNA Extraction and RT-qPCR Analysis

For RNA extraction, a variety of flower AZ and NAZ samples from four different abscission variants of tomato were processed with the TRIzol reagent (Invitrogen, Life Technologies, Waltham, MA, USA). Recombinant DNase I (TaKaRa, Kyoto, Japan) was used to process the RNA in accordance with the manufacturer’s recommendations. Reverse transcription of the extracted RNA was carried out using the kit (TaKaRa Reverse Transcription) to synthesize cDNA. Using an oligo (dT) primer and reverse transcriptase, 2 µg RNA (20 µL reactions including 4 µL 5× PrimeScript RT Master Mix) was used to create first-strand cDNA after the DNA was removed. RT-qPCR was carried out using a Bio-Rad system (Bio-Rad laboratory, Inc., Hungary Ltd., Budapest, Hungary), in 20 µL reactions with 1 µL of cDNA, 200 nM of each primer, and SuperReal PreMix Plus (Tiangen, Beijing, China). The SYBR SuperReal PreMix plus manufacturer procedure for amplification reactions and conditions were used to detect the mRNA of SlTAPG2 (Solyc02g067640.2.1), SlTAPG4 (Solyc12g096750.1.1), SlIDA (Solyc05g010000.1.1), SlIDL1 (CP023762.1), SlIDL2 (CP023760.1), SlIDL3 (Solyc07g044890.1.1), SlIDL4 (Solyc05g007040.1.1), SlIDL5 (XM_010314166), SlIDL6 (Solyc06g050140.1.1), SlIDL7 (Solyc09g005780.1.1), SlHAE (Solyc02g077630.2.1), SlHSL1 (Solyc03g006300.1.1), SlHSL2 (Solyc07g053600.2.1), SlHSL3 (Solyc04g077010.2.1), SlHSL4 (Solyc04g077010.2.1), SlHSL5 (Solyc02g091860.2.1), SlHSL6 (Solyc08g066270.1.1) and SlHSL7 (Solyc08g066320.2.1) (File S2). The thermal profile consisted of 30 s at 95 °C, followed by 40 cycles of 5 s at 95 °C, 34 s at 58 °C, and 30 s at 72 °C. According to the mean ± and the standard deviation of three separate biological replicates, each gene’s expression indicates a value in relation to tomato *SlEF1b* (Solyc07g016150.2.1). A list of all the primers used is provided in Table S1.

### 3.3. Phloroglucinol Lignin Staining

As previously shown by Yi, et al. [49], lignin was stained with phloroglucinol on the flower’s pedicel abscission zones. Thirteen weeks following germination, the young flower stage of four abscission variants was harvested from the secondary peduncles of the primary inflorescences. To make the staining process and additional image acquisition easier, the preserved tissues of j, fij, fij like, and NJ flower pedicels were cut into transverse sections. Two volumes of 10% (*w*/*v*) phloroglucinol in 95% (*v*/*v*) ethanol were mixed with one volume of concentrated HCl to prepare the phloroglucinol-HCl reagent. Freshly sliced tissues were added to a 5 mL tube along with 3 mL of Ph-HCl solution while being shielded from light. To make sure that every part was stained, the tube was gently shaken and documented with a digital camera (D3200; Nikon, Tokyo, Japan).

### 3.4. Analysis of Cisregulatory Elements and Construction of Phylogeny

The promoter regions of the SlIDA and SlHAE genes were retrieved at 1000 base pairs upstream of the start codon in order to analyze the ciselements of these promoters. For the cisregulatory element analysis, the PlantCARE database was used and the resultant data were subjected to TBtools for visualization [50]. Heatmap clustering for cisregulatory of *SlIDA* genes was generated with the Euclidean distance measurement and complete cluster method. The sequences of *AtIDA* and *AtIDL* genes were used as described previously [51,52]. The EPIP motif of AtIDA was then used to identify the IDA like genes conserved in *S. lycopersicum*. The phylogeny was generated using SlIDA genes (see above), AtIDA (At1g68765), AtIDL1 (At3g25655), AtIDL2 (At5g64667), AtIDL3 (At5g09805), AtIDL4 (At3g18715), AtIDL5 (At1g76952), AtIDL6 (At5g05300), AtIDL7 (At3g10930), and AtIDL8 (At5g02591). The sequence alignments were performed through the multiple alignment program (MAFFT version 7, https://mafft.cbrc.jp/alignment/server/ (accessed on 11 October 2022)), and the phylogenetic trees were created using phylo.io [53].

### 3.5. Statistical Analysis

The results of qPCR included at least three replicates, and the data were presented as means with standard deviation (SD) for all the data sets. Using Microsoft Excel (2016), mean and SD values were determined for each treatment. The error bars in the figures show the SD.

## 4. Conclusions

In our prior study, we identified and examined the expression for five SlIDA genes (SlIDA, SlIDL1, SlIDL2, SlIDL3, and SlIDL4) in an ethylene-treated leaf abscission system [12]. In a more recent transcriptome study for the auxin-source depleted tomato flower abscission system, we identified three more SlIDL genes (SlIDL5, SlIDL6, and SlIDL7) [7], from which SlIDA, SlIDL6, and SlIDL7 were flower AZ-specific (Figure S2). The current study expands upon these results by analyzing gene expression for eight SlIDA genes and eight SlHAE genes in the natural development of flower abscission using four tomato abscission variants. Further, we examined the landscape of transcriptional regulation that may underlie the tomato IDA/HAE module. We show that different *SlIDA* genes are likely to be under the distinct transcriptional control either of developmental (SlIDA, SlIDAL2, SlIDL3, SlIDL4, and SlIDL5) or stress conditions (SlIDA, SlIDL6, and SlIDL7). The differing data appear to result from the difference between gene sequences and promoter sequences of eight *SlIDA* genes that were subjected to the respective analysis (i.e., gene body sequences for qPCR analysis and promoter sequences for cisregulatory elements analysis). In other words, the sequence similarity of SlIDA genes (SlIDA, SlIDL2, SlIDL3, SlIDL4, and SlIDL5) (Figure S3) was largely reflected in the analysis of gene expression (Figure 4), while the transcriptional landscape in the promoter sequence of other SlIDA genes (SlIDA, SlIDL6, and SlIDL7) was discernible in gene expression analysis from the auxin-depleted stress condition (Figure 5 and Figure S2). It appears that our previous results associated with the auxin-depleted flower abscission system may have caused stress responses, which in turn enhanced the AZ-specific expression for a set of SlIDA genes (SlIDA, SlIDL6, and SlIDL7) [7]. Similar analyses for SlHAE genes demonstrate that potential receptors for SlIDA genes may be associated with a broader context of transcriptional control by perceiving both developmental and stress signals that affect tomato flower abscission. Our findings provide future transcriptional regulatory targets for functional validations to gain precise biological insights into the potential bimodal role in the regulation of development and stress responses by the tomato IDA/HAE module.

## Data Availability

Not applicable.

## Supplementary Materials

The following supporting information can be downloaded at: https://www.mdpi.com/article/10.3390/plants12010185/s1, File S1: Diagram for natural abscission system, flower removal expression for SlIDA, and the relationship of AtIDA-SlIDA; File S2: Information about tomato *IDA*-*HAE* genes; Table S1: Primers used in the study. Reference [54] are cited in the supplementary materials.
