# Supplementary material for: Involvement of IDA-HAE Module in Natural Development of Tomato Flower Abscission
Source: Plants (Basel). 2023 Jan 1;12(1):185. doi: 10.3390/plants12010185 (PMC9823658; doi:10.3390/plants12010185)
Supplement: Supplementary file 1 [file plants-12-00185-s001.zip › Supplementary File 1. Diagram for natural abscission system, flower removal expression for SlIDA, and relationship of AtIDA-SlIDA_11-28-2022.pdf]

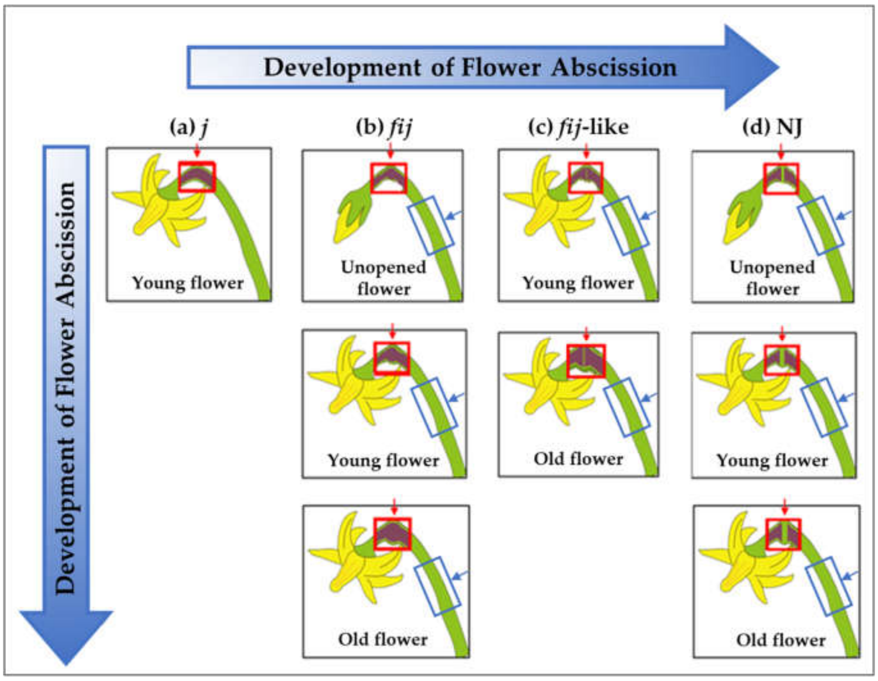

Figure S1. Schematic diagram of development of flower abscission and abscission zones (AZs) in four natural tomato abscission variants. (a) *jointless* (*j*), (b) *functionally impaired jointless* (*fij*), (c) *functionally impaired jointless* like (*fij*-like), and (d) normal joint (NJ). The samples of of *j* (midpoint of pedicels), and three developmental stages of the AZ and NAZ from *fij*, two developmental stages of the AZ and NAZ from *fij*-like, and three developmental stages of the AZ and NAZ from NA variant were used in this study. For phloroglucinol staining, the young flower stage of each abscission variant was subjected to the analysis. The AZ and NAZ samples for various developmental stages above were collected from two neighboring peduncles to further enrich for RNA extraction.

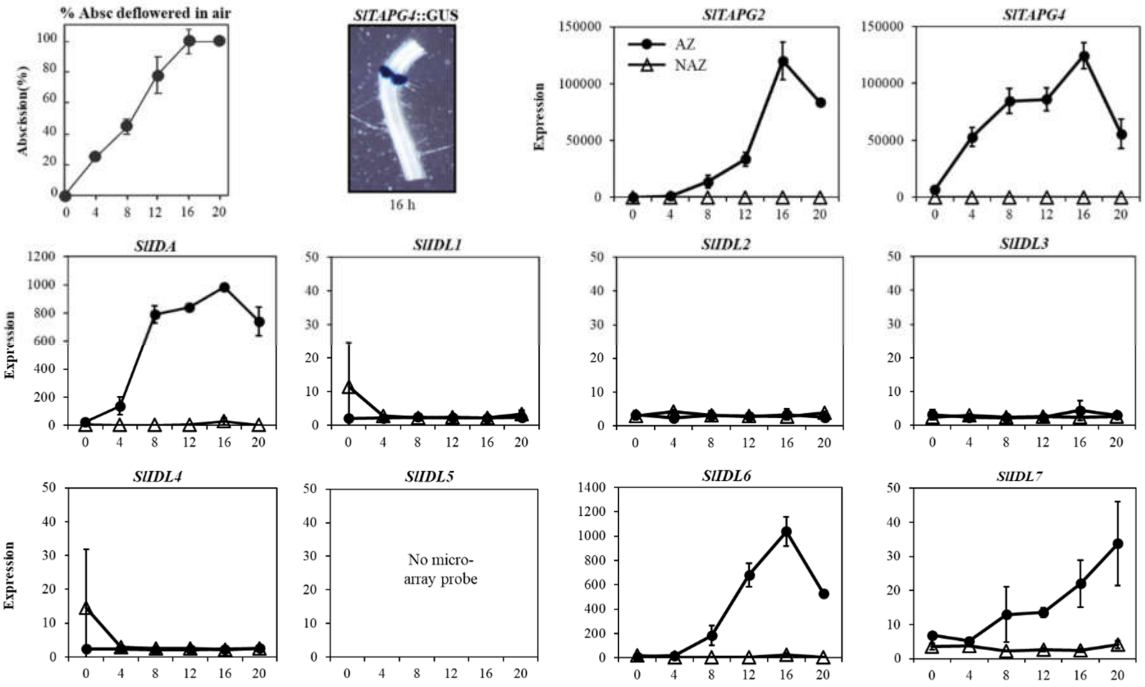

Figure S2. The flower AZ-specific expression for *SlTAPG* and *SlIDA* genes. Total RNA of flower AZ (•) and NAZ (proximal side of pedicels, Δ) extracted from 0, 4, 8, 12, 16, and 20 h after removal of flower, which is auxin source that inhibits abscission, is used for the analysis. By the 12 h and 16 h after flower removal 80 percent and 100 percent of flowers were abscised, respectively. Gene expression analysis for tomato flower abscission in the deflowered system has identified three AZ-specific (transcript of AZ compared to that of NAZ) *SlIDA* genes (*SlIDA*, *SlIDL7*, and *SlIDL8*). The data shown here are adapted and replotted from Hong, et al. [1], Meir, et al. [2], and Kim, et al. [3]. Note that there was no probe for *SlIDL5* on the microarray chip.

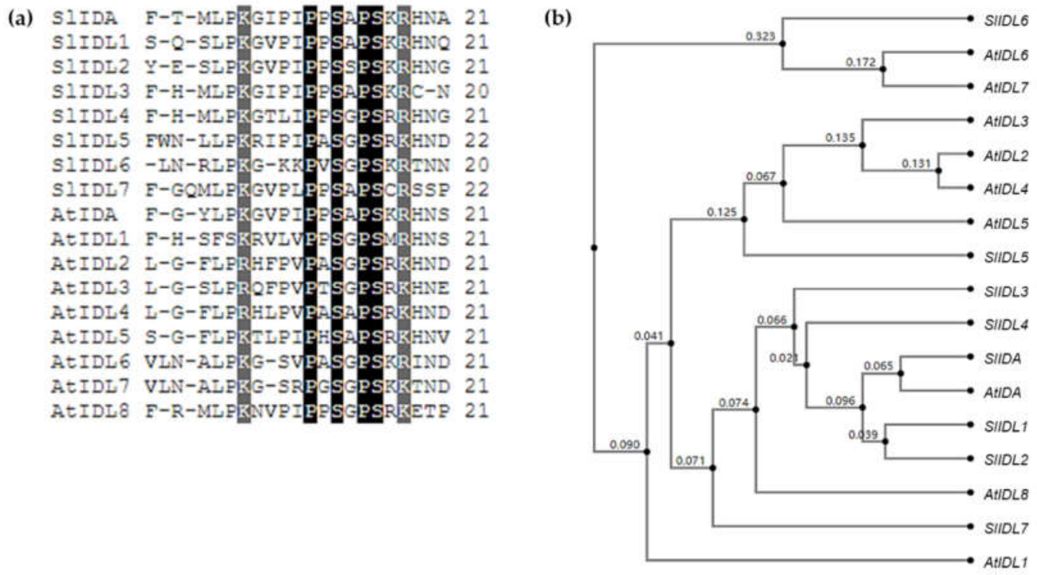

Figure S3. The phylogenetic tree of IDA-like genes conserved in *Arabidopsis thaliana* and *S. lycopersicum*. (a) The sequence of EPIP domain from Arabidopsis IDA genes and tomato IDA genes were aligned for the comparison. The alignment was performed using Multiple alignment program, MAFFT version 7 (https://mafft.cbrc.jp/alignment/server/, accessed on 11 Oct. 2022). (b) The phylogenetic trees were visualized with the resultant alignment and using the default setting of phylo. lo.

References

1. Hong, S.-B.; Sexton, R.; Tucker, M. L., Analysis of gene promoters for two tomato polygalacturonases expressed in abscission zones and the stigma. *Plant Physiology* **2000,** 123, (3), 869-882.
2. Meir, S.; Philosoph-Hadas, S.; Sundaresan, S.; Selvaraj, K. V.; Burd, S.; Ophir, R.; Kochanek, B.; Reid, M. S.; Jiang, C.-Z.; Lers, A., Microarray analysis of the abscission-related transcriptome in the tomato flower abscission zone in response to auxin depletion. *Plant Physiology* **2010,** 154, (4), 1929-1956.
3. Kim, J.; Sundaresan, S.; Philosoph-Hadas, S.; Yang, R.; Meir, S.; Tucker, M. L., Examination of the abscission-associated transcriptomes for soybean, tomato, and Arabidopsis highlights the conserved biosynthesis of an extensible extracellular matrix and boundary layer. *Frontiers in plant science* **2015,** 6, 1109.
