# Supplementary material for: Involvement of IDA-HAE Module in Natural Development of Tomato Flower Abscission
Source: Plants (Basel). 2023 Jan 1;12(1):185. doi: 10.3390/plants12010185 (PMC9823658; doi:10.3390/plants12010185)
Supplement: Supplementary file 1 [file plants-12-00185-s001.zip › Supplementary File 2. Information on SlIDA-SlHAE genes_11-28-2022.pdf]

Tomato Gene IDs used in this study

Tomato *SlEF1b* gene information
>SlEF1b-1: Solyc07g016150.2.1

Tomato *SlTAPG* gene information
>SlTAPG2: Solyc02g067640.2.1
>SlTAPG4: Solyc12g096750.1.1

Tomato *SlIDA* gene information
>SlIDA: Solyc05g010000.1.1
>SlIDL1: CP023762.1
>SlIDL2: CP023760.1
>SlIDL3: Solyc07g044890.1.1
>SlIDL4: Solyc05g007040
>SlIDL5: XM_010314166
>SlIDL6: Solyc06g050140.1.1
>SlIDL7: Solyc09g005780.1.1

Tomato *SlHSL* gene information
>SlHSL : Solyc02g077630.2.1
>SlHSL1: Solyc03g006300.1.1
>SlHSL2: Solyc07g053600.2.1
>SlHSL3: Solyc03g006300.1.1
>SlHSL4: Solyc04g077010.2.1
>SlHSL5: Solyc02g091860.2.1
>SlHSL6: Solyc08g066270.1.1
>SlHSL7: Solyc08g066320.2.1

SlIDA Solyc05g010000.1.1
Protein Sequence:
>Solyc05g010000.1.1
MAFSFSSSKTLYLSSKLTCLILVISLLFNYGHIVEASRFGRIMMVEENSRIFSSQHMKVYKKENAYKVD
NLLFTMLPKGIPIPPSAPSKRHNAIEDSTPQN
CDS Sequence:
>Solyc05g010000.1.1
ATGGCTTTCTCTTTTTCTTCTTCAAAAACCCTTTATTTATCAAGCAAATTAACTTGTTTGATACTT
GTTATTTCTCTACTTTTTAATTATGGTCATATTGTTGAAGCATCAAGATTTGGGAGAATTATGATG
GTAGAGGAAAATTCAAGAATATTTTCATCACAACATATGAAGGTATACAAAAAAGAAAATGC
ATACAAAGTTGATAATTTATTATTTACTATGTTACCAAAAGGGATTCCAATTCCTCCTTCTGCTCC
ATCAAAGAGACATAATGCTATTGAAGACTCTACACCTCAAAATTGA
Gene Sequence:
>Solyc05g010000.1.1

ATGGCTTTCTCTTTTTCTTCTTCAAAAACCCTTTATTTATCAAGCAAATTAACTTGTTTGATACTT
GTTATTTCTCTACTTTTTAATTATGGTCATATTGTTGAAGCATCAAGATTTGGGAGAATTATGATG
GTAGAGGAAAATTCAAGAATATTTTCATCACAACATATGAAGGTATACAAAAAAGAAAATGC
ATACAAAGTTGATAATTTATTATTTACTATGTTACCAAAAGGGATTCCAATTCCTCCTTCTGCTCC
ATCAAAGAGACATAATGCTATTGAAGACTCTACACCTCAAAATTGA

5,-Upstream Sequence (1kb):

>Solyc05g010000.1.1
TGTGTATAATGAAACAATTAATTATCTTGATTAGGCCATTAATTATTGATCATATTAGGTATTAAC
AATAAAGTGTTCATAACAACAAGCATATATTTTAGGTTGAAGTACTTTTCAATTTTTAACAAGA
GGTTTCGCGTTTGACCTCGATATATGAGTTATTTTTTAAAAGAAAGTGCTATACTTTGCGCATAA
GTTGAAATTTTTCGATGAAAATTTAACTGAATCAGACTATGAAGCAAGTATTGCACGTCTAAGG
TGAGGCTGTTATTTTTTTTTTACTTATTTAGTGTCTGAAAGTATTAGGGTTCGACTAAATTGTTGTT
ATTATGATTTTTCATTTGATGTTTGATATGATCTATATTGAAGCTTGATTAAATTCATATACGTGTA
TTGCAAGATCATGGAAAAGATGCTTTCAACAAAGGTTTCTCAATTTTCAGAAGCTAGAATGTG
AGACGCCTAATTAAAATTGAATGATACCAACCATCCCACCATATTACTCTTTTAATTAATCTAAA
TATTTTTTTATTTTTCTGTTACTGACTTGGAATTTTTGTTAACAAACTAATATCCAAATTGTAATAT
TAAATTTTTGCAGACCCCAATGTATGTACTTCAACATCAATTTCACCTAACACAATTAAACATA
GTATTCTCCATGTTAGTTCAAAACTTTTTTTTTCTTTTTACTTATTATTGGGCAAGAATTCAAAGT
GAAATTTTCAAATATTTGACAAATTCTAAGTACACCATGTGGCATCATATATATTAATTATAACT
ACTAATTAATGCTTGCAAATTCTAAATTCCTCTATTTTTTCAAACCAAACCACACAGAGACAAA
TTTCTAACGTAAGGGAAACTCATGAGAAAATCCCTTTCATTGACTTGAAAAAGAAGTGAAAA
AAAAACAATATTTAAACAAGAGATATAACTAATCTACCTACATTCACTCGATGTTTACATCAAA
TTATATCAGTTTATCACGATTAATGAAT

SlIDL1 (CP023762.1)

Protein Sequence:

MLKKNHNTTLLIYLLLVILVVDHHDHHVNAVKNSQVVNVKPLLPSNNNSKSSFSQSLPKGVPIPPS
APSKRHNQINI

>SlIDA2 ORF
ATGTTGAAAAAAAATCACAACACAACACTATTAATTTATTTACTTCTTGTGATTTTGGTGGTTGA
TCATCATGATCACCATGTTAATGCAGTAAAAAACTCACAAGTTGTTAATGTTAAGCCCTTATTA
CCTAGTAATAACAATTCTAAATCATCTTTTTCTCAATCTTTGCCAAAAGGAGTCCCTATTCCACC
TTCTGCTCCTTCCAAAAGGCACAATCAAATCAATATATGA

5,-Upstream Sequence (1kb):

ACATAAATAATTGCTTCAAACATAAATAATTGGTAAGGACTAAGAGGCAACTTGGACAGTACC
ATTCATTTTACCACAACTAAAAAACAAATAAACCGTTAATTAATGGGAAAATTGTATATAATAG
CAAATTAATGACCTAAAATAAATGGAATAGCTAGGGTTTAATTTAATTGCGCTTCATAGCAAAC
ATTAGCTAAAATTTGCCAGCGTCTCTCTCCAAAAATCTCGCTCGTCACTCTCCATTCTCGCTCG
CCTCTCTCGCTTTATACACAGAAATGTATAATTCTGTTTCTATTTTGTATAAAGCGAGAGAAAAT
TGTGTATACACATGCAAAAATATATATCATCGTGTTATACACTTAATTATACAATTTACAAACAC
TTCACTTCAAATATTGCAGAGAAAAAGGCCAACGAATTATACAATTGCGAATTATACAATTGC
AGTGAAATACAATTTTCTCTAGCTTTATACAACAGAAGTGTATATATTGTGTTTCTGTTTTTGTAT
AAAGTGAGAAAAATATATATCTTCTTGCTATACACTTATAATTATGAAATATACATACATTTTAAT
TCGATTCAAATGTATGCAAAGCAAATTATACAATTGTAGCGAAATAGGCCAGCAAATTATACA
ATTTAGGTCAGCGAATTATACAATTGTATATGTATAGCGAATTATACAGTTTTATGTTTGCTATGG

ATCGCAATTATGCAAACTTTGCTATAGCATACAAATATGAATTTTTTGTTTGCTATATGTGAAAG
TTGCCCTTAATTAATTATATGAAACCATTTATAATAAGCACAAAACTTATACTAGTGTTTGAACA
AAGTGAAAATGAATAACAAATGTAGTATATATGTTAGCATAGAGTTACTTTTTCATTGTCTACTT
GTAGAGGCGGATCACAATACATATAGTTGTATGTTACATGGATTCAACCTTAAAAGTTGTAACA
TTAAGCTCGTTACATTTTAAAATTATAAATTC

SlIDL2 (CP023760.1)

Protein Sequence:

MEKMSIKNTTTISIIFVLVIIIQHAHGASHTQFFKVKPLPISNKNNKSPYYESLPKGVPIPPSSPSKRHN
GINLKRYWP

>SlIDA3 ORF

ATGGAAAAAATGAGCATAAAAAACACAACTACAATATCAATCATTTTTGTCCTTGTGATAATAA
TTCAACATGCTCATGGTGCAAGTCACACACAATTTTTCAAGGTGAAGCCTTTGCCAATTAGTAA
TAAAAACAATAAATCTCCTTATTATGAGTCTTTGCCAAAAGGAGTCCCAATTCCACCTTCTTCT
CCTTCAAAAAGACACAATGGAATCAACCTCAAAAGGTATTGGCCATGA

5,-Upstream Sequence (1kb):

GGTTAATCTTTTGAAGATGAAGTGCATATACATGATACCTTAAACGAATACCACAATAGAATAG
AGGAGAAAAGTAAAGAGCTTCAAAACGCACATATTCAAGATAATATGCGCGTTTTGGAACCTA
ATTGTGACTAAAAGTCTATTACTTGGTTTCCCATCAGGTATTCGAAGCCCTGTAGCACATATTGG
AGTTTCAATTAAATTTAAATCGCATACTGGAGAGCTCATTTTGAGGTGACATTCCCAATATAATT
TTTTGCATACCTAGGACGCGAACCTGAAATCTCTGGTTAAGGATGAAACAACTCTATCACTAC
ACGACAACCCATATTGATGCACATAGATAATTAACTAAAGTATTCAACAATATATTGACCTCCC
AATATATATATAACATACTAGAGGACGATTCATAACCTTTAAGAAGAAACCTACCATCATTCAA
CAAATAAAGTGCATTTTTGTTGTGTGTTTGTAACATGCTATATGATGAATAAATTGATAGAAATA
ATTAGTGAAGATTTGTCTCATTTGTGATACCAAATGCTGCTAGTCCAAGAAAACATGTCCTTCA
TTTATTGCTTCTTCTTTTCAATATCATCTCTTGCATTCAATGTTTTCATTTCCCTCTCTTCTTCCATT
CTTTGGTCATTAAATGGGGTGGACCATTACATTGCACCTAAAGCAGACTTTTGGGGATGGGGG
GTTGGTGTTCAAAGTTCTCATATCTTTGTACAATAATATCGTTATTCACTATATACTAATAATATTT
GTATTATTTAAAAAAATTAAGAAATAATTTATATTTAACATATTTTTCTAAATATTAAATTTAATA
AAATAGAAAGATAAAATAGTAATATTATCTCTATTATTTATTATTTCTTAAAGAGTATGTCAAATC
AATAATAAATAATTATTGTTGAACAGAGAGTATTAATTTTTTTTAAAAAGCAAACCATGAAGAA
TTTGAATGTATATATTTGTAACTTTATAA

SlIDL3 (Solyc07g044890.1.1)

Protein Sequence:

>Solyc07g044890.1.1
MAYSANSKTLHYISSWKFICLILTLSLVLDHGHGTTCPPTPSRMPRRLKEEASRMFSELSDEKKEFLSS
TSNRFHMLPKGIPIPPSAPSKRCN

CDS Sequence:

>Solyc07g044890.1.1
ATGGCTTATTCTGCTAATTCCAAAACCCTTCATTATATTTCCTCATGGAAATTCATATGCTTGATT
CTCACACTATCTCTTGTTCTTGACCATGGACATGGTACTACATGCCCACCTACGCCGTCACGTAT
GCCGAGGCGTTTGAAAGAGGAAGCTTCTAGAATGTTCTCAGAACTTTCTGATGAAAAGAAAG
AGTTCCTCAGCAGTACTAGTAACCGGTTCCATATGCTACCAAAAGGGATTCCTATTCCTCCTTCT
GCACCATCAAAAAGGTGCAATTAA

Gene Sequence:

>Solyc07g044890.1.1
ATGGCTTATTCTGCTAATTCCAAAACCCTTCATTATATTTCCTCATGGAAATTCATATGCTTGATT
CTCACACTATCTCTTGTTCTTGACCATGGACATGGTACTACATGCCCACCTACGCCGTCACGTAT
GCCGAGGCGTTTGAAAGAGGAAGCTTCTAGAATGTTCTCAGAACTTTCTGATGAAAAGAAAG
AGTTCCTCAGCAGTACTAGTAACCGGTTCCATATGCTACCAAAAGGGATTCCTATTCCTCCTTCT
GCACCATCAAAAAGGTGCAATTAA

5,-Upstream Sequence (1kb):

>Solyc07g044890.1.1
ATTAACGTGGAAAAACTACCACGTCTTCCTCCTTATCCTTAGGGAAAACCATCGTATACCTTGG
CCAATGATCATGACGACTCCTTGAGAAAGAAAAGTAGTCTTTCAAGACTCTTGTAAGATCTTC
GAAGGAGAAAGTTTGCGGAGCCGTATGCACTGCCGCATCCACCCGTACATCATGGTACAGGTA
CCAGTTCTTGTTCTCTATCACACTCTTAGTTCGTATACTTAAAGGTACTCCTTTATATTACTTCCC
AAAACCTTAATCGTCTTATTCGGTAAAGAACTTTTAAAAGATTATCCCAAATACAAAGAGAATA
AACTTCTTATCAACTATCCCTAAATGCATTCCCATATATATCCGTACCTCTTTCACGTTTTAAGTT
GTTTACCTCTTAAAAATCATAGCTAAAAATAGAAAATTCAAATTTAAGATTTAAATTTGAGCTC
TAGTTCCCTCTCACCTAGTATACATTCCCCAACAATTTAATCCAGAATCTGGATTGAGTGTGGA
GTTTTCGATCAAGTTTGCCTACTCTTAATATTCCTCAGGTGGGTAGAGTAATTAGTAGCTACGCC
CTGAAAACAGTTAAAAATTGTGGAGTGGAGTGTAGGCCACGAATTGTAGATTACGCACTCGTT
AAAACTAAAAACTCCCGGGGTTGTAACCCAATCTGCCTGAGAAGAGACTATGGTACACTTTAA
TCCAGAACCAGGATTTATATGGTACAATTTAATCCAGAATCTGGATTGAGTGTGAGTTTTCGAT
CGAGTTTACCTCCTCCTAACAAGTTAGGAATACTCCTAAAGTGGGTAGACTAATTAGTAGCTAC
ACTCTGAAAACAGTAAGAAATTGTGGGGTGGAGTGCGGGTCACGAATTGTAGACCACGCACT
CGTTAAAACTAAAAACTCCCGGAGTTGTAGCCCAATCTGCCTGAGATGAGATATGGTACACTT
TAATTCAGAACCAAGATTGATATGGTACAATTTAATCCAGAA

SlIDA4 (Solyc05g007040)

Protein Sequence:

>Solyc05g007040.1.1
MVQKEELESLLVAYETNYSFETLSFHFHRNISEMILKIVYKRLHILEMVLKIVYKRLHLLNSSSTIFSLK
KHSQEQEIM

CDS Sequence:

>Solyc05g007040.1.1
ATGGTCCAGAAGGAGGAATTAGAGTCCCTTTTGGTAGCATATGAAACAAATTACTCTTTTGAA
ACTCTTTCTTTTCATTTTCATCGTAATATCTCGGAGATGATTCTGAAAATAGTGTATAAGCGTCTT
CATATTTTGGAGATGGTTCTGAAAATAGTGTATAAGCGTCTTCATCTTTTGAATTCATCGTCGAC
GATATTCTCACTGAAGAAGCATAGCCAAGAACAAGAGATAATGTGA

Gene Sequence:

>Solyc05g007040.1.1
ATGGTCCAGAAGGAGGAATTAGAGTCCCTTTTGGTAGCATATGAAACAAATTACTCTTTTGAA
ACTCTTTCTTTTCATTTTCATCGTAATATCTCGGAGATGATTCTGAAAATAGTGTATAAGCGTCTT
CATATTTTGGAGATGGTTCTGAAAATAGTGTATAAGCGTCTTCATCTTTTGAATTCATCGTCGAC
GATATTCTCACTGAAGAAGCATAGCCAAGAACAAGAGATAATGTGA

5,-Upstream Sequence (1kb):

>Solyc05g007040.1.1

GTGTAATAGAGAACAAGAACCGATACGAAGAAGTCACTCTTATAGCAGCTGCTACTTAAGTTT
TCTACTTCTGCGAATATGTGATAAAAGTCTTGGTAGAGGTTTTATACTTCTGCGAATATGTGATA
AAAGTCTTAGTAGAGGCTCTATAATGCTACTTTTACTTTTCTTTCTCAAAGTTTTCTCATTAAACA
AAGTATACGATGGTTTTCCCTGAGATTAAGGAGGAAGACCTGGTAGTGCATCTGTGTTACCTCT
ACCACTGAATAGATTAATAGGAATTTTCAATTAATTAACGAATTATTATTTCTTATTAATACAATA
TTAGTATATATCAAAGTAAACCTATATTATAAAGTATTGTACTATACATTGTTATGATAATAATATC
TGACTAACTTCTCGGATAGACCAAATTAATATATATCAATAATATTTTTAATTCTTCCAATAATTA
GAACAATAAATGTTAATACAACTCAAATATACGTATATATATATATATATATATATATATATATATA
TATATACTTTTAAGTTTAATTATACTTTCTTGATACTGTCATGCATATGAAACAGCATATCACGGT
AGGAGGGGCGATCGAAATACCCGCCTTATAACAGGGAGCTTAATAACGTACACAAGAAAACT
CGATACTTAATTTCCTTATTTACATCAATTTTAAAAAGCGTACTTTTGTGATTATATAAAAAAAA
GAAAACAGTGATTAAGTTAGTTTTATAAACACGGATTTGTAAAAAGCTACTTTTTAAAGTTACC
CTTTTTATTACTAGAACATCATTACTTGATTACCGAGTAGATCAATCAATCGTGGTCTCACCTCG
ATCGATCGGTCTATCCTGATTCCCCCCAAGTTGCTATTTTTTAAATGTACTATATACTAATTAAAA
AAAATTATATACATATATCTCATTTACAACACATGATCAGAGACATAAAGAAACAAAAGTAGA
AAATGCATCGATGTTGTAAAACTAT

SlIDL5 XM_010314166

AGAGTGAAACTTGAAAGAATAAAATATACGTAGATTATAACTTTGAATCATTTCTTCTTTCCTTT
TTCCATTTCTTTTTCCTTTTTATTAATCTTTTTCCCCCTTTATTCAATTACCTATCAAGTCATTACTC
TCTATAACCCAAACCAAATGGCCCCCTTTCAAAAAGCCTTATCAAACTTTTCAAAACTTTCTAC
TTCATATGTCTTCATTCTTCTTCTTCTTCTTTCTAACCTTATTTGCCAATAGATTTACATATGTTTTC
TTCTTTTTCTAAATTAGCATAGTGATTTAAGTTTCAAGAATGATAAGCTTTTTCAGAAGAAAAAT
ACTTGTCTTATGGATGGCTATTATATTAATCTCTATTTTTGGCCATTTTTGTCATGGTTCAAGAAG
CAACTCACAAGTATTCAATACAATAAATAACCAAAGAAACTCTTACAATCATGGCCATTTTTGG
AACTTATTGCCAAAAAGAATCCCAATTCCAGCTTCTGGTCCATCAAGAAAACATAATGACATT
GGTTTAAAGAGTACTTGGAGATTACCATGAAGATTATTTCAAGAAATTAAATATTGCCAACTTC
TTTTTTATTTTTTTGGGTACTACTAATCCTTCTTCTTCTTCTTCTTCTTCTTAATTTCTTGGATGAAC
ACATGTACATTTTTTTGGGTAATTTTAAGATCTTTTTTTTTGTTAATTGACTTAAGGGTATTTTGTG
TTAGGTTGGTTTTTTTTTTTGGTCATGGTGACTAGAAGGGATATTAAATGAAAAGGGGAGAAAA
AATTAAGAACATTTTTTTGGGTTTCTTGGATCAAAATATAATTGATCATCATCTAGCTAGCTTAG
GGGTGGTTGGGGTGGGGGTATTTTGGTCTTTATTAATTAGTATGCTTTGAAAAGCTTTGTGTATG
TAATTTATGTATACATAGAAGAGATCATGATAATACAAGATGAAATGACTTTATTTCAAGTTTTA
TTTGAA

SlIDL6 Solyc06g050140.1.1

Protein Sequence:

>Solyc06g050140.1.1
MADSKLCYKVSQITVTFLVVFLFISQRSCARPFNIVLVTGKDSNSWSDEKYFDEDKMNVRRERVNML
LNRLPKGKKPVSGPSKRTNNLKD

CDS Sequence:

>Solyc06g050140.1.1
ATGGCGGATTCTAAATTGTGTTACAAGGTTAGCCAAATCACAGTGACCTTCCTCGTGGTTTTCC
TCTTTATATCTCAACGTTCATGTGCTAGACCGTTTAATATTGTACTTGTGACTGGCAAGGACTCA
AATTCATGGTCCGATGAGAAATATTTTGATGAAGATAAAATGAACGTGAGAAGGGAACGTGTG

AATATGTTATTAAACCGGCTTCCTAAAGGGAAGAAACCGGTGTCTGGGCCAAGTAAAAGAAC
AAACAACCTCAAAGACTAG

Gene Sequence:

>Solyc06g050140.1.1

ATGGCGGATTCTAAATTGTGTTACAAGGTTAGCCAAATCACAGTGACCTTCCTCGTGGTTTTCC
TCTTTATATCTCAACGTTCATGTGCTAGACCGTTTAATATTGTACTTGTGACTGGCAAGGACTCA
AATTCATGGTCCGATGAGAAATATTTTGATGAAGATAAAATGAACGTGAGAAGGGAACGTGTG
AATATGTTATTAAACCGGCTTCCTAAAGGGAAGAAACCGGTGTCTGGGCCAAGTAAAAGAAC
AAACAACCTCAAAGACTAG

5,-Upstream Sequence (1kb):

>Solyc06g050140.1.1

ATCAGAAACTCCAACAAACAAGAAAATGAACCGGGTCTGTGGCCAAAGAAGGGAAATCCTT
CGGCCAAATTATTGTATAAGTGTGCAAGGGAAGAGTGCAAGTAAAATAGAAGTAGTTTTATAA
AGAGTAGCCTGGTACTTAAACTCAGGAACGGTCAGTGTTCATGTTATAATTTGCCAGATCGTGT
ACTTGCAACTCTATATTTCTCCTTTTGGTGCTCCTTCCAGTGACACTAAACCGATTGGAACATTG
TGTTAAATCTTAGGCGGTACTTACTAACTAAGGATTAGTTTGAGCTCCCCTTTTACTCTTTACTCT
TAAATTAAATCTCTAAAACCGAATTCTCCATTCGATATATCTGTATGTTTTGTTCAACACGTATAA
TCTTTTTCAGTCGGATAATTAAAGTGTTCAGCATGCATTGCTGCATTGTAAATATCTTTTTATTTT
TTCTTTAAAACCACGATTCAGATGTGCGAAAGTGTAATATAACGTGTGCGATAATTCTACCGTC
GTATATAGTGTTTCAACCAATTCAAACTAATAATATATCGCACGACGAAATTAAGGTGATAGGT
TGAATAAGGAGTTTAACTGATTGAACTTACCAAATTAATTATTTCTTATAAAAATATGAAAATTA
TATTGTAAAATTAATACCCGCCTAGATCCTATAAACGAGTTTCAAGGAGCTAATGTTGGACTAT
AAAATACACACAATTAATTTTTTTTTTCGTATTATATATTTATACTATGAATTAAATTGTAATTAAC
TGTTAATACTAGGAAGTTAAATCTACACGTGTTTGCCTATGCATTTAAACATATTTTAACTTGTTT
AACTGTGTAAGTAGGATGTACTGTGGGATGTACCTTTAAAACACACGATGCACATAGTACAGT
ACATCCTATGTATATAGATGAACAAGTTAAAATATGTTTAATTTCACAGATACATAGGTGTAAGT
TTCAATTCTTAGTATTAATAGTTAGTTATTT

SlIDL7 (Solyc09g005780.1.1)

Protein Sequence:

>Solyc09g005780.1.1

MMNEKKKFFKSLLFLFLTTLYYSSSYAITNRKILDLKSEIEIKTSSSVFGQMLPKGVPLPPSAPSCRSSPG
TPPSCPMAQPEIVDVVESFTP

CDS Sequence:

>Solyc09g005780.1.1

ATGATGAACGAAAAAAAGAAATTTTTCAAGAGCCTTCTTTTTCTGTTTTTAACAACCCTTTACT
ATTCTTCAAGTTATGCAATTACGAATCGAAAGATATTGGATTTGAAGTCCGAAATTGAAATTAA
AACATCGTCAAGTGTTTTTGGTCAAATGTTACCAAAGGGTGTACCATTACCTCCATCTGCTCCA
TCTTGTAGATCAAGTCCTGGCACACCTCCTTCATGTCCCATGGCACAACCAGAGATCGTAGAC
GTTGTTGAGAGTTTCACCCCCTAA

Gene Sequence:

>Solyc09g005780.1.1

ATGATGAACGAAAAAAAGAAATTTTTCAAGAGCCTTCTTTTTCTGTTTTTAACAACCCTTTACT
ATTCTTCAAGTTATGCAATTACGAATCGAAAGATATTGGATTTGAAGTCCGAAATTGAAATTAA
AACATCGTCAAGTGTTTTTGGTCAAATGTTACCAAAGGGTGTACCATTACCTCCATCTGCTCCA

TCTTGTAGATCAAGTCCTGGCACACCTCCTTCATGTCCCATGGCACAACCAGAGATCGTAGAC
GTTGTTGAGAGTTTCACCCCCTAA

5,-Upstream Sequence (1kb):

>Solyc09g005780.1.1
ATCCCCCACTTTGAGAGTTGTTGCAGATGCTAGAGACCAACACGGTACCCTGTACTTCCTCCA
CACGGTCCTGAACTAGATGTTCTACCTCGTCTACCTCCATTACCATGTGGGAAACCATTGTAAA
CTGGTTTTTGTGAACTGCTACAAAATTAAAGTTAAAGCCTGAAGTTTAGGTTATAGAAAGCTAA
GCATTAACGTATTGAACTTCTTATCATTTCCCAACAATTTTTGTCTTTTTCTTCCGAGAACTTTTT
AAAGAAAAAAAGCAAGTAGTATAAAATTTCATCATAAGTCTTAACTTTATACACATGTTCTTAC
ACTATAAAATTAATACTTACTCGATACAACAAATATATCTCTTTTTTTTCACACATGGTAATAAC
TTTTAGAACAAAAATAATCTTTTTTATATATATAGTAATTTATTATTAATATAGATTTGAGGTCCTA
TTCTTACTTTACTTTTATGTATTCAAACTGAGCTGGCAATATAAAAAGGTTATTTATGTTCCTATA
CTTAACTCAGAAATAGGATTAAGGATTTCATTTTAAATTTATTCTCCAGAATACAAATAAAATTC
TTTTTTTCAGTAAATAAACAACACTAAAATAATTAGAATCAGGAATTTATCATAATTAGAAATA
AAGCTTTTTGAAAAAAAACTCTACTTAAAGTAAATTGATTATGTAAGAATGAGTAGTTTCATTG
TTGGTATACCGTAAAACATAGATTTTCTTGTAAGTTTTTTAGTTGAATTTATATTAAGATTAAATC
CCGTAATAATAAAAGTGTTAGAATATTAATTAATCGAATGTTGCAGAGATATCTATGTAGTATCT
TTAGTTATTTCATGTGCTTGAATTATTTTTTATTAACCCTACCTTTCTACCTTGTTAATTTTCATGTT
TATATTAATAAATGAAAAAATGATAATGTAGTTGGTATAATAAACTCAGTAGGAAGGTTCTAAA
TTTCGACTATAGTGATCAGATTTT

>SlHSL ORF Solyc02g077630.2.1

Protein Sequence:

>Solyc02g077630.2.1
MKSSISIMFLQILVTLFLPTLIFSLNQEGLYLHNVKLGFDDPDNVLSNWNEHDDTPCNWFGVSCDKF
TRSVTSLDLSNANVAGPFPTLLCRLKKLRYISLYNNSLNSTLLEDFSGCEAVEHLDLAQNFLVGTLP
ASLSELPNLKYLDLSGNNFTGDIPVSFGSFQQLEVLGLVGNLLDGSIPAFLGNVTTLKQLNLSYNPFT
TGRIPPELGNLTNLEVLWLSDCNLIGEVPDTLGRLKKIVDLDLAVNYLDGPIPSWLTELTSAEQIELY
NNSFTGEFPVNGWSKMTALRRIDVSMNRLTGTIPRELCELPLESLNLYENQMFGELPQDIANSPNLY
ELRLFHNRFNGSLPQHLGKNSPLLWIDVSENNFSGEIPENLCGKGLLEELLMINNLLSGEIPASLSEC
RSLLRVRLAHNQLSGDVPEGFWGLPHLSLLELMDNSLSGDIAKTIASASNLSALILSKNKFSGSIPEEI
GSLENLLDFVGNDNQFSGPLPASLVILGQLGRLDLHNNELTGKLPSGIHSLKKLNELNLANNDLSG
DIPMEIGSLSVLNYLDLSGNQFSGKIPLELQNLKLNQLNLSNNDLSGDIPPVYAKEMYKSSFLGNAG
LCGDIEGLCEGTAEGKTAGYVWLLRLLFTLAGMVFVIGVAWFYWKYKNFKEAKRAIDKSKWTLMS
FHKLGFNEYEILDALDEDNLIGSGSSGKVYKVVLSKGDTVAVKKILRSVKIVDDCSDIEKGSIQEDGF
EAEVETLGKIRHKNIVKLWCCCTTRDCKLLVYEYMPNGSLGDLLHSSKSGLLDWPMRYKIAMDAA
EGLSYLHHDCAPPIVHRDVKSNNILLDGEFGARVADFGVAKAVEANAKAIKSMSVIAGSCGYIAPE
YAYTLRVNEKSDIYSFGVVILELVTGKRPVDPEFGEKDLVKWVCSTLDQKGVDHVIDPKLDTCFKEE
ICKALNIGLLCTSPLPINRPSMRRVVKMLQEVGGGNLPKAASKDGKLTPYYYEEASDQGSVA

CDS Sequence:

>Solyc02g077630.2.1
ATGAAATCTTCAATTTCAATAATGTTTCTTCAAATCTTGGTTACCCTTTTTCTCCCAACTTTGATT
TTCTCACTTAATCAAGAAGGTCTTTATTTACATAATGTGAAGCTTGGATTTGATGACCCTGATAA
TGTTCTTTCAAACTGGAATGAACATGATGATACACCATGTAACTGGTTTGGTGTTTCATGTGAC

AAATTTACTCGCTCTGTTACTTCATTGGACCTTTCTAATGCGAATGTTGCTGGTCCTTTTCCCACT
CTGCTTTGTCGGTTGAAGAAGCTGCGTTACATTTCCTTGTATAACAACTCGCTTAACAGTACTCT
TCTTGAAGATTTTTCTGGGTGTGAAGCAGTAGAGCATCTTGATTTGGCGCAGAATTTCTTGGTG
GGTACACTTCCGGCGAGTTTATCTGAGCTCCCAAACTTGAAATATCTTGACTTGTCGGGTAATA
ACTTCACCGGAGATATTCCGGTGAGTTTTGGTTCTTTTCAGCAGCTTGAAGTTCTTGGGTTAGTT
GGGAACTTGCTTGACGGGAGTATACCGGCGTTTCTCGGGAACGTTACGACGTTGAAGCAGCTG
AATCTGTCGTACAACCCGTTTACTACTGGTCGGATCCCGCCGGAGCTGGGAAATCTGACGAAT
CTTGAGGTTTTGTGGCTTTCTGACTGTAATTTGATTGGGGAAGTTCCTGACACATTGGGGAGGT
TGAAGAAGATTGTGGATTTGGACCTTGCTGTTAACTACTTGGATGGGCCGATTCCGAGTTGGCT
CACTGAGCTGACTAGTGCTGAACAAATTGAGCTGTATAACAACTCGTTCACCGGCGAGTTTCC
GGTGAATGGGTGGTCGAAAATGACGGCGTTGAGGCGAATCGACGTTTCGATGAACCGGTTAA
CTGGTACGATTCCGAGGGAGTTGTGTGAGCTGCCACTTGAGTCACTCAATCTTTATGAAAACC
AGATGTTTGGTGAATTGCCACAAGACATTGCAAATTCACCAAACTTGTATGAGTTGCGCCTTTT
TCACAACCGTTTTAATGGGAGTTTACCTCAACATCTTGGAAAAAATTCACCTTTGTTGTGGATT
GATGTGTCGGAAAACAATTTTTCTGGTGAAATTCCGGAAAATTTATGTGGTAAAGGGTTGTTGG
AGGAGCTTTTGATGATAAATAACTTACTTTCTGGTGAAATTCCTGCCAGTTTGAGTGAATGCCG
GAGCTTACTGCGAGTGAGATTGGCTCATAACCAGTTATCCGGTGATGTTCCGGAGGGGTTCTG
GGGTCTGCCTCACCTTTCCCTGCTTGAGCTCATGGACAATTCACTCTCCGGAGATATCGCGAAA
ACTATAGCTAGTGCTTCAAATTTATCAGCTTTGATTTTGTCTAAGAACAAATTTTCAGGTTCCAT
TCCTGAGGAGATTGGTTCTCTGGAAAATCTTCTTGATTTTGTGGGCAATGATAACCAGTTTTCTG
GGCCTTTACCTGCAAGTCTGGTGATTCTTGGACAATTGGGGAGGCTGGATCTTCACAACAATG
AGTTAACTGGTAAGCTTCCGAGTGGGATTCATTCTTTGAAGAAATTGAATGAATTGAACTTGGC
AAACAATGATCTTTCTGGAGATATCCCCATGGAGATTGGGAGCTTGTCTGTTTTGAATTATCTTG
ATCTATCAGGGAACCAGTTTTCAGGGAAAATCCCACTGGAGTTGCAGAATTTGAAGCTCAATC
AGCTGAACTTGTCGAATAATGACCTTTCGGGTGATATCCCCCCTGTTTATGCAAAGGAAATGTA
TAAGAGTAGCTTTTTGGGTAATGCTGGTCTATGTGGAGACATTGAGGGCTTGTGTGAAGGAAC
AGCTGAAGGTAAAACTGCTGGTTATGTTTGGTTATTGAGGTTACTATTCACCCTTGCTGGAATG
GTGTTTGTTATTGGGGTTGCTTGGTTCTACTGGAAGTACAAGAATTTTAAGGAAGCTAAAAGGG
CTATTGATAAGTCTAAATGGACTTTAATGTCGTTTCATAAATTGGGTTTCAACGAGTATGAAATC
TTGGATGCTCTTGATGAGGATAACTTAATTGGCAGTGGCTCTTCTGGGAAGGTTTACAAGGTTG
TTCTGAGCAAGGGTGACACTGTTGCGGTGAAGAAGATATTGAGAAGTGTGAAAATAGTAGAT
GATTGTAGTGATATCGAGAAAGGTAGCATTCAAGAAGATGGATTTGAAGCGGAGGTTGAGAC
GTTGGGGAAGATTCGACACAAGAACATTGTTAAGCTATGGTGTTGTTGTACAACTAGGGATTG
CAAACTTCTGGTCTATGAGTATATGCCTAATGGAAGTTTGGGTGATTTGCTACACAGCAGCAAA
AGTGGCCTTCTAGACTGGCCTATGAGATATAAGATAGCCATGGATGCAGCTGAGGGACTCTCC
TACTTGCATCATGACTGTGCTCCACCTATCGTTCACAGAGATGTTAAGTCAAATAACATCTTGC
TGGATGGTGAGTTCGGAGCTCGTGTTGCTGACTTCGGTGTTGCAAAGGCGGTCGAAGCTAATG
CTAAGGCAATCAAGTCCATGTCTGTTATTGCAGGGTCTTGTGGTTACATTGCTCCAGAATATGC
ATACACACTGCGGGTGAACGAGAAGAGTGATATATACAGCTTCGGTGTGGTCATCCTAGAGCT
TGTAACGGGGAAACGCCCTGTAGATCCCGAGTTTGGGGAAAAGGATCTGGTGAAATGGGTGT
GCAGCACGTTGGACCAAAAGGGTGTAGATCATGTCATTGACCCTAAACTTGATACTTGTTTCA
AGGAGGAGATATGCAAGGCCCTAAACATTGGCCTACTCTGCACTAGCCCTCTCCCAATTAACC
GACCCTCGATGAGACGAGTCGTTAAAATGTTGCAAGAAGTAGGTGGTGGAAACCTGCCCAAG
GCTGCTTCAAAGGATGGCAAGTTGACACCTTATTACTATGAAGAAGCATCTGATCAAGGAAGT

GTAGCTTAA

Gene Sequence:

>Solyc02g077630.2.1

AAACAAAAAAAAAAGGACAATATATGAAAATTAAAAAAAAAAACAATTCACTATAAATGTGTC
TCTAAAAAAATAAGTTACTAAAATACTAACATGTCTTAGTCAGCTTTTACTACACCCTTTTCCCA
AAAAGAAAATTGCAGTCTTTCTTCTTCTTCTTCTTTGTTCTTTCTTGATCCTCTGAAAAACCAAA
AAAAATGAAATCTTCAATTTCAATAATGTTTCTTCAAATCTTGGTTACCCTTTTTCTCCCAACTTT
GATTTTCTCACTTAATCAAGAAGGTCTTTATTTACATAATGTGAAGCTTGGATTTGATGACCCTG
ATAATGTTCTTTCAAACTGGAATGAACATGATGATACACCATGTAACTGGTTTGGTGTTTCATGT
GACAAATTTACTCGCTCTGTTACTTCATTGGACCTTTCTAATGCGAATGTTGCTGGTCCTTTTCC
CACTCTGCTTTGTCGGTTGAAGAAGCTGCGTTACATTTCCTTGTATAACAACTCGCTTAACAGT
ACTCTTCTTGAAGATTTTTCTGGGTGTGAAGCAGTAGAGCATCTTGATTTGGCGCAGAATTTCT
TGGTGGGTACACTTCCGGCGAGTTTATCTGAGCTCCCAAACTTGAAATATCTTGACTTGTCGGG
TAATAACTTCACCGGAGATATTCCGGTGAGTTTTGGTTCTTTTCAGCAGCTTGAAGTTCTTGGGT
TAGTTGGGAACTTGCTTGACGGGAGTATACCGGCGTTTCTCGGGAACGTTACGACGTTGAAGC
AGCTGAATCTGTCGTACAACCCGTTTACTACTGGTCGGATCCCGCCGGAGCTGGGAAATCTGA
CGAATCTTGAGGTTTTGTGGCTTTCTGACTGTAATTTGATTGGGGAAGTTCCTGACACATTGGG
GAGGTTGAAGAAGATTGTGGATTTGGACCTTGCTGTTAACTACTTGGATGGGCCGATTCCGAGT
TGGCTCACTGAGCTGACTAGTGCTGAACAAATTGAGCTGTATAACAACTCGTTCACCGGCGAG
TTTCCGGTGAATGGGTGGTCGAAAATGACGGCGTTGAGGCGAATCGACGTTTCGATGAACCG
GTTAACTGGTACGATTCCGAGGGAGTTGTGTGAGCTGCCACTTGAGTCACTCAATCTTTATGAA
AACCAGATGTTTGGTGAATTGCCACAAGACATTGCAAATTCACCAAACTTGTATGAGTTGCGC
CTTTTTCACAACCGTTTTAATGGGAGTTTACCTCAACATCTTGGAAAAAATTCACCTTTGTTGTG
GATTGATGTGTCGGAAAACAATTTTTCTGGTGAAATTCCGGAAAATTTATGTGGTAAAGGGTTG
TTGGAGGAGCTTTTGATGATAAATAACTTACTTTCTGGTGAAATTCCTGCCAGTTTGAGTGAAT
GCCGGAGCTTACTGCGAGTGAGATTGGCTCATAACCAGTTATCCGGTGATGTTCCGGAGGGGT
TCTGGGGTCTGCCTCACCTTTCCCTGCTTGAGCTCATGGACAATTCACTCTCCGGAGATATCGC
GAAAACTATAGCTAGTGCTTCAAATTTATCAGCTTTGATTTTGTCTAAGAACAAATTTTCAGGTT
CCATTCCTGAGGAGATTGGTTCTCTGGAAAATCTTCTTGATTTTGTGGGCAATGATAACCAGTTT
TCTGGGCCTTTACCTGCAAGTCTGGTGATTCTTGGACAATTGGGGAGGCTGGATCTTCACAAC
AATGAGTTAACTGGTAAGCTTCCGAGTGGGATTCATTCTTTGAAGAAATTGAATGAATTGAACT
TGGCAAACAATGATCTTTCTGGAGATATCCCCATGGAGATTGGGAGCTTGTCTGTTTTGAATTAT
CTTGATCTATCAGGGAACCAGTTTTCAGGGAAAATCCCACTGGAGTTGCAGAATTTGAAGCTC
AATCAGCTGAACTTGTCGAATAATGACCTTTCGGGTGATATCCCCCCTGTTTATGCAAAGGAAA
TGTATAAGAGTAGCTTTTTGGGTAATGCTGGTCTATGTGGAGACATTGAGGGCTTGTGTGAAGG
AACAGCTGAAGGTAAAACTGCTGGTTATGTTTGGTTATTGAGGTTACTATTCACCCTTGCTGGA
ATGGTGTTTGTTATTGGGGTTGCTTGGTTCTACTGGAAGTACAAGAATTTTAAGGAAGCTAAAA
GGGCTATTGATAAGTCTAAATGGACTTTAATGTCGTTTCATAAATTGGGTTTCAACGAGTATGA
AATCTTGGATGCTCTTGATGAGGATAACTTAATTGGCAGTGGCTCTTCTGGGAAGGTTTACAAG
GTTGTTCTGAGCAAGGGTGACACTGTTGCGGTGAAGAAGATATTGAGAAGTGTGAAAATAGT
AGATGATTGTAGTGATATCGAGAAAGGTAGCATTCAAGAAGATGGATTTGAAGCGGAGGTTGA
GACGTTGGGGAAGATTCGACACAAGAACATTGTTAAGCTATGGTGTTGTTGTACAACTAGGGA
TTGCAAACTTCTGGTCTATGAGTATATGCCTAATGGAAGTTTGGGTGATTTGCTACACAGCAGC
AAAAGTGGCCTTCTAGACTGGCCTATGAGATATAAGATAGCCATGGATGCAGCTGAGGGACTC

TCCTACTTGCATCATGACTGTGCTCCACCTATCGTTCACAGAGATGTTAAGTCAAATAACATCTT
GCTGGATGGTGAGTTCGGAGCTCGTGTTGCTGACTTCGGTGTTGCAAAGGCGGTCGAAGCTAA
TGCTAAGGCAATCAAGTCCATGTCTGTTATTGCAGGGTCTTGTGGTTACATTGCTCCAGGTTAG
TACAGATTAAACAATTTCAGTTTTTCAGCTTCAGTTTCATGATTTTGTGTTGGATTTGTTCATATT
GAGTTTTACTTGCTAATTGTTATGCCTGTAAGTTTCTGGATTACTGTAGCCTTTCAAAAATGAGA
TCTAGTATTACTGTAGCTATGTGGCATCTATTGTGTAGAAGGCTAGATCTATGTACTACAATATTT
CCTAGATATTCCACATTTGTTAAGTACTCTAACTAACTTCTGATAGAAATTAGCTCATATCTGGA
GTACCCCCTAATGCAGTAACAGTGCACCATTGCTCTAGATCATAGTTTGTGAACAATTTAGTAG
GCTCCACGAGTTTCCGTAACCAAGTTAAACGGTTCTGATTACATCGTTCTTGAACACAAAACA
CTGTATTAGGACTGTAGAAAGAGATATACTTAGCTTTCCACTTGTGATGTTTGAAATCTTAACA
GCTAGCTATATTGTGGGCGGCTGCTAAATTTTGTTACCAACTACCCATAATTTCCATTTCATGTG
GTCATTCTTTGTTGTGTTGTTTCCTTGCAACTGCTGAGCTGACTGAATTCTTTTTTCGCATTCCAG
AATATGCATACACACTGCGGGTGAACGAGAAGAGTGATATATACAGCTTCGGTGTGGTCATCC
TAGAGCTTGTAACGGGGAAACGCCCTGTAGATCCCGAGTTTGGGGAAAAGGATCTGGTGAAA
TGGGTGTGCAGCACGTTGGACCAAAAGGGTGTAGATCATGTCATTGACCCTAAACTTGATACT
TGTTTCAAGGAGGAGATATGCAAGGCCCTAAACATTGGCCTACTCTGCACTAGCCCTCTCCCA
ATTAACCGACCCTCGATGAGACGAGTCGTTAAAATGTTGCAAGAAGTAGGTGGTGGAAACCT
GCCCAAGGCTGCTTCAAAGGATGGCAAGTTGACACCTTATTACTATGAAGAAGCATCTGATCA
AGGAAGTGTAGCTTAAGAATGTCTCAACAAATTTTGTCATTTAACTTCTTTGTTCATCTGCTAAC
TACTCAATTCTTTCCCCACTAGATGTTTGGGTTGATTTGAAAGAAAGAATTGAAGTTAAATGAA
AAAAAGTGAGAACAAAAGAATATTAGGAAAAAAAAACAAGTAGAGTTGGAAGAATCTTGTT
TTTCATTGTAAAAATCTCATCCAACTACCAAAAGTTATTGCAGTAAGTATTACTATCTGGCACTA
AAAGATCTTCAATGTACTCTATTTTTCTTTATTCTTTCTAATCAAGCAAATTTTTAAGTTAGTTGT
GT

5,-Upstream Sequence (1kb):

>Solyc02g077630.2.1.1

GTGTTGATTGAATTTTTAAACGAACTAATCTTTCTTATTTCTTTTTATCTCATGTAACTTCTAGAA
AATCACGGTCTATCATTATGAATGACGTTATTGAAAACCATCAACCTACTCTAAAAATGTTACTT
TTTGTTCTAAGAAGGTTGAGATGAACAAAAAAAAAGGATTATAAGAAAACAAGAGTGAAAA
AAAGTAAATTGAAGTTAAGAAAGAAAGTTTAGTTGGGTTTGTAGATCACCCCTTTCTTAACTCA
TCAATCGTCTACTTGTTTCTTCAATTTACTGTTTTAAACAACTCTGTAAGAATTCGATGTGAAGG
AACTAGTCTACGAAGAAGTATCATTATTCCACAGTTGAACGGTAGGAAACTTCGTCGGAACCC
GTCCAAAGGTGGTGGATGAAGAACGTTGTAAAATTGCTGAGCAGAGTAGCTCCCAGCCAATT
AACCCTCTCCCGATCACGTCTCATCCGGTTACAAATCCCGGAACGTATAGAGGAGGAACTTTG
TTCATAGTTCAAATCCCAGTTACTGTACTAGATGTGGGAAAACCAGGTTGCACGACGTGTGGG
TAAAGTGGTCTAGGAAAAGGGGTTTGAGCCCTAGATGTCCCGCAAAGGGGCAATGTTCGAGA
TCCTACTGGTGTGGCTTCGACATATATAGTGAGAAGAGCAAGTGGGCGTCACACATACGTATA
AGACCTTACGCTTTTTTCTTAAGTCAGTCGAGTCGTCAACGTTCCTTTGTTGTGTTGTTTCTTACT
GGTGTACTTTACCTTTAATACCCATCAACCATTGTTTTAAATCGTCGGCGGGTGTTATATCGATC
GACAATTCTAAAGTTTGTAGTGTTCACCTTTCGATTCATATAGAGAAAGATGTCAGGATTATGTC
ACAAAACACAAGTTCTTGCTACATTAGTCTTGGCAAATTGAACCAATGCCTTTGAGCACCTCG
GATGATTTAACAAGTGTTTGATACTAGATCTCGTTACCACGTGA

>SlHSL1 ORF Solyc03g006300.1.1

Protein Sequence:

>Solyc03g006300.1.1
MHLQILLILLLPTLILSINQESLYLHTIKLGFDDPNGVFSNWNLHDNSSPCNWYGVKCDSLTRSVTSI
DLSNTNIAGPFPASLLCRLKYIKYISFYNNSINSTLPVEELSACKSLVHLDLAQNLLVGSLPSSLAELHE
LKYLDLTGNNFTGEIPASFGAFRRLEVLGLVENLLTGTIPPEIGNISSLKQLNLSYNPFSPGRVPPEIGN
LTNLEVLWLTDCGLIGEVPGTLRGLNKLVNLDLALNNLYGPIPSWLTELTSVEQIELYNNSFSGEFPV
NGWSNMTSLRRVDVSMNRVTGSIPNGLCELPLESLNLYENQLYGELPVAIANSPNLYELKLFGNSLN
GTLPEDLGKFSPLVWIDVSNNEFSGEIPVNLCGNGVLEEVLMIDNSFSGGIPQSLSQCRSLLRVRLAH
NKFSGDVPVEFWGLPRLSLLELTNNSFSGGIAKTIAGASNLSALILSKNEFSGNIPEEIGFLESLVDFVG
NDNKFSGSLPVSIVNLEQLGRMDFHNNELSGKFPSGVHSLKKLNELNLANNDLSGEIPREIGSLSVL
NYLDLSGNKFSGEIPVALQNLKLNQLNLSNNGLSGGIPPSYAKGMYKNSFLGNPGLCGDIGGLCDG
KDEGKTAGYVWLLRLLFVPAVLVFVVGVVSFYWKYRNYKKAKRLDRSKWTLTSFHKLDFNEFEVLR
ALDEDNLIGSGSSGKVYKVVLSNGEAAAVKKLSRNSKKVDESCDIEKGKYQDDGFDAEVETLGKIR
HKNIVRLWCCCTTRGCKLLVYEYMPNGSLGDLLHSSKSGLLDWPKRFKIATDTAEGLSYLHHDCAP
PIVHRDFKSNNILLDGEFGARVADFGVAKVIDVDDKGTMSMSVIAGSCGYIAPEYAYTLQVNEKSDI
YSFGVVVLELVTGKLPVGPEYGEKDLVKWVCATLDQKGINHVIDPKLDSCFKEDISKVLQIGLLCTSP
LPINRPPMRKVVKMLQEVGGGDQLKTALTDGKLTPYYHEDASDQGNVA

CDS Sequence:

>Solyc03g006300.1.1
ATGCATCTTCAAATCTTGCTTATACTTCTATTACCTACATTGATTCTCTCAATAAACCAAGAATCC
CTTTATTTACATACCATAAAGCTTGGATTTGATGACCCAAATGGTGTTTTTCAAACTGGAATCT
CCATGATAACTCTTCACCCTGTAACTGGTATGGAGTAAAATGCGACTCTTTAACTCGTTCTGTTA
CATCTATTGACCTCTCCAATACCAATATCGCCGGCCCATTTCCGGCTTCTCTTCTTTGCCGGCTC
AAGTATATTAAGTACATTTCATTCTATAATAACTCTATTAACTCGACACTTCCGGTGGAGGAGTT
ATCTGCTTGTAAATCTCTTGTCCATCTCGATTTAGCTCAAAATTTGTTAGTGGGTAGTCTTCCATC
GAGTTTGGCTGAGCTTCACGAGCTGAAATATCTTGATTTAACCGGGAATAACTTTACCGGCGA
AATTCCGGCGAGTTTTGGGGCTTTCCGGCGACTTGAAGTTCTGGGTTTGGTTGAAAATTTGTTA
ACTGGGACTATCCCGCCGGAGATTGGAAATATTTCGAGTTTGAAACAGCTGAATTTGTCGTAC
AACCCGTTTTCGCCGGGTCGGGTCCCGCCGGAGATTGGGAACCTTACGAATCTCGAGGTGCTT
TGGTTAACTGACTGTGGGTTAATTGGTGAGGTTCCGGGTACATTAAGGGGATTAAATAAGCTTG
TTAACTTGGACCTTGCGTTAAACAACTTGTACGGTCCGATTCCGAGCTGGCTCACTGAGTTAAC
TAGTGTTGAGCAAATTGAGCTGTATAATAACTCGTTCTCCGGCGAGTTTCCGGTGAATGGGTGG
TCGAATATGACATCGTTGAGGCGGGTCGACGTGTCGATGAATCGGGTTACCGGGTCGATCCCG
AACGGGTTGTGTGAGTTGCCACTTGAGTCGCTCAATCTTTATGAGAATCAATTGTATGGTGAGT
TACCTGTAGCCATTGCAAATTCACCCAATTTATATGAATTAAAGCTCTTTGGTAATAGTTTGAAT
GGAACTTTACCTGAAGATCTTGGTAAATTTTCGCCATTGGTATGGATTGATGTTTCAAACAATG
AGTTTTCAGGTGAAATTCCGGTGAATTTGTGCGGAAATGGAGTCTTAGAGGAGGTTTTGATGAT
AGATAACTCATTTTCCGGTGGAATTCCGCAGAGTTTAAGCCAATGCCGGAGCTTATTACGTGTG
AGGTTAGCTCATAATAAGTTCTCAGGTGATGTCCCTGTGGAATTTTGGGGGCTGCCACGCCTCT
CGCTGCTTGAGTTAACGAACAATTCATTTTCTGGTGGAATCGCGAAAACTATAGCTGGTGCATC
GAATTTATCAGCTTTGATTTTGTCAAAGAACGAATTTTCGGGTAATATTCCTGAAGAGATTGGC
TTTTTGGAAAGTCTGGTTGATTTTGTGGGAAATGATAATAAGTTTTCAGGGTCGTTGCCAGTTA
GTATAGTGAATCTTGAGCAATTGGGAAGAATGGATTTCCACAACAATGAATTAAGTGGTAAGT
TTCCTAGTGGGGTTCATTCTTTGAAGAAATTGAATGAATTGAACTTGGCAAACAATGATCTTTC
TGGAGAAATTCCCCGAGAAATTGGGAGCTTGTCTGTTTTGAACTATCTTGACCTATCAGGAAA

CAAGTTTTCGGGGGAAATTCCAGTTGCGTTGCAGAATTTGAAGCTCAATCAGCTGAATTTATCG
AATAATGGCCTTTCGGGTGGTATTCCTCCTTCATATGCAAAGGGAATGTACAAGAATAGCTTTC
TGGGGAATCCAGGTTTATGTGGAGATATTGGAGGTTTATGTGATGGAAAAGATGAAGGTAAAA
CTGCTGGTTATGTATGGTTACTGAGATTGCTTTTCGTACCTGCTGTTTTGGTGTTTGTAGTTGGGG
TAGTTTCGTTCTATTGGAAGTATAGGAATTACAAGAAAGCAAAAAGGTTGGATAGATCGAAAT
GGACCTTGACGTCGTTTCATAAGTTAGATTTCAATGAGTTTGAAGTACTGAGAGCTCTAGATGA
AGACAACTTGATTGGTAGTGGTTCTTCCGGGAAGGTTTACAAGGTCGTTTTGAGTAATGGTGA
GGCTGCTGCTGTGAAAAAACTTTCAAGAAATTCGAAAAAAGTAGATGAGAGTTGTGACATCG
AGAAAGGTAAGTATCAGGATGATGGATTTGATGCAGAGGTTGAGACATTGGGCAAAATTCGA
CACAAGAACATCGTTAGGCTATGGTGTTGTTGTACAACAAGGGGTTGCAAACTTTTGGTTTATG
AGTACATGCCTAATGGAAGCTTGGGTGATTTGCTACACAGCAGCAAAAGTGGGTTGTTGGATT
GGCCTAAGAGATTTAAGATAGCTACGGATACTGCAGAGGGACTCTCATATTTGCATCATGATTG
TGCTCCTCCGATTGTTCACAGAGACTTTAAGTCGAACAATATCTTGTTGGACGGGGAGTTTGGA
GCTCGGGTAGCTGATTTTGGTGTGGCAAAGGTGATTGATGTCGATGACAAGGGAACCATGTCT
ATGTCAGTCATTGCAGGGTCTTGCGGTTATATTGCTCCAGAATATGCATACACACTTCAGGTGA
ACGAGAAGAGTGATATATATAGTTTTGGCGTGGTAGTCCTCGAGCTAGTGACAGGGAAACTCC
CTGTAGGTCCCGAATACGGGGAAAAGGATTTGGTGAAGTGGGTTTGCGCTACTCTAGACCAGA
AGGGTATAAATCATGTTATTGACCCGAAACTCGACTCTTGTTTCAAGGAGGACATAAGCAAAG
TCCTACAAATTGGCCTCCTCTGCACTAGCCCCCTCCCAATCAACCGACCCCCGATGAGAAAAG
TCGTAAAAATGCTGCAGGAAGTTGGTGGCGGAGACCAGCTCAAGACAGCGTTAACAGATGGC
AAGTTGACCCCTTACTACCACGAAGACGCATCAGATCAAGGAAATGTAGCTTAA

Gene Sequence:

>Solyc03g006300.1.1

ATGCATCTTCAAATCTTGCTTATACTTCTATTACCTACATTGATTCTCTCAATAAACCAAGAATCC
CTTTATTTACATACCATAAAGCTTGGATTTGATGACCCAAATGGTGTTTTTTCAAACTGGAATCT
CCATGATAACTCTTCACCCTGTAACTGGTATGGAGTAAAATGCGACTCTTTAACTCGTTCTGTTA
CATCTATTGACCTCTCCAATACCAATATCGCCGGCCCATTTCCGGCTTCTCTTCTTTGCCGGCTC
AAGTATATTAAGTACATTTCATTCTATAATAACTCTATTAACTCGACACTTCCGGTGGAGGAGTT
ATCTGCTTGTAAATCTCTTGTCCATCTCGATTTAGCTCAAAATTTGTTAGTGGGTAGTCTTCCATC
GAGTTTGGCTGAGCTTCACGAGCTGAAATATCTTGATTTAACCGGGAATAACTTTACCGGCGA
AATTCCGGCGAGTTTTGGGGCTTTCCGGCGACTTGAAGTTCTGGGTTTGGTTGAAAATTTGTTA
ACTGGGACTATCCCGCCGGAGATTGGAAATATTTCGAGTTTGAAACAGCTGAATTTGTCGTAC
AACCCGTTTTCGCCGGGTCGGGTCCCGCCGGAGATTGGGAACCTTACGAATCTCGAGGTGCTT
TGGTTAACTGACTGTGGGTTAATTGGTGAGGTTCCGGGTACATTAAGGGGATTAAATAAGCTTG
TTAACTTGGACCTTGCGTTAAACAACTTGTACGGTCCGATTCCGAGCTGGCTCACTGAGTTAAC
TAGTGTTGAGCAAATTGAGCTGTATAATAACTCGTTCTCCGGCGAGTTTCCGGTGAATGGGTGG
TCGAATATGACATCGTTGAGGCGGGTCGACGTGTCGATGAATCGGGTTACCGGGTCGATCCCG
AACGGGTTGTGTGAGTTGCCACTTGAGTCGCTCAATCTTTATGAGAATCAATTGTATGGTGAGT
TACCTGTAGCCATTGCAAATTCACCCAATTTATATGAATTAAAGCTCTTTGGTAATAGTTTGAAT
GGAACTTTACCTGAAGATCTTGGTAAATTTTCGCCATTGGTATGGATTGATGTTTCAAACAATG
AGTTTTCAGGTGAAATTCCGGTGAATTTGTGCGGAAATGGAGTCTTAGAGGAGGTTTTGATGAT
AGATAACTCATTTTCCGGTGGAATTCCGCAGAGTTTAAGCCAATGCCGGAGCTTATTACGTGTG
AGGTTAGCTCATAATAAGTTCTCAGGTGATGTCCCTGTGGAATTTTGGGGGCTGCCACGCCTCT
CGCTGCTTGAGTTAACGAACAATTCATTTTCTGGTGGAATCGCGAAAACTATAGCTGGTGCATC

GAATTTATCAGCTTTGATTTTGTCAAAGAACGAATTTTCGGGTAATATTCCTGAAGAGATTGGC
TTTTTGGAAAGTCTGGTTGATTTTGTGGGAAATGATAATAAGTTTTCAGGGTCGTTGCCAGTTA
GTATAGTGAATCTTGAGCAATTGGGAAGAATGGATTTCCACAACAATGAATTAAGTGGTAAGT
TTCCTAGTGGGGTTCATTCTTTGAAGAAATTGAATGAATTGAACTTGGCAAACAATGATCTTTC
TGGAGAAATTCCCCGAGAAATTGGGAGCTTGTCTGTTTTGAACTATCTTGACCTATCAGGAAA
CAAGTTTTCGGGGGAAATTCCAGTTGCGTTGCAGAATTTGAAGCTCAATCAGCTGAATTTATCG
AATAATGGCCTTTCGGGTGGTATTCCTCCTTCATATGCAAAGGGAATGTACAAGAATAGCTTTC
TGGGGAATCCAGGTTTATGTGGAGATATTGGAGGTTTATGTGATGGAAAAGATGAAGGTAAAA
CTGCTGGTTATGTATGGTTACTGAGATTGCTTTTCGTACCTGCTGTTTTGGTGTTTGTAGTTGGGG
TAGTTTCGTTCTATTGGAAGTATAGGAATTACAAGAAAGCAAAAAGGTTGGATAGATCGAAAT
GGACCTTGACGTCGTTTCATAAGTTAGATTTCAATGAGTTTGAAGTACTGAGAGCTCTAGATGA
AGACAACTTGATTGGTAGTGGTTCTTCCGGGAAGGTTTACAAGGTCGTTTTGAGTAATGGTGA
GGCTGCTGCTGTGAAAAAACTTTCAAGAAATTCGAAAAAAGTAGATGAGAGTTGTGACATCG
AGAAAGGTAAGTATCAGGATGATGGATTTGATGCAGAGGTTGAGACATTGGGCAAAATTCGA
CACAAGAACATCGTTAGGCTATGGTGTTGTTGTACAACAAGGGGTTGCAAACTTTTGGTTTATG
AGTACATGCCTAATGGAAGCTTGGGTGATTTGCTACACAGCAGCAAAAGTGGGTTGTTGGATT
GGCCTAAGAGATTTAAGATAGCTACGGATACTGCAGAGGGACTCTCATATTTGCATCATGATTG
TGCTCCTCCGATTGTTCACAGAGACTTTAAGTCGAACAATATCTTGTTGGACGGGGAGTTTGGA
GCTCGGGTAGCTGATTTTGGTGTGGCAAAGGTGATTGATGTCGATGACAAGGGAACCATGTCT
ATGTCAGTCATTGCAGGGTCTTGCGGTTATATTGCTCCAGGTTAGAGGATTGATCCTTCACTTTG
TGAGTTTTACAAACATGATTTTATTGAAATTGTTTTATGCCTTCCATTGCAAATTGTAGTTAAGAT
GTATGCCTAAATATAACATATGTCATATATTGTTAAGAGTATATTATAATTAAGCCTCGAATAAGG
TCTGTTGTACACTCTATCCTCCTCTGACCTCGCTTGTGGGATTACATTGGGTATATTGTCGTTGAT
TATTGTTAAACCTCCAATATATATGAGTAATTTTGGTCCATTGCTCGAATCCTTCAAAAAATGTG
GCATGTCTTTGTTGGATTCTCCCAAAGTTGATCAATTTTAGAGGATCCAATACAGGTGCAACGA
CATTTGGAGGATCCGAGCAACTTAGAACTAACTTGATTGCCCTTGTACATTTAACACTAAGGT
TTGCTCCAAGAACAGGTCATTCTTTGTTGACATTTGCTTTCTGCTTCTCGTAGAGCTCCTAAGGT
TGAACTAAATTGGGTTCGTCTTGCAGAATATGCATACACACTTCAGGTGAACGAGAAGAGTGA
TATATATAGTTTTGGCGTGGTAGTCCTCGAGCTAGTGACAGGGAAACTCCCTGTAGGTCCCGAA
TACGGGGAAAAGGATTTGGTGAAGTGGGTTTGCGCTACTCTAGACCAGAAGGGTATAAATCAT
GTTATTGACCCGAAACTCGACTCTTGTTTCAAGGAGGACATAAGCAAAGTCCTACAAATTGGC
CTCCTCTGCACTAGCCCCCTCCCAATCAACCGACCCCCGATGAGAAAAGTCGTAAAAATGCTG
CAGGAAGTTGGTGGCGGAGACCAGCTCAAGACAGCGTTAACAGATGGCAAGTTGACCCCTTA
CTACCACGAAGACGCATCAGATCAAGGAAATGTAGCTTAA

5,-Upstream Sequence (1kb):

>Solyc03g006300.1.1

ATTCGATGTAAAGGAACTAGACTACGCAGAAGCACCATCATTCCCCAGTTGAACGGTAGACA
ATTGCGACAGAACTCGACCAGAGGCGGTGGTTGAAGGACGTCGTAAAAATGCTGAAAAGAG
TAGCCCCCAGCCAACTAACCCTCCCCCGATCACGTCTCCTCCGGTTAAACATCCTGAAACGAA
TACAGGAGGAACTTTGTTCTCAGCTCAAAGCCCAGTTATTGTACTAAATATGGGAAGACCAGA
TCTCATCGCGTTTGGGTGAAGTGGTTTAGGAAAAGGGGCATAAGCCCTGGATGTCCCTCAAAG
GGACAGTGATCGAGCTCCTGATGGTGCGGTTTTGATATATATAGTGAGAAGAGCAAGTGGACT
TCACACATACGTATAAGACGTTCTGCTTGGGTTAAATCAAGTTGGAATCCTCGAGATGCTCTTC
GTCTTTCGTTTACAGTTGTTTCTTACTGGACAAGAACCTCGTTTGGAATCACAATTTACATGTTC

CCGTTAGTTCAATCAAGATTCAACGAGCCTAGGAGGTTTTACAGCAACGTGGACATAACCTAG
GAGATTTTAACTAGTTGAAACCCTCTTAGGTTGTTTCTGTACGGTGTAAAAAACTTCCTAAGCT
CGTTACCTGGTTTTAATGAGTATATATAACCTCCAAATTGTTATTAGTTGCTGTTATATGGGTTAC
ATTAGGGTGTTCGCTCCAGTCTCCTCCTATCTCACATGTTGTCTGGAATAAGCTCCGAATTAATA
TTATATGAGAATTGTTATATACTGTATACAATATAAATCCGTATGTAGAATTGATGTTAAACGTTA
CCTTCCGTATTTTGTTAAAGTTATTTTAGTACAAACATTTTGAGTGTTTCACTTCCTAGTTAGGAG
ATTGGACCTCGTTATATTGGCGTTCTGGGACGTTACTGACTGTATCTGTACCAAGGGAACAGTA
GCTGTAGTTAGTGGAAACGGTGTGGTTTTAGTCGATGGGCTC

>SlHSL2 ORF Solyc07g053600.2.1

Protein Sequence:

>Solyc07g053600.2.1
MQMKLLLFFLSTFPLIFALNQDGLYLQRLKHSLSSSDQGVFSTWYENDPTPCNWTGVTCNDAGDSP
SVIAVNLSGASLVGPFPGFLCHLTSLSSLSLSNNFINSTLPVSISECGSLTYLDISQNLIGGTIPDTISDLP
YLRYLDLSGCYFSGNIPASLGRFRQLETLILTENILTGEVPAALGNVTSLKTLELAYNPFAPSLFPPELG
NLTNLETLWLSMCNLVGSIPKSIEKLSRLTNFDVSNNGLVGSIPSAIFQLNSIVQIELYNNSLTGKLPS
GWSNLTRLRRFDVSTNKLNGTIPNELCELPLESLNLFENQFEGLIPESIANSPNLYELKLFSNRFSGSL
PSELGKNSALQYLDVSYNTFSGKIPESLCEIGALEDLIVIYNSFSGNIPASLGNCRSLLRIRFRSNKLFGE
VPTDFWSLPHVYLLDLFGNAFSGNISHMISGAKNLSNLQISRNKFSGVIPSEVGKLKNLVEFSASHNE
LTGELPDTLVQLGQLGTLDLSFNELSGKIPLGIHTMKQLSELDLANNGFSGEIPEQIGTLPVLNYLDL
SGNYFSGEIPLSLQSLKLNKLNLSNNQLSGMIPAVFDKGLYRDSFRGNPGLCQGVAGLCATKGRGQ
HEGYLWTLRAIYTVAGFVFLVGIAMFIWKYQKFKKIKKGNTMTKWTSFHKLGFSEFEIPVGLDEAN
VIGNGASGRVYKAVLSNGEAVAVKKLWERTVKDETPYGALESDKDEFEIEVETLGKIRHKNIVKLW
CCCDTGDSKLLVYEYMPNGSLGDLLHSCKAKLLDWPLRFKIALDAAEGLSYLHHGCVPPIVHRDV
KSNNILLDDEFGAKISDFGVAKIVKAGSKGGVESMSVIAGSCGYIAPEYAYTLHVNEKSDIYSFGVVI
LELVTGKRPVSPEFGEKDLTTWVHTTLNEKGVDQLLDPNLNSSFKKHICKVLDVGLCCLNQTPAN
RPSMHRVVKMLQESVPCNVPEIKNKNGKLSPQYFPKSV

CDS Sequence:

>Solyc07g053600.2.1
ATGCAAATGAAACTTTTACTCTTCTTTCTGAGTACATTCCCTTTGATTTTTGCTTTAAATCAAGA
TGGGTTGTATCTTCAAAGACTGAAACACTCGTTGTCTAGCTCAGATCAAGGGGTATTTTCTACT
TGGTATGAAAATGATCCTACCCCATGTAACTGGACAGGTGTTACCTGTAATGACGCCGGAGA
TTCTCCCTCCGTTATCGCTGTTAACCTCTCCGGTGCATCTCTTGTCGGACCCTTTCCGGGTTTCC
TCTGCCATCTCACTTCACTTTCATCACTCTCACTTTCGAATAATTTTATTAATTCTACTCTTCCG
GTTTCTATTTCTGAATGTGGTAGCCTCACGTACCTTGACATTTCTCAGAATCTCATCGGTGGAA
CTATCCCTGACACTATTTCCGATCTTCCTTACCTCAGGTACCTTGATCTTAGTGGATGCTATTTT
TCAGGGAATATTCCGGCAAGTTTGGGAAGATTCAGGCAACTAGAGACTCTTATTCTGACTGA
GAACATTCTAACTGGTGAAGTTCCAGCTGCATTAGGTAATGTAACGAGTCTCAAGACACTTG
AACTTGCTTACAACCCTTTTGCACCGAGTCTGTTTCCTCCTGAACTCGGTAACTTAACGAATCT
TGAGACATTATGGCTAAGCATGTGTAATCTTGTTGGTTCAATCCCAAAAAGTATTGAGAAATT
GAGTCGATTGACTAATTTTGATGTGTCCAATAATGGACTAGTTGGGTCAATACCAAGTGCAAT
TTTCCAGCTTAATAGTATTGTTCAAATTGAGCTCTACAATAATTCTCTTACCGGAAAATTGCCT
TCGGGATGGTCTAACTTGACCAGGTTGAGAAGATTCGATGTATCGACTAACAAGTTAAATGG
GACTATTCCTAATGAGTTGTGTGAGCTGCCACTTGAGTCACTCAATCTATTTGAGAATCAATTT
GAGGGGCTTATTCCAGAAAGTATAGCTAACTCTCCGAATCTGTATGAGCTGAAGTTATTCTCT

AACAGATTTTCTGGTTCATTGCCTAGTGAACTGGGGAAGAACTCGGCTTTACAGTATCTTGAT
GTTTCATACAATACATTTTCTGGTAAAATTCCTGAAAGTTTATGTGAGATTGGAGCTTTAGAG
GATCTTATAGTTATATATAATTCGTTCTCTGGGAATATTCCGGCCAGTCTTGGCAACTGCCGGA
GTTTACTTAGGATCAGGTTCCGGTCTAATAAGCTATTCGGGGAAGTCCCAACTGACTTTTGGA
GTTTGCCTCATGTTTATCTCTTGGACCTTTTTGGCAATGCATTTTCAGGAAATATATCACACAT
GATTTCTGGTGCCAAAAATTTATCTAACCTCCAAATATCAAGAAACAAATTCTCAGGGGTTAT
ACCTAGTGAAGTAGGAAAGTTGAAGAACTTAGTTGAGTTTTCCGCGAGTCATAATGAGCTAA
CGGGAGAACTTCCAGACACATTAGTGCAGCTAGGGCAGTTAGGAACCCTTGATCTTAGTTTC
AATGAGCTATCAGGGAAAATCCCCTTGGGAATTCACACAATGAAGCAACTCAGTGAGCTTGA
CTTGGCAAACAATGGATTTTCTGGGGAAATTCCGGAGCAAATTGGGACTTTGCCAGTGCTTAA
TTATCTTGATCTTTCTGGGAATTACTTCTCAGGGGAAATCCCACTTAGTCTGCAAAGCTTGAAG
CTTAATAAGCTAAATTTGTCTAACAATCAGCTGTCAGGGATGATTCCTGCAGTTTTTGATAAG
GGTCTTTATAGAGACAGCTTTCGAGGTAATCCAGGTTTGTGTCAAGGTGTTGCTGGTCTTTGTG
CTACCAAAGGTAGAGGACAGCATGAAGGATACTTATGGACTTTGAGAGCTATCTACACAGTT
GCTGGCTTCGTTTTCCTTGTCGGGATTGCTATGTTCATTTGGAAGTACCAGAAATTCAAGAAG
ATTAAAAAAGGAAACACTATGACAAAGTGGACATCATTCCATAAGCTTGGATTTAGTGAATT
TGAAATACCCGTTGGCCTAGATGAAGCTAATGTAATTGGCAATGGAGCTTCAGGAAGAGTGT
ACAAAGCTGTCCTAAGCAATGGTGAGGCAGTAGCTGTCAAGAAGCTATGGGAGAGAACAGT
TAAAGATGAAACCCCGTATGGTGCTCTTGAGTCTGATAAAGACGAGTTTGAAATTGAAGTTG
AAACTCTGGGTAAAATTAGGCACAAGAATATTGTGAAATTGTGGTGCTGTTGCGATACTGGG
GATAGCAAGCTCTTGGTATATGAGTACATGCCAAATGGAAGTTTGGGTGATTTGCTGCACAGT
TGCAAGGCCAAATTGTTGGATTGGCCGTTGAGGTTCAAGATAGCTTTAGATGCAGCTGAGGG
GCTCTCTTATTTGCACCATGGTTGTGTTCCTCCAATTGTTCACCGTGATGTTAAGTCAAACAAC
ATATTGCTGGATGATGAGTTTGGAGCCAAAATTTCAGATTTTGGTGTGGCAAAAATTGTTAAA
GCAGGCAGCAAAGGTGGCGTCGAATCCATGTCTGTAATTGCTGGTTCCTGTGGTTACATTGCT
CCAGAGTATGCATATACTCTTCATGTGAATGAAAAAAGTGACATATATAGCTTTGGAGTGGTC
ATTTTGGAGCTGGTGACAGGCAAACGACCAGTCAGTCCAGAATTCGGAGAGAAAGATCTAA
CTACTTGGGTACACACAACGTTGAACGAGAAAGGAGTTGATCAGTTGCTCGATCCAAATCTA
AACTCCAGCTTCAAAAAACATATATGCAAGGTTCTTGATGTTGGTCTATGCTGTCTTAACCAG
ACTCCAGCTAATCGCCCCTCAATGCACAGAGTGGTGAAAATGCTCCAAGAATCAGTTCCTTGT
AACGTGCCAGAAATCAAAAACAAGAACGGTAAACTTTCCCCTCAGTACTTTCCAAAGTCAGT
CTAG

Gene Sequence:

>Solyc07g053600.2.1

TACACTATTTTAACAAGCTCAACAAACACCCATTTTCACCATTGTTACAGAAAAATCTCTTCA
CCTTCTTCTTCTCGTACATGAAAAACCATGCAAATGAAACTTTTACTCTTCTTTCTGAGTACAT
TCCCTTTGATTTTTGCTTTAAATCAAGATGGGTTGTATCTTCAAAGACTGAAACACTCGTTGTC
TAGCTCAGATCAAGGGGTATTTTCTACTTGGTATGAAAATGATCCTACCCCATGTAACTGGAC
AGGTGTTACCTGTAATGACGCCGGAGATTCTCCCTCCGTTATCGCTGTTAACCTCTCCGGTGCA
TCTCTTGTCGGACCCTTTCCGGGTTTCCTCTGCCATCTCACTTCACTTTCATCACTCTCACTTTC
GAATAATTTTATTAATTCTACTCTTCCGGTTTCTATTTCTGAATGTGGTAGCCTCACGTACCTTG
ACATTTCTCAGAATCTCATCGGTGGAACTATCCCTGACACTATTTCCGATCTTCCTTACCTCAG
GTAACTCAGTTTGAAGCTCAATTTTACATTCCCCTGTGTTTTATTTCCTTCAAATTTTCGAAAAA
TAGCAACTATAGTTAGCGTAAATAACATGGGTATGTATAGAAAAGTATAGTCCAGTGCACAA

AACATCTCGTAGCAGAGGATGTAAGCAATGATTCAGCTAAATACACATCATCCGCCTGGATT
TACTTACTGGAAACTCAGAATTACTAGTATTGAGTATTTGAATAAAAAATGCAAATGAAACA
AAATGAATATTATGATTTTTATTGAAGATTTTGTTATTTGTTTTTTTAATAGTATGTTATGATCTT
TTTATTGCAGGTACCTTGATCTTAGTGGATGCTATTTTTCAGGGAATATTCCGGCAAGTTTGGG
AAGATTCAGGCAACTAGAGACTCTTATTCTGACTGAGAACATTCTAACTGGTGAAGTTCCAGC
TGCATTAGGTAATGTAACGAGTCTCAAGACACTTGAACTTGCTTACAACCCTTTTGCACCGAG
TCTGTTTCCTCCTGAACTCGGTAACTTAACGAATCTTGAGACATTATGGCTAAGCATGTGTAAT
CTTGTTGGTTCAATCCCAAAAAGTATTGAGAAATTGAGTCGATTGACTAATTTTGATGTGTCC
AATAATGGACTAGTTGGGTCAATACCAAGTGCAATTTTCCAGCTTAATAGTATTGTTCAAATT
GAGCTCTACAATAATTCTCTTACCGGAAAATTGCCTTCGGGATGGTCTAACTTGACCAGGTTG
AGAAGATTCGATGTATCGACTAACAAGTTAAATGGGACTATTCCTAATGAGTTGTGTGAGCTG
CCACTTGAGTCACTCAATCTATTTGAGAATCAATTTGAGGGGCTTATTCCAGAAAGTATAGCT
AACTCTCCGAATCTGTATGAGCTGAAGTTATTCTCTAACAGATTTTCTGGTTCATTGCCTAGTG
AACTGGGGAAGAACTCGGCTTTACAGTATCTTGATGTTTCATACAATACATTTTCTGGTAAAA
TTCCTGAAAGTTTATGTGAGATTGGAGCTTTAGAGGATCTTATAGTTATATATAATTCGTTCTC
TGGGAATATTCCGGCCAGTCTTGGCAACTGCCGGAGTTTACTTAGGATCAGGTTCCGGTCTAA
TAAGCTATTCGGGGAAGTCCCAACTGACTTTTGGAGTTTGCCTCATGTTTATCTCTTGGACCTT
TTTGGCAATGCATTTTCAGGAAATATATCACACATGATTTCTGGTGCCAAAAATTTATCTAAC
CTCCAAATATCAAGAAACAAATTCTCAGGGGTTATACCTAGTGAAGTAGGAAAGTTGAAGAA
CTTAGTTGAGTTTTCCGCGAGTCATAATGAGCTAACGGGAGAACTTCCAGACACATTAGTGCA
GCTAGGGCAGTTAGGAACCCTTGATCTTAGTTTCAATGAGCTATCAGGGAAAATCCCCTTGGG
AATTCACACAATGAAGCAACTCAGTGAGCTTGACTTGGCAAACAATGGATTTTCTGGGGAAA
TTCCGGAGCAAATTGGGACTTTGCCAGTGCTTAATTATCTTGATCTTTCTGGGAATTACTTCTC
AGGGGAAATCCCACTTAGTCTGCAAAGCTTGAAGCTTAATAAGCTAAATTTGTCTAACAATC
AGCTGTCAGGGATGATTCCTGCAGTTTTTGATAAGGGTCTTTATAGAGACAGCTTTCGAGGTA
ATCCAGGTTTGTGTCAAGGTGTTGCTGGTCTTTGTGCTACCAAAGGTAGAGGACAGCATGAAG
GATACTTATGGACTTTGAGAGCTATCTACACAGTTGCTGGCTTCGTTTTCCTTGTCGGGATTGC
TATGTTCATTTGGAAGTACCAGAAATTCAAGAAGATTAAAAAAGGAAACACTATGACAAAGT
GGACATCATTCCATAAGCTTGGATTTAGTGAATTTGAAATACCCGTTGGCCTAGATGAAGCTA
ATGTAATTGGCAATGGAGCTTCAGGAAGAGTGTACAAAGCTGTCCTAAGCAATGGTGAGGCA
GTAGCTGTCAAGAAGCTATGGGAGAGAACAGTTAAAGATGAAACCCCGTATGGTGCTCTTGA
GTCTGATAAAGACGAGTTTGAAATTGAAGTTGAAACTCTGGGTAAAATTAGGCACAAGAATA
TTGTGAAATTGTGGTGCTGTTGCGATACTGGGGATAGCAAGCTCTTGGTATATGAGTACATGC
CAAATGGAAGTTTGGGTGATTTGCTGCACAGTTGCAAGGCCAAATTGTTGGATTGGCCGTTGA
GGTTCAAGATAGCTTTAGATGCAGCTGAGGGGCTCTCTTATTTGCACCATGGTTGTGTTCCTCC
AATTGTTCACCGTGATGTTAAGTCAAACAACATATTGCTGGATGATGAGTTTGGAGCCAAAAT
TTCAGATTTTGGTGTGGCAAAAATTGTTAAAGCAGGCAGCAAAGGTGGCGTCGAATCCATGT
CTGTAATTGCTGGTTCCTGTGGTTACATTGCTCCAGGTATTGCTTTGTCCAAACTGTATCACTG
GCTTTAGTCATTTCTATCGATATATTAACACATCTAGCTTATATTTTTCGGAAAGATTGCAATT
TCATTTCAAAGTAACAAAAACTAACATAAGTGAAATTTATTGTTTGCAGAGTATGCATATACT
CTTCATGTGAATGAAAAAAGTGACATATATAGCTTTGGAGTGGTCATTTTGGAGCTGGTGACA
GGCAAACGACCAGTCAGTCCAGAATTCGGAGAGAAAGATCTAACTACTTGGGTACACACAA
CGTTGAACGAGAAAGGAGTTGATCAGTTGCTCGATCCAAATCTAAACTCCAGCTTCAAAAAA
CATATATGCAAGGTTCTTGATGTTGGTCTATGCTGTCTTAACCAGACTCCAGCTAATCGCCCCT

CAATGCACAGAGTGGTGAAAATGCTCCAAGAATCAGTTCCTTGTAACGTGCCAGAAATCAAA
AACAAGAACGGTAAACTTTCCCCTCAGTACTTTCCAAAGTCAGTCTAGTTAATATATAATATC
TAGTAATAGAGCAGGAGTTTTTCCTGTCAAGAATTGGCAAGGAAGTAGCTGCATAACGAAAA
CTTTACTAGTCTTTTTCAAGAATTAAGCATATTTTGTACTCTTTGAATTCTACAACCCTTTATGA
ATCTATATTACGATGATTCATCATTCATCATGAATTAACAAATTTTACGCTAGCTGTCAGCCTA
CGGCAACACAAGTTGGATACTGAAAGTTGTAAGTATTTCTTAACTAATGCATTTGATTAAGCT
TGTTAAA

5,-Upstream Sequence (1kb):

>Solyc07g053600.2.1.1

AATTGTTCGAATTAGTTTACGTAATCAATTCTTTATGAATGTTGAAAGTCATAGGTTGAACAC
AACGGCATCCGACTGTCGATCGCATTTTAAACAATTAAGTACTACTTACTACTTAGTAGCATT
ATATCTAAGTATTTCCCAACATCTTAAGTTTCTCATGTTTTATACGAATTAAGAACTTTTTCTGA
TCATTTCAAAAGCAATACGTCGATGAAGGAACGGTTAAGAACTGTCCTTTTTGAGGACGAGA
TAATGATCTATAATATATAATTGATCTGACTGAAACCTTTCATGACTCCCCTTTCAAATGGCA
AGAACAAAAACTAAAGACCGTGCAATGTTCCTTGACTAAGAACCTCGTAAAAGTGGTGAGA
CACGTAACTCCCCGCTAATCGACCTCAGACCAATTCTGTCGTATCTGGTTGTAGTTCTTGGAA
CGTATATACAAAAAACTTCGACCTCAAATCTAAACCTAGCTCGTTGACTAGTTGAGGAAAGA
GCAAGTTGCAACACACATGGGTTCATCAATCTAGAAAGAGAGGCTTAAGACCTGACTGACCA
GCAAACGGACAGTGGTCGAGGTTTTACTGGTGAGGTTTCGATATATACAGTGAAAAAAGTAA
GTGTACTTCTCATATACGTATGAGACGTTTGTTATTTAAAGTGAATACAATCAAAAACAATGA
AACTTTACTTTAACGTTAGAAAGGCTTTTTATATTCGATCTACACAATTATATAGCTATCTTTA
CTGATTTCGGTCACTATGTCAAACCTGTTTCGTTATGGACCTCGTTACATTGGTGTCCTTGGTC
GTTAATGTCTGTACCTAAGCTGCGGTGGAAACGACGGACGAAATTGTTAAAAACGGTGTGGT
TTTAGACTTTAAAACCGAGGTTTGAGTAGTAGGTCGTTATACAACAAACTGAATTGTAGTGCC
ACTTGTTAACCTCCTTGTGTTGGTACCACGTTTATTCTCTCGGGGAGTCGACGTAGAT

>SlHSL3 ORF Solyc03g006300.1.1

Protein Sequence:

>Solyc03g006300.1.1

MHLQILLILLLPTLILSINQESLYLHTIKLGFDDPNGVFSNWNLHDNSSPCNWYGVKCDSLTRSVTSI
DLSNTNIAGPFPASLLCRLKYIKYISFYNNSINSTLPVEELSACKSLVHLDLAQNLLVGSLPSSLAELH
ELKYLDLTGNNFTGEIPASFGAFRRLEVLGLVENLLTGTIPPEIGNISSLKQLNLSYNPFSPGRVPPEIG
NLTNLEVLWLTDCGLIGEVPGTLRGLNKLVNLDLALNNLYGPIPSWLTELTSVEQIELYNNSFSGEF
PVNGWSNMTSLRRVDVSMNRVTGSIPNGLCELPLESLNLYENQLYGELPVAIANSPNLYELKLFGN
SLNGTLPEDLGKFSPLVWIDVSNNEFSGEIPVNLCGNGVLEEVLMIDNSFSGGIPQSLSQCRSLLRVR
LAHNKFSGDVPVEFWGLPRLSLLELTNNSFSGGIAKTIAGASNLSALILSKNEFSGNIPEEIGFLESLV
DFVGNDNKFSGSLPVSIVNLEQLGRMDFHNNELSGKFPSGVHSLKKLNELNLANNDLSGEIPREIGS
LSVLNYLDLSGNKFSGEIPVALQNLKLNQLNLSNNGLSGGIPPSYAKGMYKNSFLGNPGLCGDIGG
LCDGKDEGKTAGYVWLLRLLFVPAVLVFVVGVVSFYWKYRNYKKAKRLDRSKWTLTSFHKLDFNE
FEVLRALDEDNLIGSGSSGKVYKVVLSNGEAAAVKKLSRNSKKVDESCDIEKGKYQDDGFDAEVET
LGKIRHKNIVRLWCCCTTRGCKLLVYEYMPNGSLGDLLHSSKSGLLDWPKRFKIATDTAEGLSYLH
HDCAPPIVHRDFKSNNILLDGEFGARVADFGVAKVIDVDDKGTMSMSVIAGSCGYIAPEYAYTLQV
NEKSDIYSFGVVVLELVTGKLPVGPEYGEKDLVKWVCATLDQKGINHVIDPKLDSCFKEDISKVLQI
GLLCTSPLPINRPPMRKVVKMLQEVGGGDQLKTALTDGKLTPYYHEDASDQGNVA

CDS Sequence:

>Solyc03g006300.1.1

ATGCATCTTCAAATCTTGCTTATACTTCTATTACCTACATTGATTCTCTCAATAAACCAAGAAT
CCCTTTATTTACATACCATAAAGCTTGGATTTGATGACCCAAATGGTGTTTTTTCAAACTGGAA
TCTCCATGATAACTCTTCACCCTGTAACTGGTATGGAGTAAAATGCGACTCTTTAACTCGTTCT
GTTACATCTATTGACCTCTCCAATACCAATATCGCCGGCCCATTTCCGGCTTCTCTTCTTTGCC
GGCTCAAGTATATTAAGTACATTTCATTCTATAATAACTCTATTAACTCGACACTTCCGGTGGA
GGAGTTATCTGCTTGTAAATCTCTTGTCCATCTCGATTTAGCTCAAAATTTGTTAGTGGGTAGT
CTTCCATCGAGTTTGGCTGAGCTTCACGAGCTGAAATATCTTGATTTAACCGGGAATAACTTT
ACCGGCGAAATTCCGGCGAGTTTTGGGGCTTTCCGGCGACTTGAAGTTCTGGGTTTGGTTGAA
AATTTGTTAACTGGGACTATCCCGCCGGAGATTGGAAATATTTCGAGTTTGAAACAGCTGAAT
TTGTCGTACAACCCGTTTTCGCCGGGTCGGGTCCCGCCGGAGATTGGGAACCTTACGAATCTC
GAGGTGCTTTGGTTAACTGACTGTGGGTTAATTGGTGAGGTTCCGGGTACATTAAGGGGATTA
AATAAGCTTGTTAACTTGGACCTTGCGTTAAACAACTTGTACGGTCCGATTCCGAGCTGGCTC
ACTGAGTTAACTAGTGTTGAGCAAATTGAGCTGTATAATAACTCGTTCTCCGGCGAGTTTCCG
GTGAATGGGTGGTCGAATATGACATCGTTGAGGCGGGTCGACGTGTCGATGAATCGGGTTAC
CGGGTCGATCCCGAACGGGTTGTGTGAGTTGCCACTTGAGTCGCTCAATCTTTATGAGAATCA
ATTGTATGGTGAGTTACCTGTAGCCATTGCAAATTCACCCAATTTATATGAATTAAAGCTCTTT
GGTAATAGTTTGAATGGAACTTTACCTGAAGATCTTGGTAAATTTTCGCCATTGGTATGGATTG
ATGTTTCAAACAATGAGTTTTCAGGTGAAATTCCGGTGAATTTGTGCGGAAATGGAGTCTTAG
AGGAGGTTTTGATGATAGATAACTCATTTTCCGGTGGAATTCCGCAGAGTTTAAGCCAATGCC
GGAGCTTATTACGTGTGAGGTTAGCTCATAATAAGTTCTCAGGTGATGTCCCTGTGGAATTTTG
GGGGCTGCCACGCCTCTCGCTGCTTGAGTTAACGAACAATTCATTTTCTGGTGGAATCGCGAA
AACTATAGCTGGTGCATCGAATTTATCAGCTTTGATTTTGTCAAAGAACGAATTTTCGGGTAA
TATTCCTGAAGAGATTGGCTTTTTGGAAAGTCTGGTTGATTTTGTGGGAAATGATAATAAGTTT
TCAGGGTCGTTGCCAGTTAGTATAGTGAATCTTGAGCAATTGGGAAGAATGGATTTCCACAAC
AATGAATTAAGTGGTAAGTTTCCTAGTGGGGTTCATTCTTTGAAGAAATTGAATGAATTGAAC
TTGGCAAACAATGATCTTTCTGGAGAAATTCCCCGAGAAATTGGGAGCTTGTCTGTTTTGAAC
TATCTTGACCTATCAGGAAACAAGTTTTCGGGGGAAATTCCAGTTGCGTTGCAGAATTTGAAG
CTCAATCAGCTGAATTTATCGAATAATGGCCTTTCGGGTGGTATTCCTCCTTCATATGCAAAG
GGAATGTACAAGAATAGCTTTCTGGGGAATCCAGGTTTATGTGGAGATATTGGAGGTTTATGT
GATGGAAAAGATGAAGGTAAAACTGCTGGTTATGTATGGTTACTGAGATTGCTTTTCGTACCT
GCTGTTTTGGTGTTTGTAGTTGGGGTAGTTTCGTTCTATTGGAAGTATAGGAATTACAAGAAAG
CAAAAAGGTTGGATAGATCGAAATGGACCTTGACGTCGTTTCATAAGTTAGATTTCAATGAGT
TTGAAGTACTGAGAGCTCTAGATGAAGACAACTTGATTGGTAGTGGTTCTTCCGGGAAGGTTT
ACAAGGTCGTTTTGAGTAATGGTGAGGCTGCTGCTGTGAAAAAACTTTCAAGAAATTCGAAA
AAAGTAGATGAGAGTTGTGACATCGAGAAAGGTAAGTATCAGGATGATGGATTTGATGCAG
AGGTTGAGACATTGGGCAAAATTCGACACAAGAACATCGTTAGGCTATGGTGTTGTTGTACA
ACAAGGGGTTGCAAACTTTTGGTTTATGAGTACATGCCTAATGGAAGCTTGGGTGATTTGCTA
CACAGCAGCAAAAGTGGGTTGTTGGATTGGCCTAAGAGATTTAAGATAGCTACGGATACTGC
AGAGGGACTCTCATATTTGCATCATGATTGTGCTCCTCCGATTGTTCACAGAGACTTTAAGTC
GAACAATATCTTGTTGGACGGGGAGTTTGGAGCTCGGGTAGCTGATTTTGGTGTGGCAAAGGT
GATTGATGTCGATGACAAGGGAACCATGTCTATGTCAGTCATTGCAGGGTCTTGCGGTTATAT
TGCTCCAGAATATGCATACACACTTCAGGTGAACGAGAAGAGTGATATATATAGTTTTGGCGT

GGTAGTCCTCGAGCTAGTGACAGGGAAACTCCCTGTAGGTCCCGAATACGGGGAAAAGGATT
TGGTGAAGTGGGTTTGCGCTACTCTAGACCAGAAGGGTATAAATCATGTTATTGACCCGAAA
CTCGACTCTTGTTTCAAGGAGGACATAAGCAAAGTCCTACAAATTGGCCTCCTCTGCACTAGC
CCCCTCCCAATCAACCGACCCCCGATGAGAAAAGTCGTAAAAATGCTGCAGGAAGTTGGTGG
CGGAGACCAGCTCAAGACAGCGTTAACAGATGGCAAGTTGACCCCTTACTACCACGAAGAC
GCATCAGATCAAGGAAATGTAGCTTAA

Gene Sequence:

>Solyc03g006300.1.1

ATGCATCTTCAAATCTTGCTTATACTTCTATTACCTACATTGATTCTCTCAATAAACCAAGAAT
CCCTTTATTTACATACCATAAAGCTTGGATTTGATGACCCAAATGGTGTTTTTTCAAACTGGAA
TCTCCATGATAACTCTTCACCCTGTAACTGGTATGGAGTAAAATGCGACTCTTTAACTCGTTCT
GTTACATCTATTGACCTCTCCAATACCAATATCGCCGGCCCATTTCCGGCTTCTCTTCTTTGCC
GGCTCAAGTATATTAAGTACATTTCATTCTATAATAACTCTATTAACTCGACACTTCCGGTGGA
GGAGTTATCTGCTTGTAAATCTCTTGTCCATCTCGATTTAGCTCAAAATTTGTTAGTGGGTAGT
CTTCCATCGAGTTTGGCTGAGCTTCACGAGCTGAAATATCTTGATTTAACCGGGAATAACTTT
ACCGGCGAAATTCCGGCGAGTTTTGGGGCTTTCCGGCGACTTGAAGTTCTGGGTTTGGTTGAA
AATTTGTTAACTGGGACTATCCCGCCGGAGATTGGAAATATTTCGAGTTTGAAACAGCTGAAT
TTGTCGTACAACCCGTTTTCGCCGGGTCGGGTCCCGCCGGAGATTGGGAACCTTACGAATCTC
GAGGTGCTTTGGTTAACTGACTGTGGGTTAATTGGTGAGGTTCCGGGTACATTAAGGGGATTA
AATAAGCTTGTTAACTTGGACCTTGCGTTAAACAACTTGTACGGTCCGATTCCGAGCTGGCTC
ACTGAGTTAACTAGTGTTGAGCAAATTGAGCTGTATAATAACTCGTTCTCCGGCGAGTTTCCG
GTGAATGGGTGGTCGAATATGACATCGTTGAGGCGGGTCGACGTGTCGATGAATCGGGTTAC
CGGGTCGATCCCGAACGGGTTGTGTGAGTTGCCACTTGAGTCGCTCAATCTTTATGAGAATCA
ATTGTATGGTGAGTTACCTGTAGCCATTGCAAATTCACCCAATTTATATGAATTAAAGCTCTTT
GGTAATAGTTTGAATGGAACTTTACCTGAAGATCTTGGTAAATTTTCGCCATTGGTATGGATTG
ATGTTTCAAACAATGAGTTTTCAGGTGAAATTCCGGTGAATTTGTGCGGAAATGGAGTCTTAG
AGGAGGTTTTGATGATAGATAACTCATTTTCCGGTGGAATTCCGCAGAGTTTAAGCCAATGCC
GGAGCTTATTACGTGTGAGGTTAGCTCATAATAAGTTCTCAGGTGATGTCCCTGTGGAATTTTG
GGGGCTGCCACGCCTCTCGCTGCTTGAGTTAACGAACAATTCATTTTCTGGTGGAATCGCGAA
AACTATAGCTGGTGCATCGAATTTATCAGCTTTGATTTTGTCAAAGAACGAATTTTCGGGTAA
TATTCCTGAAGAGATTGGCTTTTTGGAAAGTCTGGTTGATTTTGTGGGAAATGATAATAAGTTT
TCAGGGTCGTTGCCAGTTAGTATAGTGAATCTTGAGCAATTGGGAAGAATGGATTTCCACAAC
AATGAATTAAGTGGTAAGTTTCCTAGTGGGGTTCATTCTTTGAAGAAATTGAATGAATTGAAC
TTGGCAAACAATGATCTTTCTGGAGAAATTCCCCGAGAAATTGGGAGCTTGTCTGTTTTGAAC
TATCTTGACCTATCAGGAAACAAGTTTTCGGGGGAAATTCCAGTTGCGTTGCAGAATTTGAAG
CTCAATCAGCTGAATTTATCGAATAATGGCCTTTCGGGTGGTATTCCTCCTTCATATGCAAAG
GGAATGTACAAGAATAGCTTTCTGGGGAATCCAGGTTTATGTGGAGATATTGGAGGTTTATGT
GATGGAAAAGATGAAGGTAAAACTGCTGGTTATGTATGGTTACTGAGATTGCTTTTCGTACCT
GCTGTTTTGGTGTTTGTAGTTGGGGTAGTTTCGTTCTATTGGAAGTATAGGAATTACAAGAAAG
CAAAAAGGTTGGATAGATCGAAATGGACCTTGACGTCGTTTCATAAGTTAGATTTCAATGAGT
TTGAAGTACTGAGAGCTCTAGATGAAGACAACTTGATTGGTAGTGGTTCTTCCGGAAGGTTT
ACAAGGTCGTTTTGAGTAATGGTGAGGCTGCTGCTGTGAAAAAACTTTCAAGAAATTCGAAA
AAAGTAGATGAGAGTTGTGACATCGAGAAAGGTAAGTATCAGGATGATGGATTTGATGCAG
AGGTTGAGACATTGGGCAAAATTCGACACAAGAACATCGTTAGGCTATGGTGTTGTTGTACA

ACAAGGGGTTGCAAACTTTTGGTTTATGAGTACATGCCTAATGGAAGCTTGGGTGATTTGCTA
CACAGCAGCAAAAGTGGGTTGTTGGATTGGCCTAAGAGATTTAAGATAGCTACGGATACTGC
AGAGGGACTCTCATATTTGCATCATGATTGTGCTCCTCCGATTGTTCACAGAGACTTTAAGTC
GAACAATATCTTGTTGGACGGGGAGTTTGGAGCTCGGGTAGCTGATTTTGGTGTGGCAAAGGT
GATTGATGTCGATGACAAGGGAACCATGTCTATGTCAGTCATTGCAGGGTCTTGCGGTTATAT
TGCTCCAGGTTAGAGGATTGATCCTTCACTTTGTGAGTTTTACAAACATGATTTTATTGAAATT
GTTTTATGCCTTCCATTGCAAATTGTAGTTAAGATGTATGCCTAAATATAACATATGTCATATA
TTGTTAAGAGTATATTATAATTAAGCCTCGAATAAGGTCTGTTGTACACTCTATCCTCCTCTGA
CCTCGCTTGTGGGATTACATTGGGTATATTGTCGTTGATTATTGTTAAACCTCCAATATATATG
AGTAATTTTGGTCCATTGCTCGAATCCTTCAAAAAATGTGGCATGTCTTTGTTGGATTCTCCCA
AAGTTGATCAATTTTAGAGGATCCAATACAGGTGCAACGACATTTTGGAGGATCCGAGCAAC
TTAGAACTAACTTGATTGCCCTTGTACATTTAACACTAAGGTTTGCTCCAAGAACAGGTCATT
CTTTGTTGACATTTGCTTTCTGCTTCTCGTAGAGCTCCTAAGGTTGAACTAAATTGGGTTCGTCT
TGCAGAATATGCATACACACTTCAGGTGAACGAGAAGAGTGATATATATAGTTTTGGCGTGG
TAGTCCTCGAGCTAGTGACAGGGAAACTCCCTGTAGGTCCCGAATACGGGGAAAAGGATTTG
GTGAAGTGGGTTTGCGCTACTCTAGACCAGAAGGGTATAAATCATGTTATTGACCCGAAACT
CGACTCTTGTTTCAAGGAGGACATAAGCAAAGTCCTACAAATTGGCCTCCTCTGCACTAGCCC
CCTCCCAATCAACCGACCCCCGATGAGAAAAGTCGTAAAAATGCTGCAGGAAGTTGGTGGC
GGAGACCAGCTCAAGACAGCGTTAACAGATGGCAAGTTGACCCCTTACTACCACGAAGACG
CATCAGATCAAGGAAATGTAGCTTAA

5,-Upstream Sequence (1kb):

>Solyc03g006300.1.1

ATTCGATGTAAAGGAACTAGACTACGCAGAAGCACCATCATTCCCCAGTTGAACGGTAGACA
ATTGCGACAGAACTCGACCAGAGGCGGTGGTTGAAGGACGTCGTAAAAATGCTGAAAAGAG
TAGCCCCCAGCCAACTAACCCTCCCCCGATCACGTCTCCTCCGGTTAAACATCCTGAAACGA
ATACAGGAGGAACTTTGTTCTCAGCTCAAAGCCCAGTTATTGTACTAAATATGGGAAGACCA
GATCTCATCGCGTTTGGGTGAAGTGGTTTAGGAAAAGGGGCATAAGCCCTGGATGTCCCTCA
AAGGGACAGTGATCGAGCTCCTGATGGTGCGGTTTTGATATATATAGTGAGAAGAGCAAGTG
GACTTCACACATACGTATAAGACGTTCTGCTTGGGTTAAATCAAGTTGGAATCCTCGAGATGC
TCTTCGTCTTTCGTTTACAGTTGTTTCTTACTGGACAAGAACCTCGTTTGGAATCACAATTTAC
ATGTTCCCGTTAGTTCAATCAAGATTCAACGAGCCTAGGAGGTTTTACAGCAACGTGGACATA
ACCTAGGAGATTTTAACTAGTTGAAACCCTCTTAGGTTGTTTCTGTACGGTGTAAAAAACTTC
CTAAGCTCGTTACCTGGTTTTAATGAGTATATATAACCTCCAAATTGTTATTAGTTGCTGTTAT
ATGGGTTACATTAGGGTGTTCGCTCCAGTCTCCTCCTATCTCACATGTTGTCTGGAATAAGCTC
CGAATTAATATTATATGAGAATTGTTATATACTGTATACAATATAAATCCGTATGTAGAATTG
ATGTTAAACGTTACCTTCCGTATTTTGTTAAAGTTATTTTAGTACAAACATTTTGAGTGTTTCAC
TTCCTAGTTAGGAGATTGGACCTCGTTATATTGGCGTTCTGGGACGTTACTGACTGTATCTGTA
CCAAGGGAACAGTAGCTGTAGTTAGTGGAAACGGTGTGGTTTTAGTCGATGGGCTC

>SlHSL4 ORF Solyc04g077010.2.1

Protein Sequence:

>Solyc04g077010.2.1

MTIQKMITLVLFSFTLSTFVQSDQSQFFLLMKKFVTGSSLSNWDIEKPICQYRGVGCDDRGNVIKINIS
AWYLSGQFPSDVCSYLPRLKSLHIGHNNFQGGFPKYLINCSLLEELNMTKTSLTGQIPDLSPLKSLRV

LDLSCNKLTGDFPLSILNLTNLVILNFNENRHFNPWRLPEEISRLINLKWMILTACNMHGTIPVTISN
MTSLVDLELSANRLAGKVPKELGKLKNLRLLELFYNLLDGEIPAELGNLTELVDLDMSANNFTGRIP
ESISRLPKLEVLQLYHNALSGEFPAALANSTTLTILSLYDNLFTGEVPQHFGLSSALLALDLSENRFSG
KLPPFLCSGELFMQSNKLSGLLPYEISTSSNLVKLDLSNNLLYGPIPSEIGGLKSLNLLLLQGNKFNSSI
PESLSSLKSLNYLDLSSNLLIGKIPESLGELLPNSMNLSNNLLSGPIPLLFIKGGVLESFSGNPGLCVPTS
LNSSDRSFQTCSHSYNHKKRNNIAWVIGTSVGIVIVGLVLFIKRWFGNKKAVMEQDDHSLSSSFFSY
DVKSFHRLSFDQREIFEAMVEKNIVGYGGSGAVYKIELSNGGVVAAKKLWSHKHKHSVSEDQLVL
DKELKTEVETLGNIRHKNIVKLYCYFSSLDCSLLVYEYMPNGNLWHALHGGKFVLDWPIRHQIALG
IAQGLAYLHHDLMPPIIHRDIKSTNILLDIDYQPKVADFGIAKVLQARGGKDSSTTVIAGTYGYLAPE
YAYSSKATTKCDVYSFGVVLMELITGKKPVEPEFGDNKNIVYWVSTKVETKEGAFEVLDKKVSDSFK
EDMIKVLRIAIRCTYSTPTLRPTMNEVVQLLIEADPCKFNCCNMSNKKKSDTEEVINKPPKSIYDL

CDS Sequence:

>Solyc04g077010.2.1

ATGACTATTCAAAAAATGATCACCCTTGTGTTGTTTTCTTTTACTTTGTCCACTTTCGTTCAATC
TGATCAATCGCAGTTCTTTCTACTCATGAAGAAATTCGTCACAGGGAGTTCTTTGTCTAATTGG
GATATCGAAAAACCGATTTGTCAATACAGAGGTGTTGGTTGTGATGACAGGGGAAATGTTAT
CAAAATTAATATTTCAGCCTGGTATTTATCAGGCCAATTTCCAAGTGATGTGTGTAGTTATTTA
CCAAGATTGAAAAGCCTTCATATTGGTCATAATAATTTCCAAGGTGGTTTTCCTAAGTACTTA
ATAAATTGTTCTCTTTTGGAAGAATTGAACATGACTAAAACATCACTTACAGGACAAATCCCT
GATTTGTCACCATTGAAATCATTGAGAGTACTTGATCTTTCATGTAATAAATTGACAGGGGAT
TTTCCTTTGTCAATTCTTAATCTCACAAACTTAGTTATCCTGAATTTCAACGAAAATCGCCATT
TTAATCCGTGGCGATTACCAGAGGAAATTTCCAGGCTGATAAATCTCAAGTGGATGATTTTAA
CAGCTTGTAATATGCATGGGACAATTCCAGTGACGATAAGTAACATGACATCTCTTGTTGATC
TTGAATTGAGCGCGAATCGTCTTGCTGGTAAGGTGCCTAAAGAACTTGGGAAGTTGAAAAAT
TTGAGACTCCTTGAGCTTTTCTACAATCTACTTGATGGTGAAATCCCCGCGGAGCTAGGGAAT
TTGACAGAACTTGTGGACTTAGACATGTCCGCGAATAATTTCACAGGGAGAATTCCAGAGTC
TATAAGCCGGCTTCCTAAGCTGGAAGTATTGCAGCTTTACCATAACGCGCTATCAGGAGAGTT
TCCAGCAGCACTTGCTAATTCAACAACTTTAACTATCCTGTCTCTTTATGACAATCTGTTTACA
GGAGAAGTTCCACAACACTTTGGTCTTTCATCAGCTTTGTTAGCATTGGACTTGTCAGAAAAT
CGATTTTCTGGGAAGCTACCGCCTTTTCTGTGTAGTGGAGAATTGTTTATGCAAAGTAACAAG
CTTTCAGGTTTGCTTCCTTATGAAATATCTACATCTTCTAATCTTGTGAAGCTTGATCTTAGCAA
TAACCTTTTGTATGGTCCAATTCCTTCTGAAATTGGTGGTTTAAAAAGCCTCAATTTGTTACTC
TTGCAAGGTAACAAGTTCAATTCTTCAATCCCCGAGTCACTTTCTTCGCTTAAATCTCTCAACT
ATCTTGATCTGTCTAGTAATCTTTTGATAGGAAAGATCCCTGAGAGCCTAGGTGAATTGTTAC
CAAACTCTATGAACTTGTCAAACAACTTACTTTCCGGTCCTATACCTCTTTTGTTCATAAAAGG
AGGTGTTTTGGAAAGTTTTTCAGGCAATCCGGGACTCTGTGTTCCTACCTCTTTGAACTCATCA
GACAGAAGTTTTCAAACATGTTCACATAGCTATAACCATAAGAAGAGGAACAACATTGCTTG
GGTTATTGGGACATCAGTGGGGATAGTCATTGTTGGATTAGTTTTGTTTATCAAGCGATGGTTT
GGTAACAAAAAGGCGGTGATGGAACAGGATGATCATTCATTGTCATCATCATTTTTCTCCTAT
GATGTTAAGAGTTTCCATCGATTGAGCTTTGATCAACGTGAGATCTTTGAAGCTATGGTTGAG
AAGAACATTGTTGGATATGGAGGGTCAGGAGCTGTTTATAAGATCGAGTTGAGTAACGGAGG
AGTAGTTGCTGCAAAGAAGCTGTGGAGTCACAAACATAAGCATTCTGTTTCTGAGGATCAATT
GGTTTTGGATAAGGAACTTAAGACAGAAGTTGAGACTCTTGGTAACATAAGGCACAAAAAC
ATTGTGAAATTGTACTGTTATTTCTCAAGTTTGGATTGCAGCTTGTTGGTTTATGAATACATGC

CTAATGGAAACCTTTGGCATGCTCTTCATGGTGGGAAGTTTGTGTTGGATTGGCCTATTCGTCA
TCAAATAGCACTCGGAATAGCTCAAGGATTGGCCTATCTTCACCATGATCTTATGCCACCGAT
TATTCATAGAGATATCAAATCCACCAACATCCTCCTGGATATTGATTATCAGCCAAAAGTCGC
GGATTTTGGTATAGCCAAAGTGTTGCAAGCCAGAGGAGGCAAAGATTCCAGCACCACAGTCA
TCGCAGGAACCTATGGTTACTTGGCACCAGAGTATGCATATTCTTCAAAGGCAACTACAAAG
TGTGATGTGTATAGTTTTGGAGTTGTTCTAATGGAATTGATTACTGGAAAGAAGCCAGTGGAG
CCAGAATTTGGAGACAACAAGAACATTGTTTATTGGGTATCAACAAAAGTGGAGACTAAAG
AAGGTGCATTTGAAGTATTGGATAAAAAAGTCTCAGATTCTTTCAAAGAGGACATGATTAAG
GTTCTACGTATAGCTATACGTTGTACGTATAGTACACCAACACTTCGTCCTACCATGAATGAA
GTCGTTCAGCTGCTAATCGAGGCGGATCCTTGCAAGTTCAATTGTTGCAACATGTCAAATAAG
AAGAAGAGTGACACAGAAGAAGTGATCAATAAGCCACCAAAGAGCATATATGATTTGTAA

Gene Sequence:

>Solyc04g077010.2.1

CACAACCAAAATATAACAAAACTTTTATTTCCTTCCTCTCTAAGAAAAAACAAATTTAATACA
AATGACTATTCAAAAAATGATCACCCTTGTGTTGTTTTCTTTTACTTTGTCCACTTTCGTTCAAT
CTGATCAATCGCAGTTCTTTCTACTCATGAAGAAATTCGTCACAGGGAGTTCTTTGTCTAATTG
GGATATCGAAAAACCGATTTGTCAATACAGAGGTGTTGGTTGTGATGACAGGGGAAATGTTA
TCAAAATTAATATTTCAGCCTGGTATTTATCAGGCCAATTTCCAAGTGATGTGTGTAGTTATTT
ACCAAGATTGAAAAGCCTTCATATTGGTCATAATAATTTCCAAGGTGGTTTTCCTAAGTACTT
AATAAATTGTTCTCTTTTGGAAGAATTGAACATGACTAAAACATCACTTACAGGACAAATCCC
TGATTTGTCACCATTGAAATCATTGAGAGTACTTGATCTTTCATGTAATAAATTGACAGGGGA
TTTTCCTTTGTCAATTCTTAATCTCACAAACTTAGTTATCCTGAATTTCAACGAAAATCGCCAT
TTTAATCCGTGGCGATTACCAGAGGAAATTTCCAGGCTGATAAATCTCAAGTGGATGATTTTA
ACAGCTTGTAATATGCATGGGACAATTCCAGTGACGATAAGTAACATGACATCTCTTGTTGAT
CTTGAATTGAGCGCGAATCGTCTTGCTGGTAAGGTGCCTAAAGAACTTGGGAAGTTGAAAAA
TTTGAGACTCCTTGAGCTTTTCTACAATCTACTTGATGGTGAAATCCCCGCGGAGCTAGGGAA
TTTGACAGAACTTGTGGACTTAGACATGTCCGCGAATAATTTCACAGGGAGAATTCCAGAGT
CTATAAGCCGGCTTCCTAAGCTGGAAGTATTGCAGCTTTACCATAACGCGCTATCAGGAGAGT
TTCCAGCAGCACTTGCTAATTCAACAACTTTAACTATCCTGTCTCTTTATGACAATCTGTTTAC
AGGAGAAGTTCCACAACACTTTGGTCTTTCATCAGCTTTGTTAGCATTGGACTTGTCAGAAAA
TCGATTTTCTGGGAAGCTACCGCCTTTTCTGTGTAGTGGAGGTAAATTGAGTTACATTCTTTTA
CTTCAAAACATGTTCTCAGGTGAACTGCCTGATGGATATGTGAAATGTCAGTCTGTTCTTCGCT
TTCGAGTGAATTACAATCAGTTAGAGGGAAGTATACCACAAGAGCTTTTTACTCTTCCACATG
TTTCGATTATTGATTTGAGCTATAATCATTTTAGTGGTTCAATTCCAACAACAATTGGAAGTGC
TAGGAATTTATCAGAATTGTTTATGCAAAGTAACAAGCTTTCAGGTTTGCTTCCTTATGAAATA
TCTACATCTTCTAATCTTGTGAAGCTTGATCTTAGCAATAACCTTTTGTATGGTCCAATTCCTTC
TGAAATTGGTGGTTTAAAAAGCCTCAATTTGTTACTCTTGCAAGGTAACAAGTTCAATTCTTC
AATCCCCGAGTCACTTTCTTCGCTTAAATCTCTCAACTATCTTGATCTGTCTAGTAATCTTTTGA
TAGGAAAGATCCCTGAGAGCCTAGGTGAATTGTTACCAAACTCTATGAACTTGTCAAACAAC
TTACTTTCCGGTCCTATACCTCTTTTGTTCATAAAAGGAGGTGTTTTGGAAAGTTTTTCAGGCA
ATCCGGGACTCTGTGTTCCTACCTCTTTGAACTCATCAGACAGAAGTTTTCAAACATGTTCAC
ATAGCTATAACCATAAGAAGAGGAACAACATTGCTTGGGTTATTGGGACATCAGTGGGGATA
GTCATTGTTGGATTAGTTTTGTTTATCAAGCGATGGTTTGGTAACAAAAAGGCGGTGATGGAA
CAGGATGATCATTCATTGTCATCATCATTTTTCTCCTATGATGTTAAGAGTTTCCATCGATTGA

GCTTTGATCAACGTGAGATCTTTGAAGCTATGGTTGAGAAGAACATTGTTGGATATGGAGGGT
CAGGAGCTGTTTATAAGATCGAGTTGAGTAACGGAGGAGTAGTTGCTGCAAAGAAGCTGTGG
AGTCACAAACATAAGCATTCTGTTTCTGAGGATCAATTGGTTTTGGATAAGGAACTTAAGACA
GAAGTTGAGACTCTTGGTAACATAAGGCACAAAAACATTGTGAAATTGTACTGTTATTTCTCA
AGTTTGGATTGCAGCTTGTTGGTTTATGAATACATGCCTAATGGAAACCTTTGGCATGCTCTTC
ATGGTGGGAAGTTTGTGTTGGATTGGCCTATTCGTCATCAAATAGCACTCGGAATAGCTCAAG
GATTGGCCTATCTTCACCATGATCTTATGCCACCGATTATTCATAGAGATATCAAATCCACCA
ACATCCTCCTGGATATTGATTATCAGCCAAAAGTCGCGGATTTTGGTATAGCCAAAGTGTTGC
AAGCCAGAGGAGGCAAAGATTCCAGCACCACAGTCATCGCAGGAACCTATGGTTACTTGGC
ACCAGGTATAATTTTTTCAATTCATAAGTGTTACTTCAATCGTTGAGCATGATTAAGTAGATTA
AATAACATCCTATAGTTTTTAGTGTTCATATTGGCCTAATGTTACTCTAAATATTAGAACTTTC
CGTAACGTTTAAGAGTATATTTGACCCTTTTCCGTTCTTAATTATGACTTTTTATGTGTAAAAA
ACATCACATATACTCAAGTTAGTTGATTAAACTTTCATTTAACACATGTATAAGTGTTAAAATT
GGGCTAATGTCACTAAACCTTTGGTGTAACAGAGTATGCATATTCTTCAAAGGCAACTACAA
AGTGTGATGTGTATAGTTTTGGAGTTGTTCTAATGGAATTGATTACTGGAAAGAAGCCAGTGG
AGCCAGAATTTGGAGACAACAAGAACATTGTTTATTGGGTATCAACAAAAGTGGAGACTAA
AGAAGGTGCATTTGAAGTATTGGATAAAAAAGTCTCAGATTCTTTCAAAGAGGACATGATTA
AGGTTCTACGTATAGCTATACGTTGTACGTATAGTACACCAACACTTCGTCCTACCATGAATG
AAGTCGTTCAGCTGCTAATCGAGGCGGATCCTTGCAAGTTCAATTGTTGCAACATGTCAAATA
AGAAGAAGAGTGACACAGAAGAAGTGATCAATAAGCCACCAAAGAGCATATATGATTTGTA
AAAAATTTAGTATATCAAATTAGTATACAAGGTATATAGAATATATGAGTACTGAGATATTA
CACCTTTAAAAGAATTTTAGTCTATATGTTTTCTAATAAATGGTGTACTCTTTATTAGCATGTA
GATTTTTCAATTAGTGTTACTTTGCTTTTTGTATTTTTGGCCTGTTGTAAGTGTTGGGAATTTTAT
GATATACAACAGCTTTATTGTAACGCATAGAATATTTGTATTATGGAGTTCTTGTTGG

5,-Upstream Sequence (1kb):

>Solyc04g077010.2.1.1
NNNNNNNNNNNNNNNNNNNNNNNNNNNNNNNNNNNNNNNNNNNNNNNNNNNNNNNNNNNN
NNNNNNNNNNNNNNNNNNNNNNNNNNNNNNNNNNNNNNNNNNNNNNNNNNNNNNNNNNNN
NNNNNNNNNNNNNNNNNNNNNNNNNNNNNNNNNNNNNNNNNNNNNNNNNNNNNNNNNNNN
NNNNNNNNNNNNNNNNNNNNNNNNNNNNNNNNNNNNNNNNNNNNNNNNNNNNNNNNNNNN
NNNNNNNNNNNNNNNNNNNNNNNNNNNNNNNNNNNNNNNNNNNNNNNNNNNNNNNNNNNN
NNNNNNNNNNNNNNGTATTGTACGGTAGATACAATGTTTGGCTAGATTGTTTATTTGTTGTT
GTTTAGTAACATTTTAATTATTTGGTTTGATCGTATCGTACTGTATTATAATTTATAAATTTACT
TGAATATCCTTAATTATTCTAGGGTAGAGGTTTGACTAAATTTAAATAATTAAGGTAAAGAGT
AAAATAGTATTTTGAAATATTATGTAAAGATATAATTGAAAAAAAGAAATTAAGTAACAATG
AGAACACACCAAATTAGTTGTTCCATAAAATAGGGATTTTCATTGTTATGTAACAACAACAC
AGTACAATATATTTTAAGTAACAATTAAAACAAACATTGTAGGTATAGTAACAATACAATAT
AATGGATAACAATGATTTAAACATAATATAAAAAAATTCAAATTTATTACAACATAATCTAC
CTAAGCCAATAGTATTCATATAAAATTGCTACTGATATATAAGAATATGATCAAAGTAACAA
AATCTTGATCAATAAATCCATGCAAAAACATATATAAATGAGATACAGTATATTTGAACAGC
TCTTCATGAGTATAAAGGGCTTTATATATATTCTCTTTAGGGGTTGCAAGTGGAGGGTATAAA
CATAAAGCTTCTTTCTTTTCTATCTCTCACTTTCAATACATCAAAGCACCAAAATGTTCCTATC
AATCTTCCCATTTATATATTTCTTCACCACAATCTCCTCTTC

>SlHSL5 ORF Solyc02g091860.2.1

Protein Sequence:

>Solyc02g091860.2.1

MDYMKLQLLILISFFLFIVPASSSPRDIAILLRVKSAQLDDPNGLIADWNGSAPNAPCSWNGIKCDRR
TGQVLSIDFGSFGIAGRFPADFCRISTLQELNLGDNSFGESISSDSWSLCSHLHLLNISLNFFVGRLPEF
VTKFDNLTVLDANSNNFSGEIPASLGRLPKLQVLNIANNLLNGSIPEFLTNLTELTRLEIAANPFKPG
PLPSSIGRLGKLRIFYARFASLVGNFPDSIKDLKSIQDFDVANNNLSGKIPESFGKLKTIQQIELFGNHF
SGELPDMFSGLGSLSRFDASENNLTGKIPETLTHLPLESLNLNDNQLEGEISENLALNPNLSQLKLFN
NRFSGTLPQTFGLSSDLDEFDVSGNNLEGSLPPNLCSRKKLRILNLFDNKFNGPIPESYGQCYSLSYV
RIYNNQFSGELPTGFWGFDGYTFLELRNNNFQGSIPASISNARGLTQILISGNNFSGELPAEICNLEEV
VFMDISKNQLSGQLPSCITRLKKLQKLDLSQNRIRGQIPKSVSSWNELTELSLADNQLTGEIPGELGM
LPVLTYLDLASNLLSGEIPSELSKLKLNKFNVSNNRLEGKVPLGFDNDFFVSGLLGNPDLCSPDLKPL
PQCRRPKSVSLYLVCILSAFAFILVGSLVCVLLKASKLLPIRSKRKSVWRITAFQRVGFTERDVLDALIE
KNLIGAGGSGRVYRVKLKNGQMVAVKKLWAAKRERESEEVFRSEVETLGRVRHGNIVKLLYTGIG
DDFRILVYEYMENGSLGDVLHGEKGGLLLDWPRRFAIAVGAAHGLAYLHHDSVPAVVHRDVKSN
NILLDEDFRPKVADFGLAKAMRGDAEESDQAMSHIAGSYGYIAPEYAYTLKITEKSDVYSFGVVLLE
LIIGKRPNDSSFGEDKDVVKWVLEVATSSKKDEGTGHIVTCAGGILDLNQLVDQRMNPSASDYAEI
KNVLDVALLCTSALPINRPSMRRVVELLKNIPSARSKTTH

CDS Sequence:

>Solyc02g091860.2.1

ATGGATTACATGAAGCTTCAATTGCTGATACTCATAAGTTTTTTTCTCTTCATTGTTCCGGCGA
GTTCATCGCCTCGGGATATTGCTATTTTACTCCGGGTTAAGTCCGCCCAACTCGATGACCCGA
ATGGGTTGATTGCTGATTGGAACGGGTCTGCTCCAAATGCGCCTTGCAGCTGGAACGGGATC
AAGTGTGATCGTAGAACCGGTCAGGTTCTGTCCATTGATTTTGGGAGTTTTGGAATCGCAGGT
CGTTTTCCTGCTGATTTCTGCCGGATTTCGACTTTGCAGGAACTCAATCTGGGTGATAACAGTT
TTGGTGAGTCCATTTCCTCTGACTCATGGTCACTCTGTTCGCATCTACACTTATTGAATATTTCT
TTAAATTTCTTTGTTGGCCGGCTGCCGGAGTTTGTTACCAAGTTTGATAACTTGACCGTCCTTG
ATGCTAATTCAAACAATTTCTCCGGTGAAATCCCGGCGAGTTTAGGCCGTTTACCCAAATTAC
AAGTGCTAAATATAGCTAACAATCTCCTCAATGGTTCAATTCCTGAGTTCTTGACGAATCTTA
CCGAGTTGACTCGATTGGAAATTGCTGCAAATCCGTTTAAGCCAGGTCCATTGCCTTCCTCAA
TCGGCCGACTCGGTAAGCTTCGAATTTTCTATGCTCGGTTTGCGAGCCTTGTTGGGAATTTTCC
AGATTCTATCAAAGACCTGAAATCTATTCAGGATTTTGATGTGGCAAACAACAATCTCTCCGG
AAAAATTCCAGAAAGCTTCGGAAAACTCAAAACCATACAACAAATAGAGCTCTTTGGGAAC
CATTTCTCAGGCGAATTGCCGGACATGTTTTCCGGTCTTGGTTCTCTTTCCAGGTTTGACGCTTC
TGAGAACAATCTCACCGGGAAAATACCTGAAACCCTTACCCATTTGCCATTGGAATCTTTAAA
TCTCAATGATAACCAATTAGAAGGCGAAATTTCAGAAAATTTAGCTCTTAACCCAAATCTTAG
TCAGTTAAAGCTTTTTAACAACAGATTTTCAGGTACTTTACCTCAAACGTTTGGTTTAAGTTCA
GATTTAGATGAGTTTGATGTCTCCGGCAACAATCTAGAAGGTTCTTTACCTCCCAACCTATGTT
CTAGAAAGAAACTTAGGATTTTGAACCTGTTTGATAATAAGTTCAATGGGCCAATCCCAGAA
TCCTATGGGCAGTGTTATTCACTATCATATGTGCGTATCTATAACAATCAATTCTCTGGTGAAT
TACCAACTGGTTTCTGGGGATTTGATGGATACACATTTCTTGAACTGCGAAACAACAACTTTC
AAGGTTCAATTCCAGCTTCAATCTCCAATGCTCGAGGCCTGACACAAATTCTCATCTCTGGCA
ACAACTTCTCCGGAGAATTGCCAGCAGAAATATGCAATTTGGAAGAGGTTGTGTTCATGGAC
ATTAGCAAGAATCAATTATCAGGGCAGTTGCCTTCGTGTATCACAAGATTGAAAAAGTTACA

AAAGCTTGATCTTTCACAAAATAGGATCAGGGGTCAAATTCCCAAATCAGTTAGTTCTTGGAA
TGAATTGACTGAGTTGAGTTTAGCTGACAATCAACTGACCGGTGAAATTCCTGGTGAGCTTGG
GATGTTACCGGTCTTAACATACTTAGACCTCGCCTCAAACTTGCTTTCTGGTGAAATTCCATCC
GAGCTGAGCAAGCTCAAGCTCAACAAATTCAATGTATCGAATAACAGGCTGGAAGGGAAAG
TGCCACTTGGGTTTGATAACGATTTTTTCGTCTCAGGTTTACTGGGCAATCCGGATCTTTGTAG
TCCAGATCTTAAGCCTCTGCCCCAGTGCCGAAGACCTAAAAGTGTAAGCTTGTACTTGGTGTG
TATTTTATCAGCTTTTGCCTTCATACTTGTTGGGTCACTTGTTTGTGTCTTACTCAAGGCCAGTA
AGCTGCTACCAATCCGTAGCAAACGTAAAAGTGTGTGGAGAATTACTGCATTCCAACGTGTC
GGTTTCACAGAGAGAGACGTGTTAGATGCACTGATAGAAAAAAATCTCATTGGAGCTGGTGG
GTCGGGTCGGGTGTACCGGGTCAAATTGAAAAACGGGCAGATGGTTGCGGTGAAGAAACTTT
GGGCGGCTAAACGGGAAAGAGAATCCGAGGAGGTGTTCAGGTCAGAGGTGGAGACATTAGG
GAGAGTTCGGCATGGAAACATAGTGAAACTATTGTACACTGGCATTGGTGATGACTTTAGGA
TATTGGTGTACGAATACATGGAGAATGGAAGCTTAGGAGACGTATTACATGGGGAAAAAGGT
GGCTTGTTATTGGATTGGCCGAGGAGATTTGCCATAGCAGTTGGAGCAGCTCATGGATTGGCC
TATTTGCACCATGATTCTGTGCCAGCAGTAGTTCACAGAGATGTTAAGTCTAATAACATTTTGT
TGGACGAAGATTTCAGGCCCAAAGTGGCTGATTTTGGGCTAGCCAAGGCAATGCGAGGGGAT
GCTGAGGAGAGTGATCAAGCCATGTCCCACATTGCTGGTTCCTACGGCTACATTGCACCTGAA
TATGCCTACACTCTGAAGATCACTGAGAAGAGTGATGTTTATAGCTTTGGTGTGGTACTGTTG
GAACTAATAATTGGTAAAAGGCCTAATGACTCCTCTTTCGGAGAGGATAAGGACGTTGTCAA
GTGGGTGTTAGAGGTTGCAACATCGTCTAAGAAAGACGAAGGAACTGGCCATATTGTTACGT
GCGCAGGTGGTATTCTTGATTTGAATCAGCTAGTTGACCAGAGAATGAATCCATCTGCAAGCG
ATTACGCAGAGATTAAAAATGTTTTGGATGTGGCTTTGCTTTGCACTTCAGCATTGCCTATCAA
TAGGCCTTCCATGAGAAGAGTTGTTGAATTGCTGAAGAATATCCCCTCCGCTCGTTCTAAAAC
AACGCATTAG

Gene Sequence:

>Solyc02g091860.2.1

TGAGTCCAAAAACCTTATCATTACTCTCTGCTTCAAGTTACTGTAACTCTTGTCTTGTTCGTCG
CCATTAGAGTCCAATACTAGCTTTTCTATCTAATGTGTGAACACTCTTCACGCTTTCAGTACTA
TTTCTTCTCCTTAAGCTCTGCTTCCGTTACACTTGTAATCCATGCATAAATTTCCTCCACCTCTT
TCTAACTCTTATCTTCCCAACATTCAACCTCCATTTACACATGTTTATGATTAATCGGTTCTTGC
CTTTTTAAGGACTTGAGGAGCAGGGGAGATTCATTCACTCATCAATGGATTACATGAAGCTTC
AATTGCTGATACTCATAAGTTTTTTTCTCTTCATTGTTCCGGCGAGTTCATCGCCTCGGGATATT
GCTATTTTACTCCGGGTTAAGTCCGCCCAACTCGATGACCCGAATGGGTTGATTGCTGATTGG
AACGGGTCTGCTCCAAATGCGCCTTGCAGCTGGAACGGGATCAAGTGTGATCGTAGAACCGG
TCAGGTTCTGTCCATTGATTTTGGGAGTTTTGGAATCGCAGGTCGTTTTCCTGCTGATTTCTGCC
GGATTTCGACTTTGCAGGAACTCAATCTGGGTGATAACAGTTTTGGTGAGTCCATTTCCTCTGA
CTCATGGTCACTCTGTTCGCATCTACACTTATTGAATATTTCTTTAAATTTCTTTGTTGGCCGGC
TGCCGGAGTTTGTTACCAAGTTTGATAACTTGACCGTCCTTGATGCTAATTCAAACAATTTCTC
CGGTGAAATCCCGGCGAGTTTAGGCCGTTTACCCAAATTACAAGTGCTAAATATAGCTAACA
ATCTCCTCAATGGTTCAATTCCTGAGTTCTTGACGAATCTTACCGAGTTGACTCGATTGGAAAT
TGCTGCAAATCCGTTTAAGCCAGGTCCATTGCCTTCCTCAATCGGCCGACTCGGTAAGCTTCG
AATTTTCTATGCTCGGTTTGCGAGCCTTGTTGGGAATTTTCCAGATTCTATCAAAGACCTGAAA
TCTATTCAGGATTTTGATGTGGCAAACAACAATCTCTCCGGAAAAATTCCAGAAAGCTTCGGA
AAACTCAAAACCATACAACAAATAGAGCTCTTTGGGAACCATTTCTCAGGCGAATTGCCGGA

CATGTTTTCCGGTCTTGGTTCTCTTTCCAGGTTTGACGCTTCTGAGAACAATCTCACCGGGAAA
ATACCTGAAACCCTTACCCATTTGCCATTGGAATCTTTAAATCTCAATGATAACCAATTAGAA
GGCGAAATTTCAGAAAATTTAGCTCTTAACCCAAATCTTAGTCAGTTAAAGCTTTTTAACAAC
AGATTTTCAGGTACTTTACCTCAAACGTTTGGTTTAAGTTCAGATTTAGATGAGTTTGATGTCT
CCGGCAACAATCTAGAAGGTTCTTTACCTCCCAACCTATGTTCTAGAAAGAAACTTAGGATTT
TGAACCTGTTTGATAATAAGTTCAATGGGCCAATCCCAGAATCCTATGGGCAGTGTTATTCAC
TATCATATGTGCGTATCTATAACAATCAATTCTCTGGTGAATTACCAACTGGTTTCTGGGGATT
TGATGGATACACATTTCTTGAACTGCGAAACAACAACTTTCAAGGTTCAATTCCAGCTTCAAT
CTCCAATGCTCGAGGCCTGACACAAATTCTCATCTCTGGCAACAACTTCTCCGGAGAATTGCC
AGCAGAAATATGCAATTTGGAAGAGGTTGTGTTCATGGACATTAGCAAGAATCAATTATCAG
GGCAGTTGCCTTCGTGTATCACAAGATTGAAAAAGTTACAAAAGCTTGATCTTTCACAAAATA
GGATCAGGGGTCAAATTCCCAAATCAGTTAGTTCTTGGAATGAATTGACTGAGTTGAGTTTAG
CTGACAATCAACTGACCGGTGAAATTCCTGGTGAGCTTGGGATGTTACCGGTCTTAACATACT
TAGACCTCGCCTCAAACTTGCTTTCTGGTGAAATTCCATCCGAGCTGAGCAAGCTCAAGCTCA
ACAAATTCAATGTATCGAATAACAGGCTGGAAGGGAAAGTGCCACTTGGGTTTGATAACGAT
TTTTTCGTCTCAGGTTTACTGGGCAATCCGGATCTTTGTAGTCCAGATCTTAAGCCTCTGCCCC
AGTGCCGAAGACCTAAAAGTGTAAGCTTGTACTTGGTGTGTATTTTATCAGCTTTTGCCTTCAT
ACTTGTTGGGTCACTTGTTTGTGTCTTACTCAAGGCCAGTAAGCTGCTACCAATCCGTAGCAA
ACGTAAAAGTGTGTGGAGAATTACTGCATTCCAACGTGTCGGTTTCACAGAGAGAGACGTGT
TAGATGCACTGATAGAAAAAAATCTCATTGGAGCTGGTGGGTCGGGTCGGGTGTACCGGGTC
AAATTGAAAAACGGGCAGATGGTTGCGGTGAAGAAACTTTGGGCGGCTAAACGGGAAAGAG
AATCCGAGGAGGTGTTCAGGTCAGAGGTGGAGACATTAGGGAGAGTTCGGCATGGAAACAT
AGTGAAACTATTGTACACTGGCATTGGTGATGACTTTAGGATATTGGTGTACGAATACATGGA
GAATGGAAGCTTAGGAGACGTATTACATGGGGAAAAAGGTGGCTTGTTATTGGATTGGCCGA
GGAGATTTGCCATAGCAGTTGGAGCAGCTCATGGATTGGCCTATTTGCACCATGATTCTGTGC
CAGCAGTAGTTCACAGAGATGTTAAGTCTAATAACATTTTGTTGGACGAAGATTTCAGGCCCA
AAGTGGCTGATTTTGGGCTAGCCAAGGCAATGCGAGGGGATGCTGAGGAGAGTGATCAAGC
CATGTCCCACATTGCTGGTTCCTACGGCTACATTGCACCTGGTAAGCTTAAATTCCTTATTGTT
TTTCACCATTCATTTTATGTCCAATGAATGTTAGAAATTTTTCGTTCTGTAAGAAATTTATTTGC
CAATAACAGATGTTGAAAACATATAGTGCTAAGGACACTATCTAAGAAAAAAAAATATTGA
ACCGAAATAGGCATAAATGAAGTGGTATGGACAGCAGGAATTCGTAAAGTTAACCTCTACTT
GTTTGGGACCGTGGCCTAGTTGTTGTTGGTTTCAACTTTCTTGTTAAGTTGCTGTGAAGAGATA
TTCAATATCTTGCGTTTGGCATATCAAGGACATCCAAAAATAGTTTTCTGGAAGTTTTGGTTAA
CTTTGCATACTATCTTAAAATTTTGATTTGATTTGATTTGAGATTCTTTTGTATGAAGCCAGATA
AGATTTTAGGAGATGTTTCAAACTTATTAGTAGATTGGAAACCTTAATATTCTCCATTTAATGG
TAGCATCTAGTTTCATTCCTCTAACTGGACGCTGTTCTGAAGTCTGCATATTGTTTTTTTGCTTG
CTAATGCTTGATTATATGATTGGCAAGGGGATTTGTCAATGTAAAACCAAAGAATGATTGGG
CACCCCATTTTTGAAGTCTGCATGACTGTTTAGTTTTGACTACCTGAAATATTTAATCCTGAGT
TTCATTTGGGGTTTGTCAAAGTACAATCTAAGCATAGTTGGGCACTCCGTTTTCTAAAGTCTGC
ATGACCATTTATTCTACACCAGTAGTAATATTTAATTCTGAGATCACATGGAATTATTGAATG
ATTTTGTCACCTAATGTTTGTTAGCACAATCAAGATTTGATTGAAAGAATAAAAAATAGGAGG
GTCATGTGTGTCCTGTCACCTAGCATTAGTTAACGTAATTCAAGAATTGGTCTAAAAGAATAG
GATTGAAAGAACATGTGCCTAAGTGTCGTTTCTTATTTATCTAATTTATCGATGTCTTAAATTC
TATTTACCACCATAAGTCCTCTTTGATTGGACATACAATTTCATCTTTTTTGTAAAGCTGACAC

TATCCGGTCTGTGACTGCCCGTGTGCCCTTCCTTTGCAAAAGTGAGAAGAATACAGAAACCA
CGCCCTTGATGGAAAACTTTTGATGGCCTTTTGATACTAGAAAACATTCCTCTTGCAATTGCA
AACAGTTTGTTAAGTTGATCTTGGCTACTTAATGATTAGGAAGATGCTAAAGTTTGCTAAATA
TCTCATGGGACGGCAAATGTGTTATCCAAGGTTTTCAGCTGCGCGTCTAAACTTAACCTCAAT
TCCTTTTGTTTCAATTGCAGAATATGCCTACACTCTGAAGATCACTGAGAAGAGTGATGTTTAT
AGCTTTGGTGTGGTACTGTTGGAACTAATAATTGGTAAAAGGCCTAATGACTCCTCTTTCGGA
GAGGATAAGGACGTTGTCAAGTGGGTGTTAGAGGTTGCAACATCGTCTAAGAAAGACGAAG
GAACTGGCCATATTGTTACGTGCGCAGGTGGTATTCTTGATTTGAATCAGCTAGTTGACCAGA
GAATGAATCCATCTGCAAGCGATTACGCAGAGATTAAAAATGTTTTGGATGTGGCTTTGCTTT
GCACTTCAGCATTGCCTATCAATAGGCCTTCCATGAGAAGAGTTGTTGAATTGCTGAAGAATA
TCCCCTCCGCTCGTTCTAAAACAACGCATTAGCTTTTCACAGTTTCAAAAATGTGCTTGCTTGA
GTAACTTGATACTAGTGTAAAATGTAAAAGAATGTGTGAAATTAGGTGTCTGTTTAGGTCTGC
TTCATAGGTCAATTTAGTTTGCTTGCTGGGGTTTTAAACAAGTTCCTTGTTAGATACTCCAGTA
ATCCACATAGTAGCTTAACTAAAATATTGCTGACCTTGTAAAATTCATATTGTAACAGACTAA
AACATCGTGTTTTTGTTGCAATATATGAACTTTACTTTCATATACAA

5,-Upstream Sequence (1kb):

>Solyc02g091860.2.1.1
TGAACTATATCATAAAATATAGTCAATGTTATTATTATTTGACTCTAAAAAATTTAAATTATGA
TAATTTAAAATTAACGAAAATAATACTACTTTCTAAAAGAGTCTGTACATATTCAAAGAGAA
ATAGAGAGCAATTTAATGAAGTAACAATCATTATAACTAATGTAAGTATTAGCTAAACTTTTT
AAGCAATTAACGGCTCCATGTCCAAATATTCATGAAAAAAAATCAAGTTTGATGGATACTAT
TTTTCTAAAATTGGGAACACTTGCCAGGAAAATAAAAACAAAGCCAAAGAGGACGATACAA
TAAAACTTATGAAAAAGGATCTAAAATATTCTTAAATTATTAAATGGTACAAAATTATTCTCA
ATCCACTTATTTGGCTTCAAAATTTTCTCTCATTCACCTATTGACTTCAAATTATATAAATAATT
TAAATTTAAAACCGTTAAATAAGTGGTCAATTTTGAACCCAAAAGTGAATGACAAGAGTACT
TTGGAGTTAATAGGTGGGTGAGAAGGATATTTTAAAATCAATGGGTGGATGAAGAATAATTT
TGCATCATTTTCAATACTTTAAAAATATTTTAGATCCTTTTTCGTAAAACTTATCTTGATTGTTG
ATGCACAAATATTTTAGATTTCCAAAGTAATTTATTTTTCTTGGGTCTATATAATATGATATAA
TGATAGTAATAATAAGTTAATAACAAAATTTTTTTGTTCCAAATGTGATGATAACTATAAACC
TTCAACGGAGCATCATTAGNNNNNNNNNNNNNNNNNNNNNNNNNNNNNNNNNNNNN
NNNNNNNNNNNNNNNNNNNNNNNNNNNNNNNNNNNNNNNNNNNNNNNNNNNNNN
NNNNNNNNNNNNNNNNNNNNNNNNNNNNNNNNNNNNNNNNNNNNNNNNNNNNNN
NNNNNNNNNNNNNNNNNNNNNNNNNNNNNNNNNNNNNNNNNNNNNNNNNNNNNN
NNNNNNNNNNNNNNNNNNNNNNNNNN

>SlHSL6 ORF Solyc08g066270.1.1

Protein Sequence:

>Solyc08g066270.1.1
MEITTLTSSMSSLLYLSDFTAKIKRKKMESINEKMQLFFLIYFVLISPSLQLEQTVLLKLKQHWSDSEFL
QSWNSNSSECTWSGVWCIDDRVVTELHLGGKNITGTISSILCELKNLTFIDLSNNNISGIIPLSLKDCS
MLQHLDLSNNSLSDRIPGELFEMKQLLNLYLNGNMLSGEMPKEIASSQLKNLNLSENYLNGSIPEDI
GNLKNIVKLDMSHNSLSGSITNKLFQLHHLRHLSLSFNYLSSVIPDEMTLFSLYDMDLSHNQLTGSIP
RGFQYLPGLHALDLSYNQLSGDISQSIEHLRPRNTLKLCSNKFSGSISAEFVKLTYEENCFDESNLCSA
SKNLSVPSLPSCSSGDEVQKSSRPKHLIIIIPIVGFGVAIQLTWIFYMVRKHWWKTKKRNVKDDMKLI

SFQKLKVTTEGILCSLKDENIIGNGGSGKVYRVVIDQTGNTYAVKSIGHGGKSGGRNQKEFVAEVRT
LGSIRHNNIVKLMCCISSLDRKLLVYEYFEKQSLDKWLHGEKKAASPGQSSTPALDWRKRLNIAIGA
AQGLCYMHHHCTRAIIHRDIKSSNILLDSEFNAKISDFGLAKILSRRDDDPETASAIAGTFGYIAPEY
ASTFKVNVKTDIYSYGVVLLELTTGREPVLRDEQMNLAEWALQRYREGNSILDALDKEVMETSNLE
QMRSVFKLGLMCTGASPSGRPSMKEEWQWSSGARQGFNGTRQGTPLKWDRAGRGGSKLIFLEH

CDS Sequence:

>Solyc08g066270.1.1

ATGGAAATAACTACGTTGACTTCAAGTATGAGCTCTTTATTGTATTTAAGTGACTTCACTGCCA
AAATTAAAAGAAAAAAAATGGAAAGTATCAATGAAAAAATGCAATTGTTTTTCCTCATTTAC
TTCGTCCTAATTAGCCCAAGTTTACAGTTAGAACAGACCGTGTTGTTAAAATTGAAACAACAC
TGGAGCGATTCGGAGTTCCTCCAGTCATGGAATTCAAATTCTTCAGAATGCACTTGGTCTGGA
GTTTGGTGCATCGATGACAGGGTAGTCACAGAACTACATCTTGGAGGAAAAAACATAACCGG
AACAATCTCATCGATACTATGTGAGCTCAAGAACCTTACCTTCATTGATCTCTCCAACAATAA
TATTTCAGGAATCATACCACTAAGCCTGAAAGATTGCTCCATGTTACAACATTTGGATTTGTC
CAATAATTCATTGAGCGACCGGATTCCAGGTGAGTTGTTTGAGATGAAACAATTACTCAATTT
GTATCTTAATGGTAATATGTTGTCTGGTGAGATGCCAAAAGAAATAGCATCATCCCAGCTGA
AAAATCTTAATCTCTCTGAGAACTACCTGAACGGTTCGATACCAGAAGATATTGGTAACTTGA
AAAATATTGTGAAATTGGACATGTCACATAATTCTTTAAGTGGTTCAATTACTAACAAGTTGT
TTCAGTTGCATCATTTGCGTCACCTGTCGCTAAGTTTCAACTATTTGTCCAGTGTGATACCTGA
TGAAATGACCTTGTTTAGTTTGTATGATATGGATCTTTCACATAACCAATTGACTGGTTCTATA
CCAAGGGGATTTCAGTATTTACCTGGACTGCATGCTCTGGATTTGTCGTATAATCAGTTATCAG
GAGATATCTCGCAAAGTATAGAGCATCTCAGGCCTAGAAATACTTTAAAGCTTTGTTCCAAC
AAATTCTCAGGAAGCATTTCTGCTGAATTTGTGAAGTTAACATACGAAGAGAATTGTTTTGAC
GAGTCCAATCTTTGTTCTGCATCCAAGAACTTATCCGTACCTAGCCTACCAAGCTGTTCCTCTG
GTGATGAGGTTCAAAAATCTTCGAGACCAAAACACTTGATTATTATTATTCCTATTGTAGGAT
TTGGGGTAGCAATACAGTTAACATGGATCTTTTACATGGTCAGAAAGCATTGGTGGAAAACA
AAGAAGCGGAATGTTAAAGATGACATGAAGCTCATTTCATTTCAGAAGTTGAAGGTGACTAC
AGAAGGCATTTTGTGTAGCTTGAAAGACGAAAACATAATAGGAAATGGAGGATCAGGGAAG
GTCTATCGAGTTGTGATCGACCAAACAGGCAATACTTATGCTGTTAAAAGCATAGGTCATGG
GGGAAAATCAGGTGGAAGAAACCAAAAGGAGTTCGTGGCAGAAGTTAGAACACTTGGTAGC
ATTCGACACAACAACATTGTCAAGCTCATGTGTTGCATCTCAAGTTTAGACAGAAAGCTTCTA
GTCTATGAATACTTTGAGAAACAAAGCCTCGACAAATGGCTTCACGGAGAGAAAAAAGCAG
CATCACCAGGTCAAAGTAGTACCCCGGCCCTGGATTGGCGAAAGAGACTAAATATAGCCATT
GGTGCAGCTCAAGGACTATGCTATATGCACCATCATTGCACTCGAGCCATCATTCATAGAGA
CATTAAGTCCAGCAATATCCTTCTGGACTCAGAATTCAATGCAAAAATATCAGATTTTGGACT
AGCAAAAATACTATCCAGGCGGGATGACGATCCCGAGACAGCATCTGCTATTGCTGGAACAT
TTGGTTACATTGCACCAGAGTATGCCTCGACATTCAAAGTGAACGTAAAGACTGATATCTACA
GCTATGGAGTGGTGCTATTAGAATTGACAACAGGGAGAGAACCCGTTCTCAGGGATGAGCAA
ATGAATCTAGCAGAATGGGCGCTACAGCGTTACAGAGAGGGGAATTCCATTCTCGATGCCCT
CGATAAAGAAGTGATGGAAACAAGTAATTTGGAACAAATGAGAAGTGTTTTTAAACTAGGAT
TGATGTGTACAGGAGCATCACCATCTGGTAGGCCATCAATGAAGGAGGAATGGCAATGGAG
CAGCGGGGCAAGGCAAGGGTTTAACGGGACAAGACAGGGGACACCACTTAAATGGGATAG
GGCAGGGCGAGGCGGGTCAAAGCTAATATTCTTAGAACATTAA

Gene Sequence:

>Solyc08g066270.1.1
ATGGAAATAACTACGTTGACTTCAAGTATGAGCTCTTTATTGTATTTAAGTGACTTCACTGCCA
AAATTAAAAGAAAAAAAATGGAAAGTATCAATGAAAAAATGCAATTGTTTTTCCTCATTTAC
TTCGTCCTAATTAGCCCAAGTTTACAGTTAGAACAGACCGTGTTGTTAAAATTGAAACAACAC
TGGAGCGATTCGGAGTTCCTCCAGTCATGGAATTCAAATTCTTCAGAATGCACTTGGTCTGGA
GTTTGGTGCATCGATGACAGGGTAGTCACAGAACTACATCTTGGAGGAAAAAACATAACCGG
AACAATCTCATCGATACTATGTGAGCTCAAGAACCTTACCTTCATTGATCTCTCCAACAATAA
TATTTCAGGAATCATACCACTAAGCCTGAAAGATTGCTCCATGTTACAACATTTGGATTTGTC
CAATAATTCATTGAGCGACCGGATTCCAGGTGAGTTGTTTGAGATGAAACAATTACTCAATTT
GTATCTTAATGGTAATATGTTGTCTGGTGAGATGCCAAAAGAAATAGCATCATCCCAGCTGA
AAAATCTTAATCTCTCTGAGAACTACCTGAACGGTTCGATACCAGAAGATATTGGTAACTTGA
AAAATATTGTGAAATTGGACATGTCACATAATTCTTTAAGTGGTTCAATTACTAACAAGTTGT
TTCAGTTGCATCATTTGCGTCACCTGTCGCTAAGTTTCAACTATTTGTCCAGTGTGATACCTGA
TGAAATGACCTTGTTTAGTTTGTATGATATGGATCTTTCACATAACCAATTGACTGGTTCTATA
CCAAGGGGATTTCAGTATTTACCTGGACTGCATGCTCTGGATTTGTCGTATAATCAGTTATCAG
GAGATATCTCGCAAAGTATAGAGCATCTCAGGCCTAGAAATACTTTAAAGCTTTGTTCCAAC
AAATTCTCAGGAAGCATTTCTGCTGAATTTGTGAAGTTAACATACGAAGAGAATTGTTTTGAC
GAGTCCAATCTTTGTTCTGCATCCAAGAACTTATCCGTACCTAGCCTACCAAGCTGTTCCTCTG
GTGATGAGGTTCAAAAATCTTCGAGACCAAAACACTTGATTATTATTATTCCTATTGTAGGAT
TTGGGGTAGCAATACAGTTAACATGGATCTTTTACATGGTCAGAAAGCATTGGTGGAAAACA
AAGAAGCGGAATGTTAAAGATGACATGAAGCTCATTTCATTTCAGAAGTTGAAGGTGACTAC
AGAAGGCATTTTGTGTAGCTTGAAAGACGAAAACATAATAGGAAATGGAGGATCAGGGAAG
GTCTATCGAGTTGTGATCGACCAAACAGGCAATACTTATGCTGTTAAAAGCATAGGTCATGG
GGGAAAATCAGGTGGAAGAAACCAAAAGGAGTTCGTGGCAGAAGTTAGAACACTTGGTAGC
ATTCGACACAACAACATTGTCAAGCTCATGTGTTGCATCTCAAGTTTAGACAGAAAGCTTCTA
GTCTATGAATACTTTGAGAAACAAAGCCTCGACAAATGGCTTCACGGAGAGAAAAAAGCAG
CATCACCAGGTCAAAGTAGTACCCCGGCCCTGGATTGGCGAAAGAGACTAAATATAGCCATT
GGTGCAGCTCAAGGACTATGCTATATGCACCATCATTGCACTCGAGCCATCATTCATAGAGA
CATTAAGTCCAGCAATATCCTTCTGGACTCAGAATTCAATGCAAAAATATCAGATTTTGGACT
AGCAAAAATACTATCCAGGCGGGATGACGATCCCGAGACAGCATCTGCTATTGCTGGAACAT
TTGGTTACATTGCACCAGGTACATAACAGTTCAAACATTTATGAATATTCAATCTTCTAAAAT
CCAATTCTAATATATCAAATTTTAATATTTGGTCCTGACAGAGTATGCCTCGACATTCAAAGT
GAACGTAAAGACTGATATCTACAGCTATGGAGTGGTGCTATTAGAATTGACAACAGGGAGAG
AACCCGTTCTCAGGGATGAGCAAATGAATCTAGCAGAATGGGCGCTACAGCGTTACAGAGA
GGGGAATTCCATTCTCGATGCCCTCGATAAAGAAGTGATGGAAACAAGTAATTTGGAACAAA
TGAGAAGTGTTTTTAAACTAGGATTGATGTGTACAGGAGCATCACCATCTGGTAGGCCATCAA
TGAAGGAGGTTTGCTATGTTCTCCAAAGCTGCAGAGACTCTAATATTTGAGAGAGACGGACA
TAATTGTAAATAGAGATGATTAGAGTCTTTGATTATTTATGCAAATAGTATCTATTCATTTGCC
ATGAACTGGTTTTGAAGATTATAAAAAACTTCACGGTTCCTTGTGACTAATCCTATTTCAAGA
TTAAAATCTACATGATTTCTCATACCATATTTTACATGAAATTTGTCTGTATGACTCTCATTTTT
TTCTGTTAGCAAAGCATGTCTTCTACTTGTTTGCATCAGTAGTAATTTACAGTTACAAATGAAT
AGTATGCTTATATTATAAGTAGGAATGGCAATGGAGCAGCGGGGCAAGGCAAGGGTTTAACG
GGACAAGACAGGGGACACCACTTAAATGGGATAGGGCAGGGCGAGGCGGGTCAAAGCTAA
TATTCTTAGAACATTAA

5,-Upstream Sequence (1kb):
>Solyc08g066270.1.1
ATTACAAGATTCTTATAATCGAAACTGGGCGGAGCGGGACGGGATAGGGTAAATTCACCACA
GGGGACAGAACAGGGCAATTTGGGAACGGAACGGGGCGACGAGGTAACGGTAAGGATGAA
TATTATATTCGTATGATAAGTAAACATTGACATTTAATGATGACTACGTTTGTTCATCTTCTGT
ACGAAACGATTGTCTTTTTTTACTCTCAGTATGTCTGTTTAAAGTACATTTTATACCATACTCTT
TAGTACATCTAAAATTAGAACTTTATCCTAATCAGTGTTCCTTGGCACTTCAAAAAATATTAG
AAGTTTTGGTCAAGTACCGTTTACTTATCTATGATAAACGTATTTATTAGTTTCTGAGATTAGT
AGAGATAAATGTTAATACAGGCAGAGAGAGTTTATAATCTCAGAGACGTCGAAACCTCTTGT
ATCGTTTGGAGGAAGTAACTACCGGATGGTCTACCACTACGAGGACATGTGTAGTTAGGATC
AAATTTTTGTGAAGAGTAAACAAGGTTTAATGAACAAAGGTAGTGAAGAAATAGCTCCCGTA
GCTCTTACCTTAAGGGGAGAGACATTGCGACATCGCGGGTAAGACGATCTAAGTAAACGAGT
AGGGACTCTTGCCCAAGAGAGGGACAACAGTTAAGATTATCGTGGTGAGGTATCGACATCTA
TAGTCAGAAATGCAAGTGAAACTTACAGCTCCGTATGAGACAGTCCTGGTTTATAATTTTAAA
CTATATAATCTTAACCTAAAATCTTCTAACTTATAAGTATTTACAAACTTGACAATACATGGA
CCACGTTACATTGGTTTACAAGGTCGTTATCGTCTACGACAGAGCCCTAGCAGTAGGGCGGA
CCTATCATAAAAACGATCAGGTTTTAGACTATAAAAACGTAACTTAAGACTCAGGTCTTCCTA
TAACGACCTGAATTACAGAGATACTTACTACCGAGCTCACGTTACTACCACGTATATCGTA

>SlHSL7 ORF Solyc08g066320.2.1
Protein Sequence:
>Solyc08g066320.2.1
MIQASSKLFWLNMQIFFLISLSFQETLQFSGTERSILLNMKQHWSDSEFFQSWNSNSSECTWPGVWCI
DDRVVTELHLGGKNITGTISSILCELKNLTFIDLSNNNISGIIPLSLKDCSMLQHLDLSNNSLSDRIPGE
LFEMKQLLNLYLNGNMLSGEMPKEIASSQLKNLNLSENYLNGSIPEDIGNLKNIVKLDMSHNSLSG
SITNKLFQLHHLRHLSLSFNYLSSVIPDEMNLFSLYDMDLSHNQLTGSIPRGFQYLPGLHALDLSYNQ
LSGDISQSIEHLRPRNTLKLCSNKFSGSISAEFVKLTYEENCFDESNLCSASKNLSVPSLPNCSSGDEVQ
KSSRPKHLIIIIPIVGFGVAIQLTWIFYMVRKHWWKTKKRNVKDDIKFISFQKLKVTTEDILCSLKDEN
IIGNGGSGKVYRVVIDQTGSTYAVKSIGHGGKSGGRPQKEFLAEVRTLGSIRHNNIVKLMCCISSLDR
KLLVYEYFEKQSLDKWLHGEKRAVSPGQSSTPALDWRKRLNIATGAAQGLSYMHHDCTRPIIHRDI
KSSNILLDSEFNAKIADFGLAKILSRRDDNPETASAIAGTFGYIAPEYASTFRVNIKTDIYSFGVVLLEL
TTGRQPILREEQMNLAQWAQQRYKDGNYIVEALDEEIMETSNVEQMRDVFKLGLMCTGASPSSRP
SMKEVCNILQSLRDPIF
CDS Sequence:
>Solyc08g066320.2.1
ATGATCCAAGCAAGCTCTAAACTTTTTTGGCTCAATATGCAAATCTTCTTCCTAATTAGCCTGT
CTTTCCAAGAAACCTTACAATTTTCAGGCACGGAACGGTCCATATTACTAAACATGAAACAA
CACTGGAGCGATTCGGAGTTTTTCCAGTCGTGGAATTCAAATTCTTCAGAATGCACTTGGCCT
GGAGTTTGGTGCATCGATGACAGGGTAGTCACAGAACTACATCTTGGAGGAAAAAACATAA
CCGGAACAATTTCATCGATACTATGTGAGCTCAAGAACCTTACCTTCATTGATCTCTCCAACA
ATAATATTTCAGGAATCATACCACTAAGCCTGAAAGATTGCTCCATGTTACAACATTTGGATT
TGTCCAATAATTCATTGAGCGACCGGATTCCAGGTGAGTTGTTTGAGATGAAACAATTACTCA
ATTTGTATCTTAATGGTAATATGTTGTCTGGTGAGATGCCAAAAGAAATAGCATCATCCCAGC
TGAAAAATCTTAATCTCTCTGAGAACTACCTGAACGGTTCGATACCAGAAGATATTGGTAACT

TGAAAAATATTGTGAAACTGGACATGTCACATAATTCTTTAAGTGGTTCAATTACTAACAAGT
TGTTTCAGTTGCATCATTTGCGTCACCTGTCCCTAAGTTTCAACTATTTGTCCAGTGTGATACCT
GATGAAATGAACTTGTTTAGTTTGTATGATATGGATCTTTCACATAACCAATTGACTGGTTCTA
TACCAAGGGGATTTCAGTATTTACCTGGACTGCATGCTCTGGATTTGTCATATAATCAGTTATC
AGGAGATATCTCGCAAAGTATAGAGCATCTCAGGCCTAGAAATACTTTAAAGCTTTGTTCCA
ACAAATTCTCAGGAAGCATTTCTGCTGAATTTGTGAAGTTAACATACGAAGAGAATTGTTTTG
ACGAGTCCAATCTTTGTTCTGCATCCAAGAACTTATCCGTACCTAGCCTACCAAACTGTTCCTC
TGGTGATGAGGTTCAAAAATCTTCAAGACCAAAACACTTGATTATTATTATTCCTATTGTAGG
ATTTGGGGTAGCAATACAGTTAACATGGATCTTTTACATGGTCAGAAAGCATTGGTGGAAAA
CAAAGAAGCGGAATGTTAAAGATGACATAAAGTTCATTTCATTTCAAAAGTTGAAGGTGACT
ACAGAAGACATTTTGTGTAGCTTGAAAGACGAAAACATAATAGGAAATGGAGGATCAGGCA
AGGTCTATCGAGTTGTGATCGATCAAACAGGCAGTACTTATGCTGTTAAAAGCATAGGTCATG
GGGGAAAATCAGGTGGAAGACCCCAAAAGGAGTTCCTGGCAGAAGTTAGAACACTTGGTAG
CATTCGACACAACAACATTGTCAAGCTCATGTGTTGCATCTCAAGTTTAGACAGAAAGCTTCT
AGTCTATGAATACTTTGAGAAACAAAGCCTCGACAAATGGCTTCACGGAGAGAAAAGAGCA
GTATCACCAGGTCAAAGTAGTACCCCTGCCCTGGATTGGCGAAAGAGACTAAATATAGCCAC
TGGTGCAGCTCAAGGACTCAGCTATATGCACCATGACTGTACTCGACCCATCATTCATAGAG
ACATTAAGTCCAGCAATATCCTTCTGGACTCAGAATTCAATGCAAAAATAGCAGATTTTGGAT
TAGCAAAAATACTATCGAGGAGAGATGATAATCCTGAGACAGCTTCTGCTATTGCTGGAACA
TTTGGTTACATTGCACCAGAGTATGCCTCGACATTCAGAGTGAATATAAAGACAGATATATAT
AGCTTCGGAGTGGTGCTATTAGAATTAACAACAGGGAGACAACCCATTCTTAGAGAGGAGCA
AATGAATCTAGCACAATGGGCTCAACAGCGTTACAAAGATGGGAATTACATTGTTGAGGCCC
TTGATGAAGAAATCATGGAAACAAGTAATGTGGAACAAATGAGAGATGTTTTTAAACTAGGA
CTAATGTGTACCGGAGCATCACCATCTAGTAGGCCGTCAATGAAGGAAGTCTGCAACATTCTT
CAAAGTCTCAGAGACCCCATTTTTTGA

Gene Sequence:

>Solyc08g066320.2.1

GACTTGACAGACTTCATTATCAAATGAAAAGAAGCACATGAAGGAAAATAAAACATCAAAT
TAACATGATCCAAGCAAGCTCTAAACTTTTTTGGCTCAATATGCAAATCTTCTTCCTAATTAGC
CTGTCTTTCCAAGAAACCTTACAATTTTCAGGCACGGAACGGTCCATATTACTAAACATGAAA
CAACACTGGAGCGATTCGGAGTTTTTCCAGTCGTGGAATTCAAATTCTTCAGAATGCACTTGG
CCTGGAGTTTGGTGCATCGATGACAGGGTAGTCACAGAACTACATCTTGGAGGAAAAAACAT
AACCGGAACAATTTCATCGATACTATGTGAGCTCAAGAACCTTACCTTCATTGATCTCTCCAA
CAATAATATTTCAGGAATCATACCACTAAGCCTGAAAGATTGCTCCATGTTACAACATTTGGA
TTTGTCCAATAATTCATTGAGCGACCGGATTCCAGGTGAGTTGTTTGAGATGAAACAATTACT
CAATTTGTATCTTAATGGTAATATGTTGTCTGGTGAGATGCCAAAAGAAATAGCATCATCCCA
GCTGAAAAATCTTAATCTCTCTGAGAACTACCTGAACGGTTCGATACCAGAAGATATTGGTA
ACTTGAAAAATATTGTGAAACTGGACATGTCACATAATTCTTTAAGTGGTTCAATTACTAACA
AGTTGTTTCAGTTGCATCATTTGCGTCACCTGTCCCTAAGTTTCAACTATTTGTCCAGTGTGATA
CCTGATGAAATGAACTTGTTTAGTTTGTATGATATGGATCTTTCACATAACCAATTGACTGGTT
CTATACCAAGGGGATTTCAGTATTTACCTGGACTGCATGCTCTGGATTTGTCATATAATCAGTT
ATCAGGAGATATCTCGCAAAGTATAGAGCATCTCAGGCCTAGAAATACTTTAAAGCTTTGTTC
CAACAAATTCTCAGGAAGCATTTCTGCTGAATTTGTGAAGTTAACATACGAAGAGAATTGTTT
TGACGAGTCCAATCTTTGTTCTGCATCCAAGAACTTATCCGTACCTAGCCTACCAAACTGTTC

CTCTGGTGATGAGGTTCAAAAATCTTCAAGACCAAAACACTTGATTATTATTATTCCTATTGTA
GGATTTGGGGTAGCAATACAGTTAACATGGATCTTTTACATGGTCAGAAAGCATTGGTGGAA
AACAAAGAAGCGGAATGTTAAAGATGACATAAAGTTCATTTCATTTCAAAAGTTGAAGGTGA
CTACAGAAGACATTTTGTGTAGCTTGAAAGACGAAAACATAATAGGAAATGGAGGATCAGG
CAAGGTCTATCGAGTTGTGATCGATCAAACAGGCAGTACTTATGCTGTTAAAAGCATAGGTC
ATGGGGGAAAATCAGGTGGAAGACCCCAAAAGGAGTTCCTGGCAGAAGTTAGAACACTTGG
TAGCATTCGACACAACAACATTGTCAAGCTCATGTGTTGCATCTCAAGTTTAGACAGAAAGCT
TCTAGTCTATGAATACTTTGAGAAACAAAGCCTCGACAAATGGCTTCACGGAGAGAAAAGAG
CAGTATCACCAGGTCAAAGTAGTACCCCTGCCCTGGATTGGCGAAAGAGACTAAATATAGCC
ACTGGTGCAGCTCAAGGACTCAGCTATATGCACCATGACTGTACTCGACCCATCATTCATAGA
GACATTAAGTCCAGCAATATCCTTCTGGACTCAGAATTCAATGCAAAAATAGCAGATTTTGG
ATTAGCAAAAATACTATCGAGGAGAGATGATAATCCTGAGACAGCTTCTGCTATTGCTGGAA
CATTTGGTTACATTGCACCAGGTATATTACAGTTCAAATATTTATCGTACCTCAAGAATTCTCA
AAATAATTTATCCAACTGTAATATTAAATTTTAATGTTTGATCCTCTCAGAGTATGCCTCGACA
TTCAGAGTGAATATAAAGACAGATATATATAGCTTCGGAGTGGTGCTATTAGAATTAACAAC
AGGGAGACAACCCATTCTTAGAGAGGAGCAAATGAATCTAGCACAATGGGCTCAACAGCGT
TACAAAGATGGGAATTACATTGTTGAGGCCCTTGATGAAGAAATCATGGAAACAAGTAATGT
GGAACAAATGAGAGATGTTTTTAAACTAGGACTAATGTGTACCGGAGCATCACCATCTAGTA
GGCCGTCAATGAAGGAAGTCTGCAACATTCTTCAAAGTCTCAGAGACCCCATTTTTTGAAAG
AATAGAATGTTTAGCCTTACATAG

5,-Upstream Sequence (1kb):

>Solyc08g066320.2.1.1

ATTAGCTTTGAAGAAACCATGTATCATTATCATACAACACAAGTCAGTCAACATGTTATGAAT
ATTGTAGCCGACACTGAAAGGGAAATGCTAGCAAAGTGTTGACTTGTTAAAAGAAAAGAAA
CAAAAGTTGACTTGACTGACCTTTGTGGCCAAGAAATAAACAGCAAAGCAAGAAAGATCTTT
GCCATTTCCAGAAAAATGATGATGGGAAAGGAAACAATTTTTTACCAACTATTTTTCAATTTT
GTCGTGTTTGGTTTGACTTAAATGTTTTTGAAAATATTTTTCAAATCAACAAAATGACTTACCT
CGAATACATTTTTCCAAAACTATTTTTCAACATCGCACCTCAACTTTCCACTTTAACTCAAGTC
TTGGTTGCCAATTCTCGATACTAATGCGAGATACAAGTCTTGATTCTTTATTTGAGACTTGATT
TTCGATCACAAATCGAGACCCCACTCTCGAGTGTCGGATCGGGTCACTGTTGCAGTCGAAATC
CAAATTTGAATGTTGTGTCTAAGATTATAAAAACATAAATAATCTAATCATTCATAAACTCTT
TATCAATTATTTACCATCGTAAAATTACGTGATTTTCACACATAGTAGGAATACATACTCGAA
GAAGTCATAGTATACTTTGGAAATCTTCACTAAAATTAAATGAAAATCTTTTCTTTATAGATCT
TGTTTTTCTCCTTTACCTTACGTTTCATGAATAATTTTGATAAATAAGACTATACTTTACGAAG
AGAATGAGGGGACCATTTAATGGATTCCCACTTTGCATCATCCTGAATAGTGGGTATGGTGTA
TAGTTTTAAAACTACCAGGTCTCCATCTTTATGGGTAGTTTAAAGTTACTACAGGTCTAGGATT
ATACTTATATTAATTTTAAACCAAAATTATAATTATGTGGGTCATAGAAATTTACAATATATC
AGGTCAGGTTTCGAGGTCGGGTGTCGGAGTTGGATCCCGAGTCAGCTGTTGG
