# Supplementary material for: Involvement of IDA-HAE Module in Natural Development of Tomato Flower Abscission
Source: Plants (Basel). 2023 Jan 1;12(1):185. doi: 10.3390/plants12010185 (PMC9823658; doi:10.3390/plants12010185)
Supplement: Supplementary file 1 [file plants-12-00185-s001.zip › Supplementary Table 1. Primers used in the study_11-28-2022.pdf]

Supplementary Table S1. Sequence of primers used for qRT-PCR analysis

	Gene	Primers	Annealing Temperature °C
1.	*SlEF1b*	F - TTGGTCCCAGTTGGATACGGAAT<br>R -AGGTTCCTCTGTTAGACGTTCCTCAAT	58.2<br>58.0
2.	*SlTAPG2*	F - AGTGAAAACATGGGCAAGGCCA<br>R - TTATATCGCTTATCTTTATACCAGAACCCTG	60.2<br>58.1
3.	*SlTAPG4*	F - GCATTGGAAGCTTAGGTTGGGAATC<br>R - CACCATTCTCAGTACTTGTGAAGCTAACCA	60.0<br>60.9
4.	*SlIDA*	F - TCTTCTTCAAAAACCCTTTATTTATCAAGC<br>R - CCTCTACCATCATAATTCTCCCAAATCTTG	58.2<br>60.5
5.	*SlIDL1*	F - AACTACAAGAGATGTTGAAAAAAAATCACAAC<br>R - GGTAATAAGGGCTTAACATTAACAACTTGTG	58.1<br>58.3
6.	*SlIDL2*	F - GGAGATGGAAAAAATGAGCATAAAAAACAC<br>R - CCTTGAAAAATTGTGTGTGACTTGCAC	60.5<br>59.8
7.	*SlIDL3*	F - GAAATGGCTTATTCTGCTAATTCCAAAAC<br>R - GTAGGTGGGCATGTAGTACCATGTCC	59.0<br>58.6
8.	*SlIDL4*	F - CTTGTTCTTGGCTATGCTTCTTCAGTG<br>R - TGTATAAGCGTCTTCATATTTTGGAGATGG	58.7<br>59.9
9.	*SlIDL5*	F - TTGGCCATTTTTGTCATGGTT<br>R - TTCTTGATGGACCAGAAGCTG	55.5<br>59.4
10.	*SlIDL6*	F - CGTTCATGTGCTAGACCGTT<br>R - CACGTTCCCTTCTCACGTTC	58.4<br>60.5
11.	*SlIDL7*	F - CCATTACCTCCATCTGCTCC<br>R - GGGTGAAACTCTCAACAACG	60.5<br>58.4
12.	*SlHSL*	F - ATGTTGCAAGAAGTAGGTGGTGGA<br>R - CACTTCCTTGATCAGATGCTTCTTC	58.0<br>57.0
13.	*SlHSL1*	F - CCCCGATGAGAAAAGTCGTAAA<br>R - ACATTTCCTTGATCTGATGCGTC	57.0<br>57.0
14.	*SlHSL2*	F - CTCAATGCACAGAGTGGTGAAAA<br>R - TGACTTTGGAAAGTACTGAGGGGA	56.0<br>58.0
15.	*SlHSL3*	F - TGAATGAAGTCGTTCAGCTGCTA<br>R - CAAATCATATATGCTCTTTGGTGGC	56.0<br>57.0
16.	*SlHSL4*	F - TAGCGGGAACAACAGCGAAAA<br>R - CACTGGAAATTATTAGACCATCATCAC	59.0<br>56.0
17.	*SlHSL5*	F - TGTTTTGGATGTGGCTTTGCTTTG<br>R - TTTTAGAACGAGCGGAGGGGATA	62.0<br>60.0
18.	*SlHSL6*	F - GATGCCCTCGATAAAGAAGTGATG<br>R - ATTGATGGCCTACCAGATGGTGAT	58.0<br>59.0
19.	*SlHSL7*	F - ACAGGGAGACAACCCATTCTTAGA<br>R - TTCCCATCTTTGTAACGCTGTTG	57.0<br>58.0
